# The coordination of alkali–metal nickelates to organic π-systems: synthetic, structural and spectroscopic insights†

**DOI:** 10.1039/d4dt00889h

**Published:** 2024-04-03

**Authors:** Andryj M. Borys, Luca Vedani, Eva Hevia

**Affiliations:** a Departement für Chemie, Biochemie und Pharmacie, Universität Bern 3012 Bern Switzerland andryj.borys-smith@unibe.ch eva.hevia@unibe.ch

## Abstract

Low-valent nickelates have recently been shown to be key intermediates in challenging cross-coupling reactions using aryl ethers as electrophiles. Key for the success of these transformations is the activation of the substrate through π-coordination to the nickelate intermediate, however there is still limited knowledge about the fundamental structure and coordination chemistry of these heterobimetallic complexes. Herein, we report the synthesis, structures, and spectroscopic analysis of a diverse family of alkali–metal nickelates derived from phenyl-alkali–metal reagents and Ni(*ttt*-CDT), where *ttt*-CDT = *trans*,*trans*,*trans*-1,5,9-cyclododecatriene. The co-complexation of PhLi with Ni(*ttt*-CDT) was found to yield 1 : 1, 2 : 1 or 4 : 2 lithium nickelates depending on the stoichiometry and reaction conditions employed. The high lability of the *ttt*-CDT ligand enables facile ligand exchange with an assorted series of organic π-acceptors, ranging from polyaromatic hydrocarbons to ketones, imines and nitriles. For anthracene and phenanthrene, a homologous series of Li, Na and K nickelates could be obtained, which lead to different structural motifs or degrees of aggregation in the solid-state spanning solvated monomers to complex polymeric arrangements. For π-extended systems such as perylene or coronene, competing single-electron-transfer to give the corresponding radical anions was observed, illustrating the highly reducing nature of the alkali–metal nickelates. X-ray crystallographic analysis and NMR spectroscopy of the phenyl-alkali–metal nickelates reveal extreme back-bonding from Ni(0) to the organic π-acceptors due to strong σ-donation from the carbanionic ligands.

## Abstract 2

By assessing of the co-complexation chemistry of PhM (M = Li, Na or K) with Ni(*ttt*-CDT) in the presence of organic π-acceptors, new structural and spectroscopic insights into the coordination chemistry of alkali–metal nickelates have been gained.

## Graphical abstract

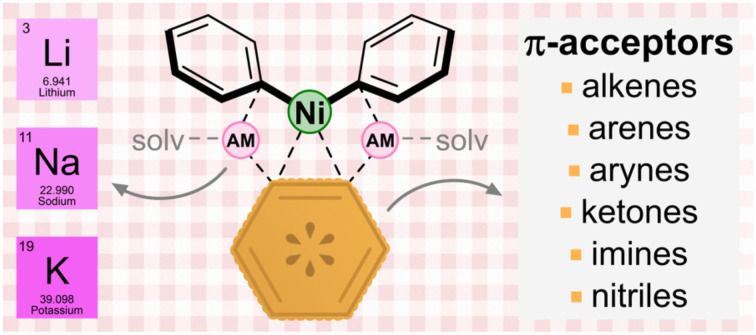

## Introduction

**Scheme 1:**
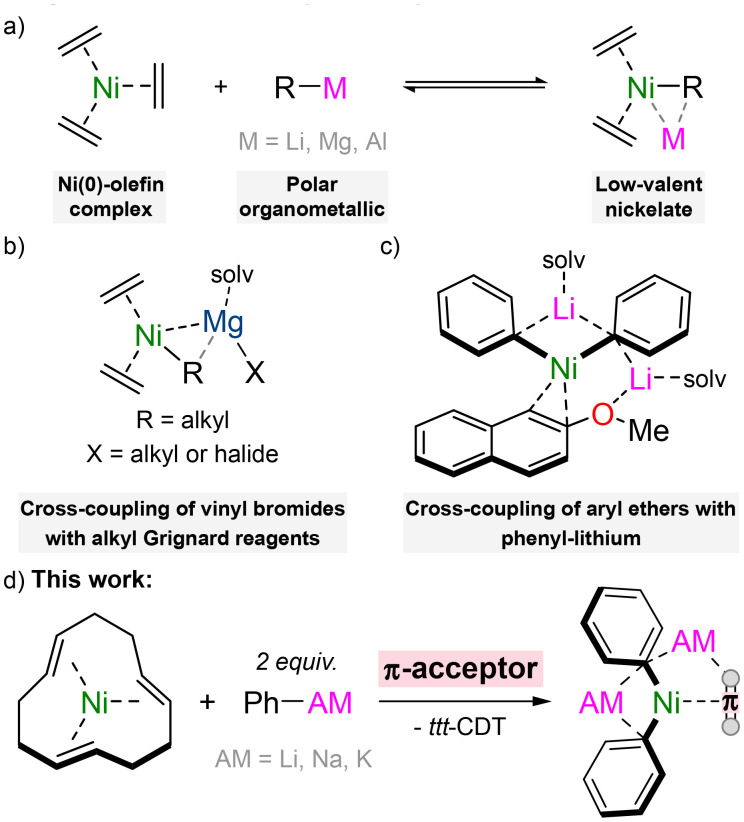
(a) Co-complexation of Ni(0)-olefin complexes with polar organometallics to give low-valent nickelates. (b) Proposed magnesium nickelate intermediate in the low-temperature cross-coupling of vinyl bromides with alkyl Grignard reagents. (c) Proposed lithium nickelate intermediate in the cross-coupling or aryl ethers with phenyl-lithium. (d) This work – coordination of 2 : 1 phenyl-alkali–metal nickelates to organic π-acceptors.

The coordination chemistry of transition-metals has fascinated chemists for over 150 years^1^ and a plethora of ligands with varying donor strength, accepting properties, denticity, and countless other features have been reported to date.^2,3^ One unique class of transition-metal complex is low-valent nickelates,^4,5^ and these are typically accessed by treating a Ni(0)-olefin^6,7^ precursor [*e.g.* Ni(C_2_H_4_)_3_] with a polar organometallic reagent (*e.g.* organolithiums, Scheme 1a). In these complexes, the carbanion from the polar organometallic reagent can be viewed as a strong σ-donating ligand which coordinates to the Ni(0) centre. Stabilisation of these electron-rich Ni(0) complexes is presented in the form of a complementary π-accepting ligand, which may be the parent olefin from which the nickelate is derived, an external π-acceptor, or from the carbanion itself. Low-valent nickelates were widely investigated in the 1970s and 80s,^4^ and the high reactivity of these systems is exemplified by their ability to activate small molecules such as dinitrogen.^8–10^ Nevertheless, they remained dormant in the literature for several decades and it is only in recent years that a renaissance in the field has emerged.^5^ This has been sparked by mechanistic studies which have demonstrated that low-valent nickelates are overlooked intermediates that facilitate challenging cross-coupling reactions.^11,12^ Cornella has shown that highly-reduced or simple 16-electron Ni(0)-olefin complexes catalyse the low-temperature C(sp^2^)–C(sp^3^) Kumada cross-coupling of vinyl bromides with alkyl Grignard reagents (Scheme 1b),^13,14^ whilst our group and others have employed Ni(COD)_2_ for the cross-coupling of aryl ethers with phenyl-lithium (Scheme 1c).^15–17^ In the latter case, the coordination ability of both nickel and lithium is crucial towards activating the substrate and facilitating smooth C_aryl_–OMe bond cleavage. Despite these recent catalytic and mechanistic advances however, there is still limited fundamental knowledge into the coordination, structure, and bonding of low-valent nickelates, but these insights may provide essential information towards the rational design of new complexes with untapped catalytic potential.

There are three main variables which influence the synthesis, properties, and reactivity of low-valent nickelates – (i) the donor ligand; (ii) the secondary metal; and (iii) the π-accepting ligand. The donor ligand is derived directly from the polar organometallic reagent and is formally a carbanionic centre, although hydrides (*e.g.* LiAlH_4_)^18,19^ and phosphides (*e.g.* LiPPh_2_)^20^ can also fall under this definition. The hybridisation of the donor ligand will influence the properties and examples of sp^3^ (alkyl),^20–22^ sp^2^ (aryl)^15,17,23,24^ and sp (acetylide)^25,26^ derived nickelates have been reported. Additionally, the stoichiometry of the donor ligand employed is crucial and this has been shown to vary from 1 : 1 up to 4 : 1, with the ability for many additional molecules of donor ligand to co-complex in the structure without direct coordination to Ni(0).^26^ The identity of the secondary metal (*e.g.* Li, Mg, Al) cannot be overlooked, since this will dictate how readily formal transfer of the carbanion centre to Ni(0) occurs depending on the electropositivity and Lewis acidity of the secondary metal. The solvation of the secondary metal can also be manipulated to give contacted or solvent-separated species, or to influence the overall aggregation of the low-valent nickelate. Late-stage exchange of the secondary metal is also possible,^24^ which may grant access to previously inaccessible heterobimetallic complexes. The final, and less well studied feature of low-valent nickelates is the π-accepting ligand. Most examples to date have employed simple olefin ligands such as ethylene, COD (1,5-cyclooctadiene) or *ttt*-CDT (*trans*,*trans*,*trans*-1,5,9-cyclododecatriene), but it has been shown that these can often be displaced by other π-accepting ligands such as alkynes, arynes, arenes or even N_2_.^8–10^ Herein, we systematically explore and expand the scope of π-accepting ligands using low-valent nickelates derived from Ni(*ttt*-CDT) and phenyl-alkali–metal reagents (Scheme 1d).

## Results and discussion

### Co-complexation chemistry of Ni(*ttt*-CDT) and PhLi

**Fig. 1:**
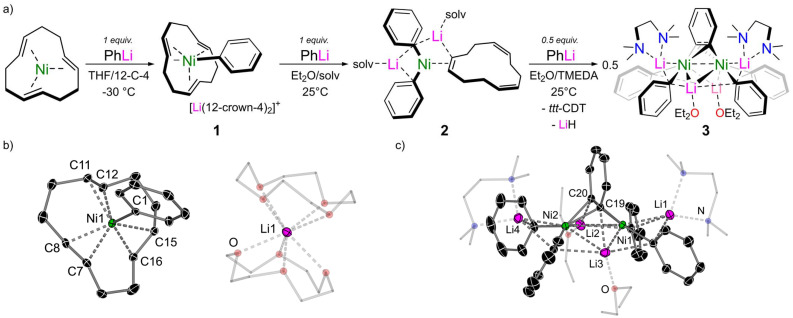
(a) Synthesis of 1 : 1 lithium nickelate (1), 2 : 1 lithium nickelate (2), and 4 : 2 lithium nickelate benzyne-complex (3). (b) Molecular structure of [(*ttt*-CDT)NiPh][Li(12-crown-4)_2_] 1. Thermal ellipsoids shown at 30% probability. Hydrogen atoms omitted and 12-crown-4 shown as wireframes for clarity. (c) Molecular structure of Li_4_(TMEDA)_2_(Et_2_O)_2_Ph_4_Ni_2_(μ;η^2^;η^2^-C_6_H_4_), 3. Thermal ellipsoids shown at 30% probability. Hydrogen atoms omitted and coordinated TMEDA and Et_2_O as wireframes for clarity.

The co-complexation chemistry of Ni(COD)_2_ and PhLi has been systematically studied and yields a structurally diverse family of lithium nickelates depending on the stoichiometry and reaction conditions employed.^15,23^ The co-complexation chemistry of Ni(*ttt*-CDT) with PhLi has also been documented by Jonas and Krüger, but since experimental and analytical details are very limited,^4^ we decided to first investigate this more systematically. The addition of one equivalent of PhLi to a THF solution of Ni(*ttt*-CDT) at −30 °C leads to a distinct colour change from red to yellow. The addition of excess 12-crown-4 enables the isolation of the 1 : 1 lithium nickelate, [(*ttt*-CDT)NiPh][Li(12-crown-4)_2_] 1, in 59% crystalline yield (Fig. 1a). The solid-state structure of 1 reveals a solvent-separated ion pair in which the lithium cation (Li1) is sequestered by two molecules of 12-crown-4 (Fig. 1b). The Ni1–C1 distance is 2.024(1) Å which is noticeably longer than Cornella's 1 : 1 lithium nickelate Li(TMEDA)PhNi(C_2_H_4_)_2_ [1.963(1) Å]^13^ and all other 2 : 1 lithium nickelates derived from PhLi.^15,17,23,24^ Compared to Ni(*ttt*-CDT) in which the Ni(0) centre sits perfectly co-planar within the cyclododecatriene ligand,^27^ the Ni-centre in 1 is approximately 0.66 Å above the mean plane defined by the 12 carbon atoms. This leads to longer Ni-olefin distances when compared to Ni(*ttt*-CDT), but only marginal increase in the C=C distances [1.384(3)–1.386(3) Å]. The low temperature ^13^C{^1^H} NMR spectrum of 1 displays a downfield (deshielded) resonance at *δ* 195.3 ppm for the *ipso*-carbon (C1), indicating a highly polarised carbon–nickel bond. Four signals are observed for the coordinated *ttt*-CDT ligand, which are less shielded when compared to [(*ttt*-CDT)NiCH_3_][Li(THF)_4_].^21,28^ Whilst complex 1 can be isolated, it is unstable above −20 °C and decomposes within minutes in solution at room temperature. This finding contrasts with phenyl-lithium nickelates derived from Ni(COD)_2_ where the corresponding 1 : 1 lithium nickelate can only be spectroscopically detected at high concentrations and readily redistributes back to Ni(COD)_2_ and a 2 : 1 lithium nickelate.^15^

The addition of 2 equivalents of PhLi to a Et_2_O solution of Ni(*ttt*-CDT) leads to the clean formation of Li_2_(solv)_*n*_Ph_2_Ni(η^2^-CDT) (2), however all attempts to grow single crystals suitable for X-ray diffraction were unsuccessful due to its high solubility and instability due to ligand lability (*vide infra*). The TMEDA solvate could be isolated as a microcrystalline solid in low yields (16%) however and displays similar spectroscopic features to the previously reported COD analogue, Li_2_(solv)_*n*_Ph_2_Ni(η^2^-COD).^15^ Complex 2 slowly reacts with an additional 0.5 equivalents of PhLi to give a dinickel-benzyne complex, Li_4_(TMEDA)_2_(Et_2_O)_2_Ph_4_Ni_2_(μ;η^2^;η^2^-C_6_H_4_) (3). We have previously reported a closely related dinickel-benzyne complex Li_6_(Et_2_O)_4_Ph_6_Ni_2_(μ;η^2^;η^2^-C_6_H_4_),^23^ which is formed by the addition of a large excess of PhLi to Ni(COD)_2_. This species was proposed to form by LiH elimination from the third PhLi ligand, preventing the formation of the hypothetical 3 : 1 lithium nickelate “Li_3_(solv)_*n*_Ph_3_Ni”.^29^ Formally, this species can be viewed as possessing a neutral benzyne π-ligand, however the highly reducing nature of the lithium nickelate leads to overall reduction to give a {C_6_H_4_}^2−^ ligand, as exemplified by the long C–C bond length of 1.449(6) Å.^23^ In the solid-state structure of 3 (Fig. 1c), no additional PhLi co-complexation is observed and the overall structural and spectroscopic features are comparable to previously reported examples, including its elongated C19–C20 bond length of 1.424(2) Å.

### Polyaromatic hydrocarbons as π-ligands to alkali–metal nickelates

**Fig. 2:**
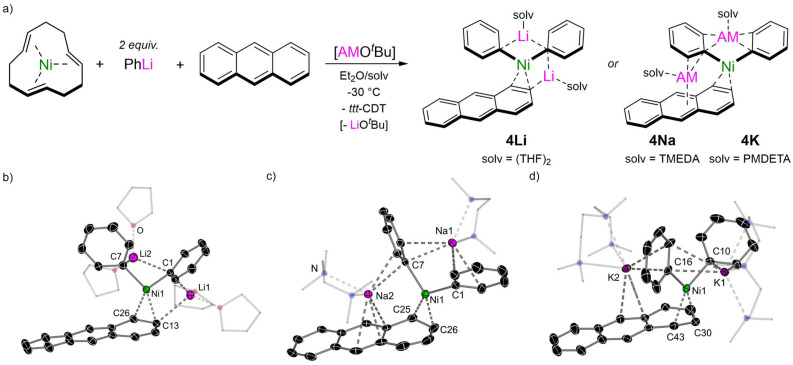
(a) Synthesis of 2 : 1 phenyl-alkali–metal nickelate η^2^-anthracene complexes 4Li, 4Na and 4K. (b) Molecular structure of 4Li. Thermal ellipsoids shown at 30% probability. Hydrogen atoms omitted and coordinated THF shown as wireframes for clarity. (c) Molecular structure of 4Na. Thermal ellipsoids shown at 30% probability. Hydrogen atoms omitted and coordinated TMEDA shown as wireframes for clarity. (d) Molecular structure of 4K. Thermal ellipsoids shown at 30% probability. Hydrogen atoms omitted and coordinated PMDETA shown as wireframes for clarity.

Jonas and Krüger have documented that the *ttt*-CDT ligand in 2 : 1 lithium nickelate 2 can be displaced by other π-accepting ligands^4^ and we have recently exploited this strategy to isolate Li_2_(TMEDA)_2_Ph_2_Ni(η^2^-naphthalene).^17^ Extending this methodology, the corresponding anthracene analogue Li_2_(THF)_4_Ph_2_Ni(η^2^-anthracene) 4Li can be readily accessed as its THF solvate in 52% crystalline yield (Fig. 2a). *In situ* alkali–metal exchange using NaO^*t*^Bu or KO^*t*^Bu (2.5 equivalents) in the presence of a suitable polydendate donor gave the corresponding sodium Na_2_(TMEDA)_2_Ph_2_Ni(η^2^-anthracene) 4Na and potassium K_2_(PMDETA)_2_Ph_2_Ni(η^2^-anthracene) 4K analogues, as black/green crystalline solids in 42% and 24% yield respectively. The solid-state structure of 4Li is comparable to Li_2_(TMEDA)_2_Ph_2_Ni(η^2^-naphthalene)^17^ and Li_2_(solv)_*n*_Ph_2_Ni(η^2^-COD),^15^ and features two distinct Li environments; Li2 is coordinated to both *ipso*-carbons (C1 and C7) whilst Li1 is coordinated to one *ipso*-carbon [C1⋯Li1 2.252(4) Å] and to one carbon of the coordinated anthracene [C13⋯Li1 2.484(3) Å]. The C13–C26 distance is 1.462(2) Å, which is considerably longer than in free anthracene [1.352(3)–1.356(3) Å]^30^ and other L_*n*_Ni(η^2^-anthracene) complexes bearing trialkylphosphine ligands [1.423–1.427(4) Å],^31,32^ illustrating strong back-donation from the electron-rich Ni-centre. The solid-state structures of 4Na and 4K display similar bond metrics (C=C and Ni–C bond lengths) to 4Li but differ primarily in the coordination of the alkali–metal cations (Fig. 2b and c), a feature that has been observed for alkali–metal main-group metalate complexes.^33–35^ One sodium (Na1) or potassium (K1) cation is coordinated to two *ipso*-carbons (C1 and C7) but have additional interactions to *ortho*-carbons of both phenyl substituents. The second alkali–metal cation (Na2 and K2) coordinates to an *ipso*-carbon (C7) and *ortho*-carbon of one phenyl-substituent, and also coordinates in an η^3^-motif to the central six-membered ring of anthracene. This illustrates the softer character of sodium and potassium which prefer contacts to the arene rings, whilst lithium prefers more localised electrostatic interactions. The ^1^H NMR spectra of complexes 4Li, 4Na and 4K display upfield shifted resonances for the coordinated anthracene at *δ* 4.40 (4Li), *δ* 4.58 and 4.42 (4Na) and *δ* 4.49 and 4.24 (4K), which are considerably shielded compared to (^i^Pr_3_P)_2_Ni(η^2^-anthracene) [*δ* 5.32 and 5.55].^32^

**Fig. 3:**
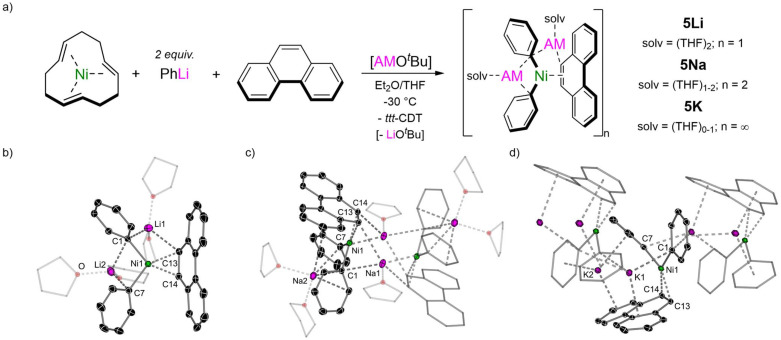
(a) Synthesis of 2 : 1 phenyl-alkali–metal nickelate η^2^-phenanthrene complexes 5Li, 5Na and 5K. (b) Molecular structure of 5Li. Thermal ellipsoids shown at 30% probability. Hydrogen atoms omitted and coordinated THF shown as wireframes for clarity. (c) Molecular structure of 5Na. Thermal ellipsoids shown at 30% probability. Hydrogen atoms omitted and coordinated THF shown as wireframes for clarity. (d) Molecular structure of 5K. Thermal ellipsoids shown at 30% probability. Hydrogen atoms and coordinated THF on K2 omitted for clarity.

Switching to phenanthrene as the π-accepting ligand, another polyaromatic hydrocarbon consisting of three fused benzene rings which is a non-linear isomer of anthracene, a complete series of 2 : 1 phenyl-alkali–metal nickelate η^2^-phenanthrene complexes 5Li, 5Na and 5K could also be obtained, all as their THF solvates (Fig. 3a). The lithium congener Li_2_(THF)_4_Ph_2_Ni(η^2^-phenanthrene) 5Li was previously documented by Jonas and Krüger^4^ and the solid-state structure (Fig. 3b) shows a solvated monomer which shares similar features to other 2 : 1 phenyl-lithium nickelate complexes.^15,17,23,24^ The sodium analogue 5Na is dimeric in the solid-state (Fig. 3c), akin to other 2 : 1 phenyl-sodium nickelates that have been reported to date.^23,36,37^ Finally, the potassium analogue 5K is polymeric in the solid-state (Fig. 3d) and propagates along the crystallographic *b* axis through numerous K⋯π-arene interactions to the phenyl substituents and coordinated phenanthrene ligand. Unlike 5Li and 5Na in which the phenanthrene ligand is essentially planar, the phenanthrene ligand in 5K is distorted away from planarity [14.6° deviation of mean planes defined by outer six-membered rings] and has considerable torsion [10.9(4)°]. In all complexes, the Ni(0) coordinates to the exposed 9,10-position of phenanthrene (labelled as C13 and C14) which is elongated relative to free phenanthrene [*cf.* 1.373 Å;^38^5Li 1.453(2) Å; 5Na 1.443(5) or 1.452(3) Å; 5K 1.447(4) Å]. In the ^1^H NMR spectra, the H13/14 resonances are observed at *δ* 2.77 (5Li), 3.05 (5Na) and 3.09 (5K), whilst in the ^13^C{^1^H} NMR spectra, the C13/14 resonances are observed at *δ* 39.0 (5Li), 38.9 (5Na) and 40.5 (5K). These resonances are significantly shielded (upfield shifted) relative to free phenanthrene [^1^H: *δ* 7.74; ^13^C{^1^H}: *δ* 127.8], indicating very strong back-donation from the Ni(0)-centre into the π-accepting ligand. Despite showing higher degrees of aggregation in the solid-state, ^1^H DOSY (diffusion ordered spectroscopy) NMR studies indicate that 5Na and 5K are both solvated monomers in THF-d_8_ solution (see Fig. S3 and S4†).

**Fig. 4:**
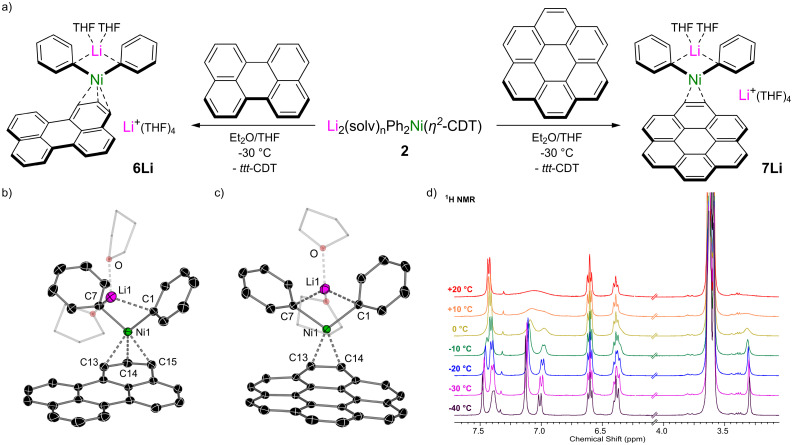
(a) Synthesis of 2 : 1 phenyl-lithium nickelate η^3^-perylene complex 6Li and η^2^-coronene complex 7Li. (b) Molecular structure of 6Li (anion only). Thermal ellipsoids shown at 30% probability. Hydrogen atoms omitted and coordinated THF shown as wireframes for clarity. (c) Molecular structure of 7Li (anion only). Thermal ellipsoids shown at 30% probability. Hydrogen atoms omitted and coordinated THF shown as wireframes for clarity. (d) Stacked ^1^H NMR spectra of 7Li at variable temperatures.

We next moved on to π-extended polyaromatic hydrocarbons to further assess how the identity of the π-accepting ligand influences the structure and properties of alkali–metal nickelates. Treatment of an *in situ* prepared solution of Li_2_(solv)_*n*_Ph_2_Ni(η^2^-CDT) 2 with 1 equivalent of perylene leads to an immediate colour change, from which lustrous black crystals of [Li(THF)_2_Ph_2_Ni(η^3^-perylene)][Li(THF)_4_] 6Li could be isolated in 71% yield (Fig. 4a). The solid-state structure of 6Li (Fig. 4b) reveals a pseudo-solvent-separated ion pair in which one lithium cation (Li1) is coordinated to both *ipso*-carbons (C1 and C7) whilst the second lithium cation is coordinated to four molecules of THF and is sequestered away from the lithium nickelate anion. The Ni-centre coordinates to the perylene ligand in a pseudo-η^3^-motif – stronger binding to C14 and C15 is observed however, as evidenced by shorter Ni–C distances [1.977(2) and 2.088(2) Å *vs.* 2.171(2) for Ni1–C13] and longer C–C distances [1.413(3) Å for C14–C15 *vs.* 1.401(3) for C13–C14]. The perylene ligand is slightly distorted away from planarity, with torsion angles across the bay-positions ranging from 2.5(3) to 5.5(3)°. Isolated samples of 6Li were found to be contaminated with a paramagnetic impurity identified as the perylene radical anion (see Fig. S5† for EPR spectrum).^39^ Moreover, attempts to prepare the sodium and potassium analogues *via* alkali–metal exchange with AMO^*t*^Bu led exclusively to the formation of the corresponding perylene radical anions, as confirmed by EPR spectroscopy and single-crystal X-ray diffraction (see Fig. S6, S7 and S18†), and the target alkali–metal nickelates could not be isolated. This suggests that the formation of the radical anion first proceeds through the targeted alkali–metal nickelates and not only illustrates the highly reducing nature of alkali–metal nickelates [*cf.* one-electron reduction potential of perylene = −1.98 V],^40^ but also suggests that the identity of the alkali–metal cation influences the reducing strength. Oxidised Ni(i) or Ni(ii) species are proposed to be nickel-containing by-products of the reaction, but attempts to identify these by NMR or EPR spectroscopy were unsuccessful. Extending the conjugation of π-accepting ligands even further, the treatment of Li_2_(solv)_*n*_Ph_2_Ni(η^2^-*ttt*-CDT) 2 with 1 equivalent of coronene (a.k.a. superbenzene) gave the corresponding lithium nickelate [Li(THF)_2_Ph_2_Ni(η^2^-coronene)][Li(THF)_4_] 7Li as a black crystalline solid. The solid-state structure of 7Li (Fig. 4c) shows a pseudo-solvent-separated ion pair, akin to 6Li. The observation of this structural motif for perylene and coronene indicates that these π-accepting ligands are better at distributing electron density and delocalising negative charge. In 7Li, the Ni-centre coordinates in a η^2^-motif to a peripheral “double bond” causing significant elongation for C13–C14 to 1.460(2) Å – in contrast, the five-remaining peripheral “double bonds” have distances ranging from 1.353(3)–1.377(2) Å. At room temperature, only a single very broad resonance is observed around *δ* 7.00 in the ^1^H NMR spectrum for the coordinated coronene ligand (Fig. 4d), indicative of exchange processes in which Ni coordinates/decoordinates to all six C=C bonds equally. Cooling down to −40 °C however leads to splitting of the resonances into well-defined signals and the appearance of a sharp singlet at *δ* 3.30 for the position coordinated to Ni (^13^C resonance for C13/14 at *δ* 42.3). Similarly, the ^7^Li NMR spectrum of 7Li at room temperature displays a sharp singlet at *δ* 0.17, which splits into two broad signals at *δ* 0.52 and 0.00 upon cooling to −40 °C (see Fig. S2†). Consistent with the lower reduction potential of coronene (−2.36 V),^40^ only traces of the coronene radical anion^41,42^ (see Fig. S8†) are observed during the synthesis of potassium nickelate K_2_(DME)_4_Ph_2_Ni(η^2^-coronene) 7K (see Fig. S20† for molecular structure).

**Scheme 2:**
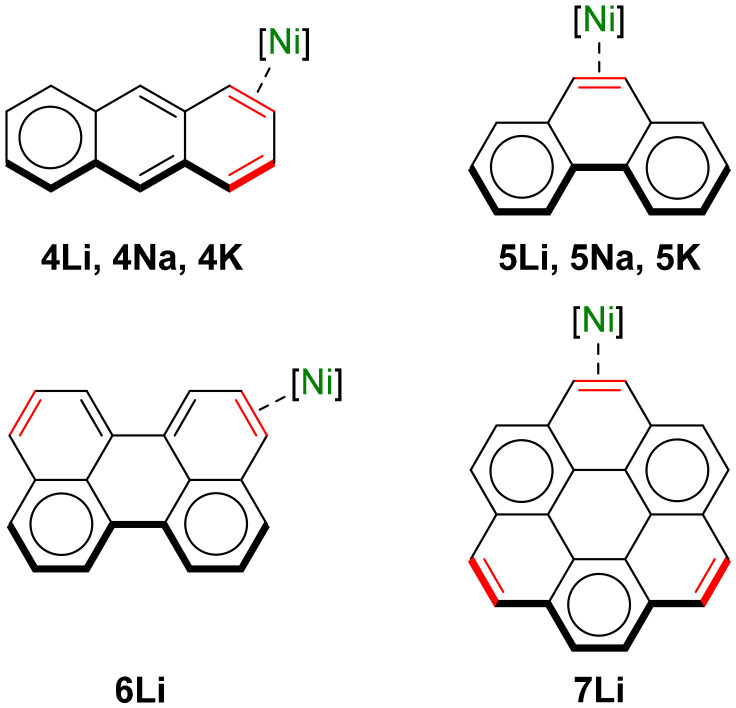
Clar structures of anthracene, phenanthrene, perylene and coronene and their respective coordination to Ni(0) in the 2 : 1 phenyl-alkali–metal nickelate complexes.

The polyaromatic hydrocarbons explored (anthracene, phenanthrene, perylene and coronene) have numerous different sites in which a transition-metal could hypothetically coordinate, and we therefore aimed to justify the coordination modes observed for the diverse series of 2 : 1 phenyl-alkali–metal nickelate complexes. Polyaromatic hydrocarbons can have several resonance forms which are typically depicted by their Kekulé structures (*i.e.* containing alternating single and double bonds). An alternate depiction is the Clar structure, which groups six π-electrons into an aromatic π-sextet and is represented by a circle.^43^ According to Clar's rules, the resonance structure of benzenoid polyaromatic hydrocarbons which contains the most aromatic π-sextets is the most important in characterising its chemical and physical properties. Interestingly, in all nickelate complexes reported herein, the Ni-centre coordinates to “exposed double bonds”^44^ (shown in red in Scheme 2) that are established in the Clar structure since these possess the greatest π-accepting ability, which is necessary to attenuate the high electron density at the Ni-centre. Supporting this hypothesis, no evidence of coordination is observed with benzene or biphenyl, benzenoid aromatic compounds in which no isolated double bonds are present in their Clar structures.

### Heteroatom-based π-acceptors

**Scheme 3:**
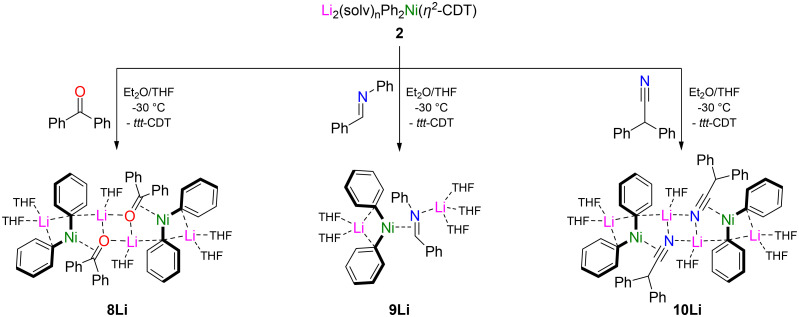
Synthesis of [Li_2_(THF)_3_Ph_2_Ni(η^2^-Ph_2_CO)]_2_ (8Li), Li_2_(THF)_5_Ph_2_Ni(η^2^-PhCH=NPh) (9Li) and [Li_2_(THF)_3_Ph_2_Ni(η^2^-Ph_2_CHCN)]_2_ (10Li).

We next turned our attention to unsaturated organic compounds containing heteroatoms which have the ability to act as a σ-donor, as well as a π-acceptor and π-donor. Utilising the same synthetic strategy, the treatment of *in situ* prepared solution of Li_2_(solv)_*n*_Ph_2_Ni(η^2^-CDT) 2 with 1 equivalent of benzophenone gave an intensely coloured solution from which purple crystals of [Li_2_(THF)_3_Ph_2_Ni(η^2^-Ph_2_CO)]_2_ (8Li) were isolated in 62% yield (Scheme 3, left). The solid-state structure (see Fig. S21† for full structure) reveals a dimeric motif with a Li_2_O_2_ core, a feature which is common in alkali–metal alkoxide structures.^45–47^ The Ni-centre coordinates in a η^2^-motif to the carbonyl fragment resulting in significant elongation of the C–O bond to 1.389(2)–1.393(2) Å [*cf.* 1.223(2) Å in free Ph_2_CO].^48^ Noticeably, this bond length is considerably longer than other L_*n*_Ni(η^2^-benzophenone) complexes [1.331(3)–1.353(6) Å]^49–52^ and is even approaching that observed in the benzophenone dianion [1.406 Å for (Ph_2_C–O)Li_2_].^53^ Complex 8Li could be treated with KO^*t*^Bu in the presence of DME (dimethoxyethane) to give [K_2_(DME)_3_Ph_2_Ni(η^2^-Ph_2_CO)]_2_ (8K) as deep blue crystals in 50% yield, and showed the same dimeric aggregation in the solid-state and comparable structural parameters to 8Li (see Fig. S22† for full structure).

**Fig. 5:**
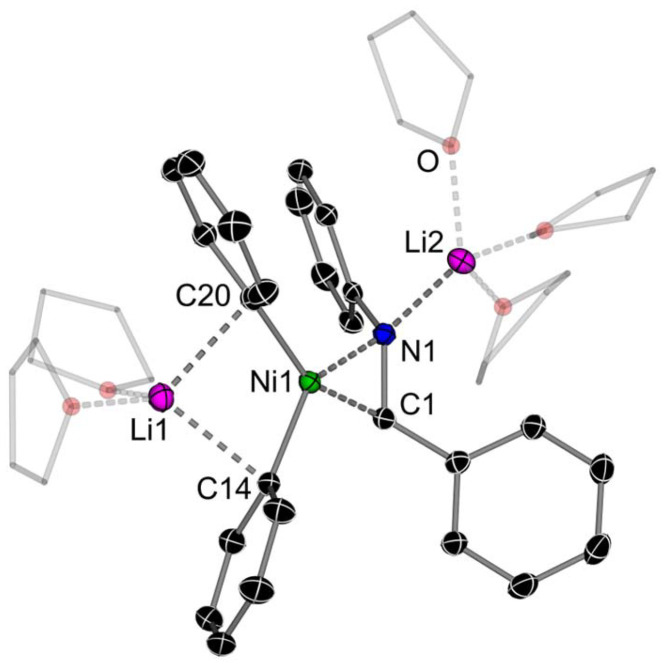
Molecular structure of 9Li. Thermal ellipsoids shown at 30% probability. Hydrogen atoms omitted and coordinated THF shown as wireframes for clarity.

The reaction of Li_2_(solv)_*n*_Ph_2_Ni(η^2^-CDT) 2 with 1 equivalent of *N*-benzylideneaniline afforded Li_2_(THF)_5_Ph_2_Ni(η^2^-PhCH=NPh) (9Li) as a red crystalline solid in 50% yield (Scheme 3). The solid-state structure (Fig. 5) reveals a monomeric structure in which one lithium cation (Li1) is coordinated to the two *ipso*-carbons (C14 and C20) whilst the second lithium cation (Li2) is coordinated to N1 and solvated by three molecules of THF. The C1–N1 bond length [1.419(2) Å] is again elongated significantly when compared to free *N*-benzylideneaniline [1.260(3) Å]^54^ and other reported L_*n*_Ni(η^2^-PhCH=NPh) complexes [1.368(3) Å].^55^ Consistent with strong back-donation from Ni, the C1–H resonance in 9Li is observed at *δ* 3.81 in the ^1^H NMR spectrum, considerably shielded (upfield shifted) with respect to free *N*-benzylideneaniline [*cf. δ* 8.50 ppm].

**Fig. 6:**
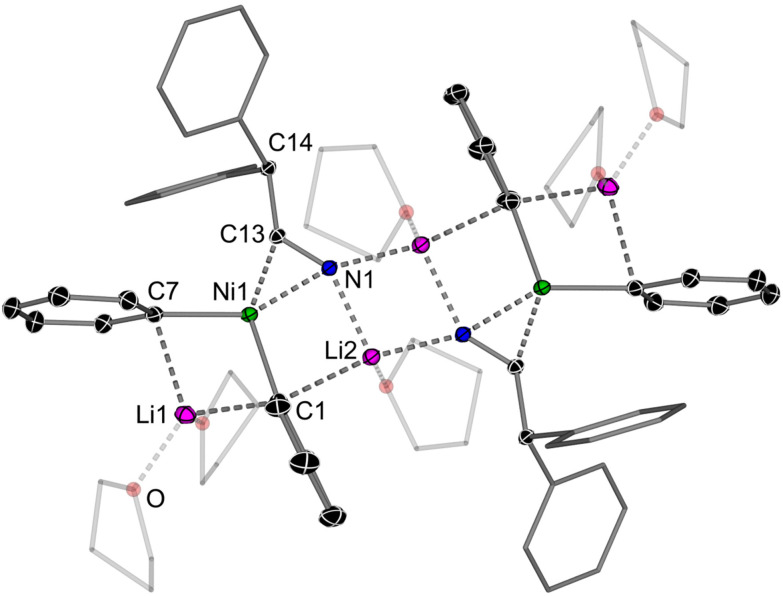
Molecular structure of 10Li. Thermal ellipsoids shown at 30% probability. Hydrogen atoms omitted and coordinated THF shown as wireframes for clarity.

Extending the scope of π-acceptors to nitriles, the treatment of Li_2_(solv)_*n*_Ph_2_Ni(η^2^-*ttt*-CDT) 2 with 1 equivalent of diphenylacetonitrile afforded yellow crystals of [Li_2_(THF)_3_Ph_2_Ni(η^2^-Ph_2_CHCN)]_2_ (10Li) in a 42% yield (Scheme 3). The solid-state structure of 10Li reveals a dimeric motif (Fig. 6) akin to 8Li, with characteristic elongation of the C13–N1 bond [1.242(2) Å *vs.* 1.147(2) Å for Ph_2_CHCN].^56^ In addition, the C14–C13–N1 unit is significantly distorted away from linearity [128.9(1)° *vs.* 177.9(1)° for Ph_2_CHCN],^56^ a feature that is observed for the coordination of triply bonded acetylenes to alkali–metal nickelates.^24,25^ The isolation of complexes 8Li, 9Li and 10Li is particularly surprising given that these unsaturated substrates readily react with PhLi *via* nucleophilic addition,^57–59^ or α-deprotonation in the case of diphenylacetonitrile.^56^ No evidence of competitive nucleophilic addition of Ph–M (where M = Li or Ni) or reduction is observed in the synthesis of 8Li and 9Li, and these complexes are stable for several days at room temperature in solution. We propose that coordination of these C=O or C=N substrates to Ni decreases their electrophilicity and thus tendency towards nucleophilic addition. Contrastingly, 10Li can only be isolated in low yields and is thermally sensitive, indicating that competing pathways or onward decomposition is facile.

To further investigate the coordination chemistry and quantify the back-donation of alkali–metal nickelates, we next targeted the corresponding 1 : 1 and 2 : 1 lithium nickelate carbonyl complexes Li(solv)_*n*_PhNi(CO)_3_ and Li_2_(solv)_*n*_Ph_2_Ni(CO)_2_. Despite repeated efforts and numerous synthetic routes however, all attempts to isolate or characterise these targets were unsuccessful. Treatment of *in situ* prepared solutions of [(*ttt*-CDT)NiPh][Li(solv)_*n*_] 1 or Li_2_(solv)_*n*_Ph_2_Ni(η^2^-*ttt*-CDT) 2 with 1 atmosphere of carbon monoxide initially leads to a colour change to deep red at −196 °C, but rapid decomposition occurs upon warming to give an intractable mixture of compounds alongside nickel black. Direct synthesis from Ni(CO)_4_ and 1 or 2 equivalents of PhLi also gave intractable mixtures even at low temperatures, and only traces of Li_2_(THF)_4_Ph_4_Ni(ii) could be identified,^60^ suggesting that CO may be reduced under these conditions. Ligand exchange by treatment of (Ph_3_P)_2_Ni(CO)_2_ with two equivalents of PhLi also gave a mixture of species, from which the mixed phosphine/phosphide complex Li(THF)_3_(PPh_3_)_2_(CO)_3_Ni_2_(μ-PPh_2_) (11Li) was identified as the major species by ^31^P NMR spectroscopy and single crystal X-ray diffraction (see Fig. S25† for molecular structure). Similar complexes have previously been documented by treatment of (Ph_3_P)_2_Ni(CO)_2_ with potassium metal,^61^ whilst the formation of 11Li likely originates from Ni-mediated P–C bond cleavage.^51,62–64^ The lack of selectivity and temperature sensitivity of these reactions is unsurprising given that phenyl-lithium itself reacts unselectively with carbon monoxide to give a mixture of organic species including benzophenone, benzoin and diphenylacetophenone upon acidic workup.^65^ Examples of nickelate-carbonyl complexes have been documented by Wilke, Pörschke and Jonas, but these are reported to be extremely temperature sensitive and readily react further to give acyl-lithium nickelate complexes.^21,28^ The synthetic challenge and toxicity risks associated with the preparation of nickel-carbonyl complexes has been addressed through the introduction of computed electronic parameters (CEP) for a series of known and hypothetical L–Ni(CO)_3_ complexes.^66^ This demonstrated that carbanionic ligands such as Ph^−^ are much stronger donors compared to neutral ligands such as phosphines and N-heterocyclic carbenes, consistent with the structural and spectroscopic features observed in isolated phenyl-alkali–metal nickelate complexes reported herein.

## Conclusions

In conclusion, this work has explored the co-complexation of PhLi with Ni(*ttt*-CDT), and the rich coordination chemistry of 2 : 1 phenyl-alkali–metal-nickelates towards a range of organic π-accepting ligands. As exemplified by the homologous series of Li, Na and K nickelates obtained for anthracene and phenanthrene, the overall nickelate core remains very similar whilst significant differences in the coordination environment of the alkali–metal cation are observed, with the π-affinity of the larger and softer potassium cation being most prevalent. The alkali–metal effects also lead to differences in reducing capability, as evidenced by the spectroscopic observation of perylene and coronene radical anions when preparing potassium nickelates. For polyaromatic hydrocarbons, we found that the strong back-donation from the electron-rich Ni centre reveals the Clar structure which contains exposed double bonds with the greatest π-accepting ability. The alkali–metal nickelate coordination chemistry can also be extended to heteroatom based π-acceptors such as ketones, imines, and nitriles, where surprisingly no formal reduction to radical anions or dianions is observed. Given the emerging role of alkali–metal nickelates in catalysis, we believe that these coordination effects will have important mechanistic implications, which can be leveraged towards designing new transformations. We are currently exploiting these coordination and alkali–metal effects for further bond activation chemistry.

## Conflicts of interest

There are no conflicts to declare.

## Supplementary Material

DT-053-D4DT00889H-s001

DT-053-D4DT00889H-s002
