# Supplementary material for: The coordination of alkali–metal nickelates to organic π-systems: synthetic, structural and spectroscopic insights
Source: Dalton Trans. 2024 Apr 3;53(19):8382–90. doi: 10.1039/d4dt00889h (PMC11091858; doi:10.1039/d4dt00889h)
Supplement: DT-053-D4DT00889H-s001 [file DT-053-D4DT00889H-s001.pdf]

Electronic Supplementary Material (ESI) for Dalton Transactions.
This journal is © The Royal Society of Chemistry 2024

Supporting Information

# The Coordination of Alkali-Metal Nickelates to Organic π-Systems: Synthetic, Structural and Spectroscopic Insights

Andryj M. Borys,[a*] Luca Vedani,[a] Eva Hevia[a*]

[a] Departement für Chemie, Biochemie und Pharmacie, Universität Bern, 3012 Bern, Switzerland

## Table of Contents


Experimental ........ 4

General Considerations ........ 4

Synthesis and Purification of PhLi ........ 4

Synthesis of Alkali-Metal Nickelates ........ 5

Synthesis of [(*ttt*-CDT)NiPh][Li(12-crown-4)$_2$] (**1**) ........ 5

Synthesis of $Li_2(TMEDA)_2Ph_2Ni(\eta^2$-CDT) (**2**) ........ 5

Synthesis of $Li_4(TMEDA)_2(Et_2O)_2Ph_4Ni_2(\mu;\eta^2;\eta^2-C_6H_4)$ (**3**) ........ 6

Synthesis of $Li_2(THF)_4Ph_2Ni(\eta^2$-anthracene) (**4Li**) ........ 6

Synthesis of $Na_2(TMEDA)_2Ph_2Ni(\eta^2$-anthracene) (**4Na**) ........ 7

Synthesis of $K_2(PMDETA)_2Ph_2Ni(\eta^2$-anthracene) (**4K**) ........ 8

Synthesis of $Li_2(THF)_4Ph_2Ni(\eta^2$-phenanthrene) (**5Li**) ........ 8

Synthesis of $[Na_2(THF)_3Ph_2Ni(\eta^2$-phenanthrene)$]_2$ (**5Na**) ........ 9

Synthesis of $[K_2(THF)_1Ph_2Ni(\eta^2$-phenanthrene)$]_\infty$ (**5K**) ........ 10

Synthesis of $[Li(THF)_2Ph_2Ni(\eta^3$-perylene)][Li(THF)$_4$] (**6Li**) ........ 10

Synthesis of $[Li(THF)_2Ph_2Ni(\eta^2$-coronene)][Li(THF)$_4$] (**7Li**) ........ 11

Synthesis of $K_2(DME)_4Ph_2Ni(\eta^2$-coronene) (**7K**) ........ 11

Synthesis of $[Li_2(THF)_3Ph_2Ni(\eta^2-Ph_2CO)]_2$ (**8Li**) ........ 12

Synthesis of $[K_2(DME)_3Ph_2Ni(\eta^2-Ph_2CO)]_2$ (**8K**) ........ 12

Synthesis of $Li_2(THF)_5Ph_2Ni(\eta^2$-PhCH=NPh) (**9Li**) ........ 13

Synthesis of $[Li_2(THF)_3Ph_2Ni(\eta^2-Ph_2CHCN)]_2$ (1**0L**i) ........ 14

Synthesis of $Li(THF)_3(PPh_3)_2(CO)_3Ni_2(\mu-PPh_2)$ (**11Li**) ........ 14

Variable Temperature NMR Spectroscopy ........ 15

$[Li(THF)_2Ph_2Ni(\eta^2$-coronene)][Li(THF)$_4$] (**7Li**) ........ 15

DOSY NMR Spectroscopy ........ 16

$[Na_2(THF)_3Ph_2Ni(\eta^2$-phenanthrene)$]_2$ (**5Na**) ........ 16

$[K_2(THF)_1Ph_2Ni(\eta^2$-phenanthrene)$]_\infty$ (**5K**) ........ 17

EPR Spectroscopy ........ 18

X-Ray Crystallography ........ 20

Solid-State Structure of [(*ttt*-CDT)NiPh][Li(12-crown-4)$_2$] (**1**) ........ 28

Solid-State Structure of $Li_4(TMEDA)_2(Et_2O)_2Ph_4Ni_2(\mu;\eta^2;\eta^2-C_6H_4)$ (**3**) ........ 28

Solid-State Structure of $Li_2(THF)_4Ph_2Ni(\eta^2$-anthracene) (**4Li**) ........ 29

Solid-State Structure of $Na_2(TMEDA)_2Ph_2Ni(\eta^2$-anthracene) (**4Na**) ........ 29

Solid-State Structure of $K_2(PMDETA)_2Ph_2Ni(\eta^2$-anthracene) (**4K**) ........ 30

Solid-State Structure of $Li_2(THF)_4Ph_2Ni(\eta^2$-phenanthrene) (**5Li**) ........ 30

Solid-State Structure of $[Na_2(THF)_3Ph_2Ni(\eta^2$-phenanthrene)$]_2$ (**5Na**) ........ 31

Solid-State Structure of $[K_2(THF)_1Ph_2Ni(\eta^2$-phenanthrene)$]_\infty$ (**5K**) ........ 31

Solid-State Structure of $[Li(THF)_2Ph_2Ni(\eta^3$-perylene)][Li(THF)$_4$] (**6Li**) ........ 32

Solid-State Structure of [perylene][K(DME)$_4$] ........ 32

Solid-State Structure of $[Li(THF)_2Ph_2Ni(\eta^2$-coronene)][Li(THF)$_4$] (**7Li**) ........ 33

Solid-State Structure of $K_2(DME)_4Ph_2Ni(\eta^2$-coronene) (**7K**) ........ 34

Solid-State Structure of $[Li_2(THF)_3Ph_2Ni(\eta^2\text{-}Ph_2CO)]_2$ (**8Li**) ........ 34
Solid-State Structure of $[K_2(DME)_3Ph_2Ni(\eta^2\text{-}Ph_2CO)]_2$ (**8K**) ........ 35
Solid-State Structure of $Li_2(THF)_5Ph_2Ni(\eta^2\text{-}PhCH{=}NPh)$ (**9Li**) ........ 36
Solid-State Structure of $[Li_2(THF)_3Ph_2Ni(\eta^2\text{-}Ph_2CHCN)]_2$ (**10Li**) ........ 36
Solid-State Structure of $Li(THF)_3(PPh_3)_2(CO)_3Ni_2(\mu\text{-}PPh_2)$ (**11Li**) ........ 37
NMR Spectra of Reported Compounds ........ 38
Reference Spectra ........ 61
*In Situ* Synthesis and Characterisation of Imine Addition Product, $Ph_2CHN(Li)Ph$ ........ 64
*In Situ* Synthesis and Characterisation of Lithiated Diphenylketenimine, $Ph_2C{=}C{=}N\text{–}Li$ ........ 66
References ........ 68

## Experimental

### General Considerations

All manipulations were carried out under an inert atmosphere of argon using standard Schlenk line or glovebox techniques (MBraun UNILab Pro ECO, <0.1 ppm H2O and O2).[1,2] All manipulations, except for the synthesis of PhLi or Ni(*ttt*-CDT), must avoid the use of Teflon-coated stir bars or cannulae, and glass-coated stir bars should be used. THF was dried and distilled from Na/benzophenone and stored over 4 Å molecular sieves, then further dried and vacuum distilled over $NaK_{2.8}$ or a sodium mirror. Hexane, pentane, Et2O and toluene were pre-dried using a MBraun MBSPS 5, then further dried and vacuum distilled over $NaK_{2.8}$ or a sodium mirror, and stored over 4 Å molecular sieves. THF-d8 and C6D6 were dried and vacuum distilled over $NaK_{2.8}$ and stored over 4 Å molecular sieves. TMEDA and PMDETA were distilled over CaH2 and stored over 4 Å molecular sieves. DME was dried and distilled from Na/benzophenone and stored over 4 Å molecular sieves.12-crown-4 was degassed by three freeze-pump-thaw cycles and stored over 4 Å molecular sieves. Ni(*ttt*-CDT) was prepared according to literature procedures and purified by vacuum sublimation.[3] Anthracene and phenanthrene were dried and sublimed *in vacuo* prior to use. All other reagents were used as supplied and dried *in vacuo* prior to use. Glovebox filtrations to separate insoluble solids were performed using 0.2 µm polypropylene (PP) syringe filters. Low temperature glovebox reactions were maintained using custom-made aluminium cooling blocks that were stored in the glovebox freezer (-30 °C) prior to use.

NMR spectra were recorded on Bruker Avance III HD 300 MHz or 400 MHz spectrometers at 300 K unless otherwise specified. $^{1}$H NMR spectra were referenced internally to the corresponding residual *protio* solvent peaks. CHN elemental microanalyses were performed on a Flash 2000 Organic Elemental Analyser (Thermo Scientific). Samples were prepared and crimped in tin capsules in an argon filled glovebox. Analyses were performed in triplicate, and reference standards (e.g. nicotinamide) were measured prior to use as controls. EPR spectra were recorded at room temperature in 1.5 mm inset capillaries on a Bruker EMXnano instrument.

### Synthesis and Purification of PhLi

Iodobenzene (10.7 mL, 96 mmol) was dissolved in hexane (200 mL) and cooled to -78 °C. $^{n}$BuLi (1.6 M, 60 mL, 96 mmol) was added dropwise and the reaction was maintained at -78 °C for 30 minutes then warmed to room temperature and stirred for 2 hours giving a thick colourless suspension. Additional hexane (2 × 100 mL) was added to loosen the suspension, and the solids were collected on a filter frit, then washed with hexane (2 × 50 mL), and dried *in vacuo*. Yield – 7.85 g (97%).

2.52 g of PhLi was suspended in hexane (40 mL) and $Et_2O$ (5 mL) was added. The cloudy solution was filtered to remove insoluble impurities and the filtrate was stored at -40 °C for 3 hours to give colourless crystals of $[PhLi(Et_2O)]_4$. The supernatant was removed *via* filter cannula and the crystals were thoroughly dried *in vacuo* for several hours to remove coordinated $Et_2O$. Yield – 2.15 g (84% recovery). $^{1}$H NMR spectroscopy indicates 2% residual $Et_2O$ (MW = 85.52 g mol$^{-1}$).

**$^{1}$H NMR** (300.1 MHz, THF-$d_8$): δ 7.92 (m, 2H, *o*-C*H*), 6.85 (m, 2H, *m*-C*H*), 6.75 (m, 1H, *p*-C*H*).

$^{7}$**Li NMR** (116.6 MHz, THF-$d_8$): δ 1.56 (s).

Analytical data in accordance with the literature.[4]

**Synthesis of Alkali-Metal Nickelates**

**Synthesis of [(*ttt*-CDT)NiPh][Li(12-crown-4)$_2$] (1)**

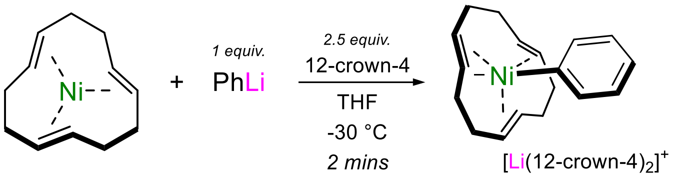

**1**

Ni(*ttt*-CDT) (22.1 mg, 0.1 mmol) was dissolved in pre-cooled THF (0.5 mL) and cooled to -30 °C. PhLi (8.6 mg, 0.1 mmol) was slowly added as a pre-cooled solution in THF (0.5 mL) resulting in a colour change from red to yellow. After stirring for 1 minute at -30 °C, 12-crown-4 (40 µL, 0.25 mmol) was added and the solution was stirred for an additional minute before being filtered and stored at -30 °C for crystallisation *via* pentane vapour diffusion. After 48 hours, the supernatant was decanted from the yellow crystals, which were washed with cold pentane (2 × 0.5 mL) and dried under argon. Yield – 39 mg (59%).

$^{1}$**H NMR** (400.1 MHz, THF-$d_8$, -20 °C): δ 7.72 (br, 2H, Ph-*o*-C*H*), 6.41 (br, 2H, Ph-*m*-C*H*), 6.20 (br, 1H, Ph-*p*-C*H*), 3.39(s, 32H, 12-crown-4-C*H₂*), 3.23 (br, 6H, CDT-C*H₂*), 1.74 (br, 6H, CDT-C*H₂*), 1.40 (br, 3H, CDT-C*H*), 1.00 (br, 3H, C*H*).

$^{7}$**Li NMR** (155.5 MHz, THF-$d_8$, -20 °C): δ -0.69 (s).

$^{13}$**C{$^{1}$H} NMR** (100.6 MHz, THF-$d_8$, -20 °C): δ 195.3 (Ph-*ipso*-C), 144.4 (Ph-*o*-CH), 124.6 (Ph-*m*-CH), 117.5 (Ph-*p*-CH), 87.2 (CDT-CH), 82.4 (CDT-CH), 69.2 (12-crown-4-$CH_2$), 40.5 (CDT-$CH_2$), 38.7 (CDT-$CH_2$).

**Elemental Analysis:** Calculated for $C_{34}H_{55}LiNiO_8$: C, 62.12; H, 8.43. Found: C, 62.16; H, 8.43.

**Synthesis of $Li_2$(TMEDA)$_2$$Ph_2$Ni($\eta^2$-CDT) (2)**

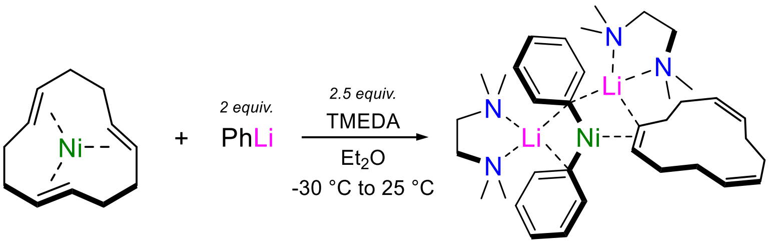

**2**

Ni(*ttt*-CDT) (44.2 mg, 0.2 mmol) was dissolved in $Et_2O$ (0.5 mL) and cooled to -30 °C. PhLi (34.2 mg, 0.4 mmol) was slowly added as a chilled solution in $Et_2O$ (0.5 mL) followed by TMEDA (75 µL, 0.5 mmol). The orange/red solution was warmed to room temperature and stirred for 15 minutes, then filtered and stored at -30 °C for crystallisation. After 4 hours, the orange/brown solids were decanted from the supernatant, washed with cold pentane (2 × 0.5 mL) and dried under argon. Yield – 20 mg (16%).

$^{1}$**H NMR** (300.1 MHz, THF-$d_8$): δ 7.91 (d, $J$ = 6.7 Hz, 4H, Ph-*o*-C*H*), 6.71 (t, $J$ = 7.1 Hz, 4H, Ph-*m*-C*H*), 6.49 (t, $J$ = 6.9 Hz, 2H, Ph-*p*-C*H*), 5.35–5.19 (m, 2H, CDT-C*H*), 5.12–4.96 (m, 2H, CDT-C*H*), 2.30 (s, 8H,

TMEDA-C$\underline{H_2}$), 2.14 (s, 24H, TMEDA-C$\underline{H_3}$), 2.14–1.98 (m, 4H + 2H, CDT-C$\underline{H_2}$), 1.96–1.74 (m, 4H, CDT-C$\underline{H_2}$), 1.06–0.90 (m, 2H, CDT-C$\underline{H_2}$), 0.16 (d, $J$ = 6.6 Hz, 2H, CDT-C$\underline{H}$-coordinated).

**$^{7}$Li NMR** (116.6 MHz, THF-d$_8$): δ 0.38 (s).

**$^{13}$C{$^{1}$H} NMR** (75.5 MHz, THF-d$_8$): δ 191.9 (Ph-*ipso*-$\underline{C}$), 142.5 (Ph-*o*-$\underline{C}$H), 137.0 (CDT-$\underline{C}$H), 127.3 (CDT-$\underline{C}$H), 125.9 (Ph-*m*-$\underline{C}$H), 119.2 (Ph-*p*-$\underline{C}$H), 59.1 (TMEDA-$\underline{C}$H$_2$), 46.8 (CDT-$\underline{C}$H-coordinated), 46.4 (TMEDA-$\underline{C}$H$_3$), 37.3 (CDT-$\underline{C}$H$_2$), 36.8 (CDT-$\underline{C}$H$_2$), 32.6 (CDT-$\underline{C}$H$_2$).

**Elemental Analysis:** Calculated for $C_{36}H_{60}Li_2N_4Ni$: C, 69.58; H, 9.73; N, 9.02. Found: C, 68.97; H, 9.91; N, 8.87.

**Synthesis of $Li_4(TMEDA)_2(Et_2O)_2Ph_4Ni_2(\mu;\eta^2;\eta^2\text{-}C_6H_4)$ (3)**

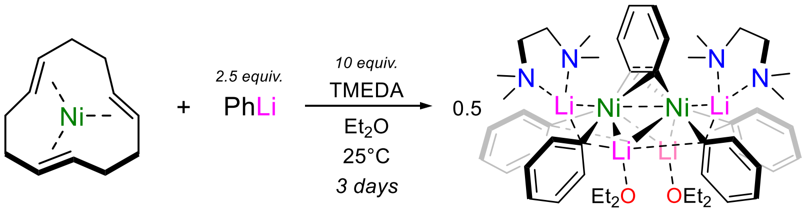

**3**

Ni(*ttt*-CDT) (44.2 mg, 0.2 mmol) was dissolved in Et$_2$O (1 mL) and PhLi (43 mg, 0.5 mmol) was added followed by TMEDA (300 µL, 2.0 mmol). The reaction was stirred at room temperature for 3 days giving a deep red solution which was filtered and stored at -30 °C for crystallisation *via* pentane vapour diffusion. After 1 week, the supernatant was decanted from the dark red crystals, which were washed with cold pentane (2 × 0.5 mL) and dried under argon. Yield – 30 mg (33%).

**$^{1}$H NMR** (300.1 MHz, THF-d$_8$): δ 8.12 (d, $J$ = 6.0 Hz, 8H, Ph-*o*-C$\underline{H}$), 6.71–6.63 (m, 10H, Ph-*m*-C$\underline{H}$ + $\underline{H}$3), 6.48 (tt, $J$ = 7.2, 1.6 Hz, 4H, Ph-*p*-C$\underline{H}$), 5.77 (dd, $J$ = 5.5, 2.5 Hz, 2H, $\underline{H}$2), 3.39 (q, Et$_2$O-C$\underline{H_2}$), 2.31 (s, 8H, TMEDA-C$\underline{H_2}$), 2.15 (s, 24H, TMEDA-C$\underline{H_3}$), 1.12 (t, Et$_2$O-C$\underline{H_3}$).

**$^{7}$Li NMR** (116.1 MHz, THF-d$_8$): δ 0.50 (vbr), -0.24 (vbr).

**$^{13}$C{$^{1}$H} NMR** (75.5 MHz, THF-d$_8$): δ 185.6 (Ph-*ipso*-$\underline{C}$), 144.1 (Ph-*o*-$\underline{C}$H), 126.0 (Ph-*m*-$\underline{C}$), 125.2 ($\underline{C}$3), 120.6 (Ph-*p*-$\underline{C}$H), 115.9 ($\underline{C}$2), 71.8 (br, $\underline{C}$1), 66.5 (Et$_2$O-$\underline{C}$H$_2$), 59.1 (TMEDA-$\underline{C}$H$_2$), 46.4 (TMEDA-$\underline{C}$H$_3$), 15.8 (Et$_2$O-$\underline{C}$H$_3$).

**Elemental Analysis:** Calculated for $C_{50}H_{76}Li_4N_4Ni_2O_2$: C, 65.97; H, 8.42; N, 6.15. Found: C, 65.65; H, 8.26; N, 6.04.

**Synthesis of $Li_2(THF)_4Ph_2Ni(\eta^2\text{-anthracene})$ (4Li)**

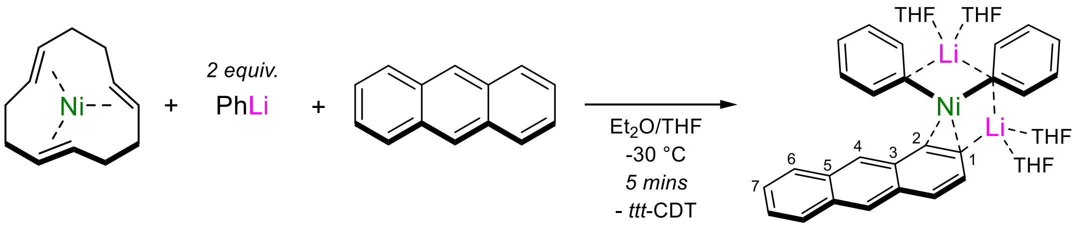

**4Li**

Ni(*ttt*-CDT) (22.1 mg, 0.1 mmol) was dissolved in $Et_2O$ (1 mL) and cooled to -30 °C. PhLi (17.1 mg, 0.2 mmol) was added and the solution was warmed to room temperature, stirred for 2-3 minutes, then recooled to -30 °C. Anthracene (17.8 mg, 0.1 mmol) was added followed by THF (0.1 mL) and the dark red reaction mixture was stirred at -30 °C for 5 minutes, then filtered and stored at -30 °C for crystallisation *via* pentane vapour diffusion. After 72 hours, the supernatant was decanted from the dark red/black crystals, which were washed with cold pentane (2 × 0.5 mL) and dried under argon. Yield – 36 mg (52%).

**$^1$H NMR** (300.1 MHz, THF-$d_8$): δ 8.00 (d, *J* = 6.7 Hz, 2H, Ph-*o*-C<u>*H*</u>), 7.44 (d, *J* = 6.7 Hz, 2H, Ph-*o*-C<u>*H*</u>), 7.00 (m, 2H, <u>*H*</u>6), 6.69–6.59 (m, 4H, Ph-*m*-C<u>*H*</u> + <u>*H*</u>7), 6.59–6.51 (m, 2H, Ph-*m*-C<u>*H*</u>), 6.49–6.37 (m, 4H, Ph-*p*-C<u>*H*</u> + <u>*H*</u>4), 4.44 (br, 4H, <u>*H*</u>1/2), 3.62 (THF), 1.78 (THF).

**$^7$Li NMR** (116.6 MHz, THF-$d_8$): δ 0.26 (br).

**$^{13}$C{$^1$H} NMR** (75.5 MHz, THF-$d_8$): δ 189.8 (*ipso*-<u>*C*</u>), 184.5 (*ipso*-<u>*C*</u>), 143.1 (<u>*C*</u>3), 142.1 (*o*-<u>*C*</u>H), 140.7 (*o*-<u>*C*</u>H), 134.6 (<u>*C*</u>5), 125.1 (*m*-<u>*C*</u>H), 124.3 (*m*-<u>*C*</u>H), 124.1 (<u>*C*</u>6), 120.3 (<u>*C*</u>7), 119.4 (*p*-<u>*C*</u>H), 119.3 (*p*-<u>*C*</u>H), 113.5 (<u>*C*</u>4), 90.3 (<u>*C*</u>2), 73.8 (<u>*C*</u>1).

**Elemental Analysis:** Calculated for $C_{42}H_{52}Li_2NiO_4$: C, 72.75; H, 7.56. Found: C, 72.68; H, 7.73.

**Synthesis of $Na_2(TMEDA)_2Ph_2Ni(\eta^2$-anthracene) (4Na)**

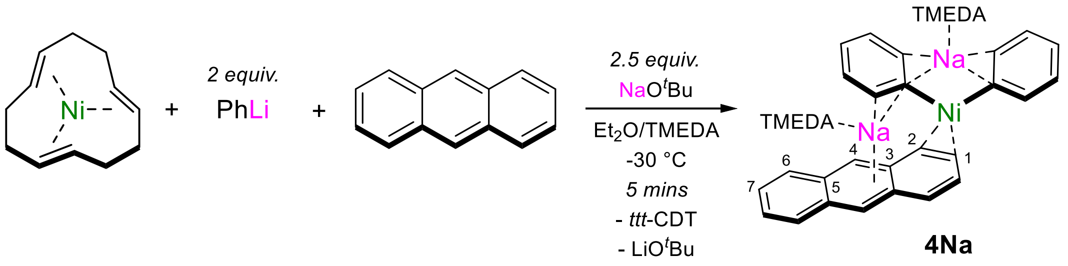

**4Na**

Ni(*ttt*-CDT) (22.1 mg, 0.1 mmol) was dissolved in $Et_2O$ (1 mL) and cooled to -30 °C. PhLi (17.1 mg, 0.2 mmol) was added and the solution was warmed to room temperature, stirred for 2-3 minutes, then recooled to -30 °C. Anthracene (17.8 mg, 0.1 mmol) was added followed by toluene (1 mL), NaO$^t$Bu (24 mg, 0.25 mmol) and TMEDA (90 µL, 0.6 mmol) and the dark green reaction mixture was stirred at -30 °C for 5 minutes, then filtered and stored at -30 °C for crystallisation *via* pentane vapour diffusion. After 72 hours, the supernatant was decanted from the dark green/black crystals, which were washed with cold pentane (2 × 0.5 mL) and dried under argon. Yield – 28 mg (42%).

**$^1$H NMR** (300.1 MHz, THF-$d_8$): δ 7.60 (m, 4H, Ph-*o*-C<u>*H*</u>), (m, 4H, Ph-*m*-C<u>*H*</u>), 6.87 (dd, *J* = 3.3, 2.6 Hz, 2H, <u>*H*</u>6), 6.70–6.62 (m, 4H, Ph-*m*-C<u>*H*</u>), 6.60–6.50 (m, 4H, Ph-*p*-C<u>*H*</u> + <u>*H*</u>7), 6.35 (s, 2H, <u>*H*</u>4), 4.58 (dd, *J* = 2.8, 2.3 Hz, 2H, <u>*H*</u>2), 4.42 (dd, *J* = 2.8, 2.3 Hz, 2H, <u>*H*</u>1), 2.30 (s, 8H, TMEDA-C<u>$H_2$</u>), 2.15 (s, 24H, TMEDA-C<u>$H_3$</u>).

**$^{13}$C{$^1$H} NMR** (75.5 MHz, THF-$d_8$): δ 182.3 (Ph-*ipso*-<u>*C*</u>), 175.4 (Ph-*ipso*-<u>*C*</u>), 143.78 (<u>*C*</u>3), 142.8 (Ph-*o*-<u>*C*</u>H), 142.2 (Ph-*o*-<u>*C*</u>H), 134.6 (<u>*C*</u>5), 126.3 (Ph-*m*-<u>*C*</u>H), 126.0 (Ph-*m*-<u>*C*</u>H), 123.9 (<u>*C*</u>6), 120.8 (<u>*C*</u>7), 119.9 (Ph-*p*-<u>*C*</u>H), 109.7 (<u>*C*</u>4), 86.5 (<u>*C*</u>2), 72.2 (<u>*C*</u>1), 59.1 (TMEDA-<u>*C*</u>$H_2$), 46.4 (TMEDA-<u>*C*</u>$H_3$).

**Elemental Analysis:** Calculated for $C_{38}H_{52}N_4Na_2Ni$: C, 68.17; H, 7.83; N, 8.37. Found: C, 66.6; H, 7.67; N, 7.66.

*N.B.* Crystalline samples were consistently low in carbon, hydrogen and nitrogen suggesting partial TMEDA decoordination.

**Synthesis of $K_2$(PMDETA)$_2$Ph$_2$Ni($\eta^2$-anthracene) (4K)**

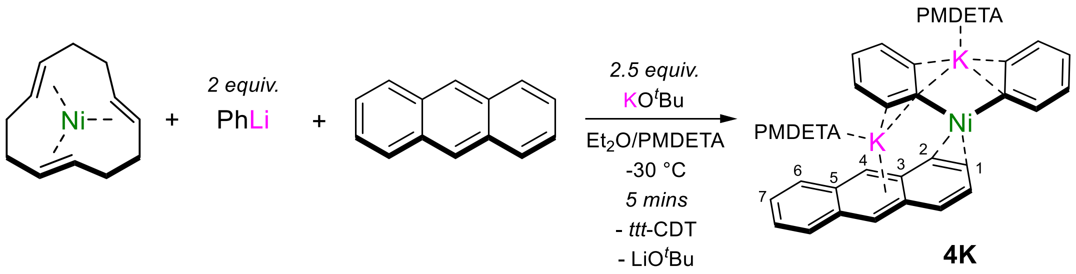

**4K**

Ni(*ttt*-CDT) (22.1 mg, 0.1 mmol) was dissolved in $Et_2O$ (1 mL) and cooled to -30 °C. PhLi (17.1 mg, 0.2 mmol) was added and the solution was warmed to room temperature, stirred for 2-3 minutes, then recooled to -30 °C. Anthracene (17.8 mg, 0.1 mmol) was added followed by toluene (1 mL), KO$^t$Bu (28 mg, 0.25 mmol) and PMDETA (125 µL, 0.6 mmol) and the dark green reaction mixture was stirred at -30 °C for 5 minutes, then filtered and stored at -30 °C for crystallisation *via* pentane vapour diffusion. After 72 hours, the supernatant was decanted from the dark green/black crystals, which were washed with cold pentane (2 × 0.5 mL) and dried under argon. Yield – 22 mg (24%).

**$^1$H NMR** (300.1 MHz, THF-d$_8$): δ 7.69 (m, 4H, Ph-*o*-C<u>*H*</u>), 6.72 (dd, *J* = 3.6, 2.1 Hz, 2H, <u>*H*</u>6), 6.69–6.60 (m, 4H, Ph-*m*-C<u>*H*</u>), 6.51–6.44 (m, 2H, Ph-*p*-C<u>*H*</u>), 6.48 (dd, *J* = 3.6, 2.1 Hz, 2H, <u>*H*</u>7), 6.17 (s, 2H, <u>*H*</u>4), 4.50 (m, <u>*H*</u>2), 4.24 (m, 2H, <u>*H*</u>1), 2.41 (m, 8H, PMDETA-C<u>*H*$_2$</u>), 2.31 (m, 8H, PMDETA-C<u>*H*$_2$</u>), 2.19 (s, 6H, PMDETA-C<u>*H*$_3$</u>), 2.15 (s, 24H, PMDETA-C<u>*H*$_3$</u>).

**$^{13}$C{$^1$H} NMR** (75.5 MHz, THF-d$_8$): δ 144.7 (<u>*C*</u>3), 142.5 (Ph-*o*-<u>C</u>H), 141.9 (Ph-*o*-<u>C</u>H), 134.8 (<u>*C*</u>5), 126.0 (Ph-*m*-<u>C</u>H), 125.5 (Ph-*m*-<u>C</u>H), 123.0 (<u>*C*</u>6), 120.3 (<u>*C*</u>7), 119.6 (Ph-*p*-<u>C</u>H), 119.0 (Ph-*p*-<u>C</u>H), 106.8 (<u>*C*</u>4), 87.1 (<u>*C*</u>2), 73.1 (<u>*C*</u>1), 59.0 (PMDETA-<u>C</u>H$_2$), 57.5 (PMDETA-<u>C</u>H$_2$), 46.5 (PMDETA-<u>C</u>H$_3$), 43.5 (PMDETA-<u>C</u>H$_3$).

*N.B.* Due to poor solubility and limited stability at room temperature, signals for the Ph-*ipso*-carbons could not be observed in the $^{13}$C{$^1$H} or $^1$H-$^{13}$C HMBC spectra.

**Elemental Analysis:** Calculated for $C_{44}H_{66}K_2N_6Ni$: C, 64.77; H, 8.15; N, 10.30. Found: C, 64.08; H, 8.01; N, 9.78.

*N.B.* Crystalline samples were consistently low in carbon, hydrogen and nitrogen suggesting partial PMDETA decoordination.

**Synthesis of $Li_2$(THF)$_4$Ph$_2$Ni($\eta^2$-phenanthrene) (5Li)**

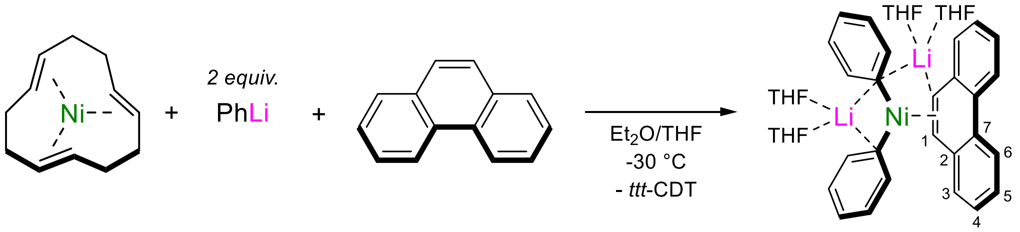

**5Li**

Ni(*ttt*-CDT) (22.1 mg, 0.1 mmol) was dissolved in $Et_2O$ (1 mL) and cooled to -30 °C. PhLi (17.1 mg, 0.2 mmol) was added and the solution was warmed to room temperature, stirred for 2-3 minutes, then recooled to -30 °C. Phenanthrene (17.8 mg, 0.1 mmol) was added followed by THF (0.1 mL) and the dark red reaction mixture was stirred at -30 °C for 5 minutes, then filtered and stored at -30 °C for crystallisation *via* pentane vapour diffusion. After 72 hours, the supernatant was decanted from the dark red/brown crystals, which were washed with cold pentane (2 × 0.5 mL) and dried under argon. Yield – 38 mg (55%).

**$^1$H NMR** (300.1 MHz, THF-$d_8$): δ 7.50 (d, *J* = 6.5 Hz, 4H, Ph-*o*-C<u>*H*</u>), 7.38 (d, *J* = 7.6 Hz, 2H, <u>*H*</u>6), 6.64–6.56 (m, 6H, Ph-*m*-C<u>*H*</u> + <u>*H*</u>5), 6.48–6.39 (m, 4H, Ph-*p*-C<u>*H*</u> + <u>*H*</u>4), 6.27 (d, *J* = 7.0 Hz, 2H, <u>*H*</u>3), 3.62 (THF), 2.78 (s, 2H, <u>*H*</u>1), 1.78 (THF).

**$^7$Li NMR** (116.6 MHz, THF-$d_8$): δ -0.27 (s).

**$^{13}$C{$^1$H} NMR** (75.5 MHz, THF-$d_8$): δ 189.7 (Ph-*ipso*-<u>*C*</u>), 145.7 (<u>*C*</u>7), 142.0 (Ph-*o*-<u>*C*</u>H), 129.6 (<u>*C*</u>2), 126.8 (<u>*C*</u>3), 126.0 (<u>*C*</u>5), 124.6 (Ph-*m*-<u>*C*</u>H), 121.0 (<u>*C*</u>6), 119.2 (Ph-*p*-<u>*C*</u>H), 116.3 (<u>*C*</u>4), 39.0 (<u>*C*</u>1).

**Elemental Analysis:** Calculated for $C_{42}H_{52}Li_2NiO_4$: C, 72.75; H, 7.56. Found: C, 71.69; H, 7.57. Spectroscopically pure and crystalline samples were consistently low in carbon.

**Synthesis of [$Na_2(THF)_3Ph_2Ni(\eta^2$-phenanthrene)]$_2$ (5Na)**

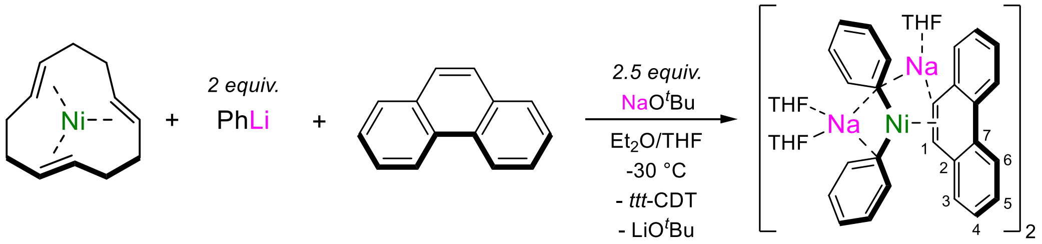

**5Na**

Ni(*ttt*-CDT) (22.1 mg, 0.1 mmol) was dissolved in $Et_2O$ (1 mL) and cooled to -30 °C. PhLi (17.1 mg, 0.2 mmol) was added and the solution was warmed to room temperature, stirred for 2-3 minutes, then recooled to -30 °C. Phenanthrene (17.8 mg, 0.1 mmol) was added followed by THF (0.1 mL) and NaO$^t$Bu (24 mg, 0.25 mmol) and the dark red reaction mixture was stirred at -30 °C for 5 minutes, then filtered and stored at -30 °C for crystallisation *via* pentane vapour diffusion. After 48 hours, the supernatant was decanted from the dark red/black crystals, which were washed with cold pentane (2 × 0.5 mL) and dried under argon. Yield – 41 mg (63%).

**$^1$H NMR** (300.1 MHz, THF-$d_8$): δ 7.53 (d, *J* = 6.7 Hz, 4H, Ph-*o*-C<u>*H*</u>), 7.37 (d, *J* = 7.8 Hz, 2H, <u>*H*</u>6), 6.69-6.56 (m, 8H, Ph-*m*-C<u>*H*</u> + <u>*H*</u>3 + <u>*H*</u>5), 6.53-6.41 (m, 4H, Ph-*p*-C<u>*H*</u> + H5), 3.62 (THF), 3.05 (s, 2H, <u>*H*</u>1), 1.78 (THF).

**$^{13}$C{$^1$H} NMR** (75.5 MHz, THF-$d_8$): δ 187.8 (Ph-*ipso*-C), 146.5 (<u>*C*</u>7), 142.4 (Ph-*o*-<u>*C*</u>H), 129.0 (<u>*C*</u>2), 126.9 (<u>*C*</u>5), 125.5 (Ph-*m*-<u>*C*</u>H), 125.1 (<u>*C*</u>3), 121.5 (<u>*C*</u>6), 119.5 (Ph-*p*-CH), 116.7 (<u>*C*</u>4), 38.9 (<u>*C*</u>1).

**Elemental Analysis:** Calculated for $C_{38}H_{44}Na_2NiO_3$: C, 69.85; H, 6.79. Found: C, 69.30; H, 6.82.

**Synthesis of [$K_2(THF)_1Ph_2Ni(\eta^2$-phenanthrene)]$_\infty$ (5K)**

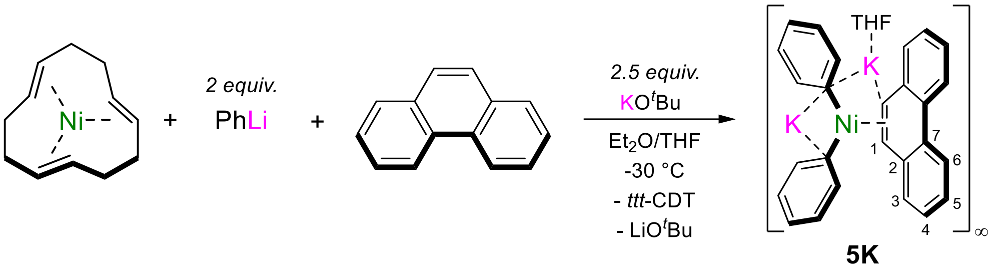


Ni(*ttt*-CDT) (22.1 mg, 0.1 mmol) was dissolved in $Et_2O$ (1 mL) and cooled to -30 °C. PhLi (17.1 mg, 0.2 mmol) was added and the solution was warmed to room temperature, stirred for 2-3 minutes, then recooled to -30 °C. Phenanthrene (17.8 mg, 0.1 mmol) was added followed by THF (0.1 mL) and KO$^t$Bu (28 mg, 0.25 mmol) and the dark red reaction mixture was stirred at -30 °C for 5 minutes, then filtered and stored at -30 °C for crystallisation *via* pentane vapour diffusion. After 72 hours, the supernatant was decanted from the dark purple/black crystals, which were washed with cold pentane (2 × 0.5 mL) and dried under argon. Yield – 23 mg (43%).

**$^1$H NMR** (300.1 MHz, THF-$d_8$): δ 7.64 (dd, *J* = 7.5, 1.4 Hz, 4H, Ph-*ortho*-C<u>H</u>), 7.27 (d, *J* = 7.2 Hz, 2H, <u>H</u>6), 6.62 (m, 6H, Ph-*meta*-C<u>H</u> + <u>H</u>3), 6.49 (td, *J* = 7.9, 1.2 Hz, 2H, <u>H</u>5), 6.43 (tt, *J* = 7.2, 1.5 Hz, 2H, Ph-*para*-C<u>H</u>), 6.34 (td, *J* = 6.8, 1.4 Hz, 2H, <u>H</u>4), 3.62 (THF), 3.09 (s, 2H, <u>H</u>1), 1.78 (THF).

**$^{13}$C{$^1$H} NMR** (75.5 MHz, THF-$d_8$): δ 193.7 (Ph-*ipso*-<u>C</u>), 147.6 (<u>C</u>7), 142.7 (Ph-*ortho*-<u>C</u>H), 128.2 (<u>C</u>2), 127.1 (<u>C</u>5), 125.4 (Ph-*meta*-<u>C</u>H), 124.2 (<u>C</u>3), 121.5 (<u>C</u>6), 118.5 (Ph-*para*-<u>C</u>H), 115.8 (<u>C</u>4), 40.5 (<u>C</u>1).

**Elemental Analysis:** Calculated for $C_{30}H_{28}K_2NiO$: C, 66.55; H, 5.21. Found: C, 66.25; H, 5.21.

**Synthesis of [$Li(THF)_2Ph_2Ni(\eta^3$-perylene)][$Li(THF)_4$] (6Li)**

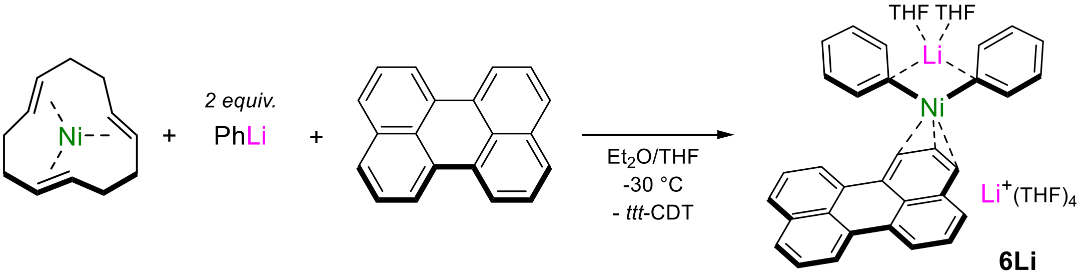


Ni(*ttt*-CDT) (22.1 mg, 0.1 mmol) was dissolved in $Et_2O$ (1 mL) and cooled to -30 °C. PhLi (17.1 mg, 0.2 mmol) was added and the solution was warmed to room temperature, stirred for 2-3 minutes, then recooled to -30 °C. Perylene (25.2 mg, 0.1 mmol) was added followed by THF (0.5 mL) and the dark purple reaction mixture was stirred at -30 °C for 5 minutes, then filtered and stored at -30 °C for crystallisation *via* pentane vapour diffusion. After 72 hours, the supernatant was decanted from the dark blue/purple crystals, which were washed with cold pentane (2 × 0.5 mL) and dried under argon. Yield – 65 mg (71%).

*N.B.* Isolated samples were contaminated with small bronze crystals identified as the perylene radical anion by EPR spectroscopy (see **Figure S5**) and could therefore not be isolated in pure form for suitable elemental analysis.

$^{1}$**H NMR** (300.1 MHz, THF-d$_8$): δ 7.89 (d, *J* = 6.9 Hz, 2H), 7.54 (d, *J* = 6.8 Hz, 2H), 6.80–6.65 (m, 6H), 6.59–6.47 (m, 4H), 6.23 (t, *J* = 7.4 Hz, 2H), 6.08 (d, *J* = 7.2 Hz, 2H), 5.84 (br, 2H), 5.65 (br, 2H), 4.60 (vbr, 2H), 3.62 (THF), 1.78 (THF).

$^{7}$**Li NMR** (116.6 MHz, THF-d$_8$): δ -0.68 (s).

$^{13}$**C{$^{1}$H} NMR** (75.5 MHz, THF-d$_8$): δ 178.5, 176.2, 143.0, 139.7, 129.1, 125.3, 125.2, 121.2, 114.6. Signals for the coordinated perylene could not be observed in the $^{13}$C{$^{1}$H}, $^{1}$H-$^{13}$C HSQC or $^{1}$H-$^{13}$C HMBC spectra.

**Synthesis of [Li(THF)$_2$Ph$_2$Ni($\eta^2$-coronene)][Li(THF)$_4$] (7Li)**

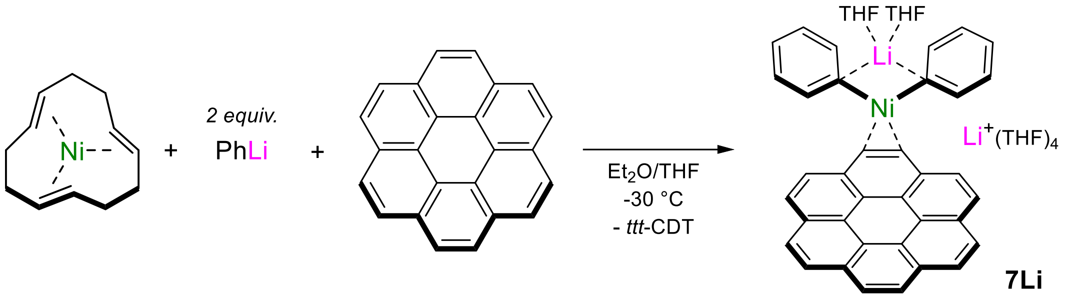

**7Li**

Ni(*ttt*-CDT) (22.1 mg, 0.1 mmol) was dissolved in Et$_2$O (1 mL) and cooled to -30 °C. PhLi (17.1 mg, 0.2 mmol) was added and the solution was warmed to room temperature, stirred for 2-3 minutes, then recooled to -30 °C. Coronene (30.0 mg, 0.1 mmol) was added followed by THF (1 mL) and the dark reaction mixture was stirred at -30 °C for 5 minutes, then filtered and stored at -30 °C for crystallisation *via* pentane vapour diffusion. After 48 hours, the supernatant was decanted from the dark black/green crystals, which were washed with cold pentane (2 × 0.5 mL) and dried under argon. Yield – 24 mg (24%).

$^{1}$**H NMR** (400.1 MHz, THF-d$_8$, -20 °C): δ 7.45 (s, 2H), 7.39 (d, *J* = 6.5 Hz, 4H, Ph-*o*-C<u>*H*</u>), 7.10 (s, 4H), 6.98 (d, *J* = 7.9 Hz, 2H), 6.59 (t, J = 7.0 Hz, 4H, Ph-*m*-C<u>*H*</u>), 6.41–6.32 (m, 2H + 2H, Ph-*p*-C<u>*H*</u>), 3.62 (THF), 3.30 (s, 2H), 1.78 (THF).

$^{7}$**Li NMR** (155.5 MHz, THF-d$_8$, -40 °C): δ 0.52 (br), 0.00 (br).

$^{13}$**C{$^{1}$H} NMR** (100.6 MHz, THF-d$_8$, -20 °C): δ 189.6 (Ph-*ipso*-<u>C</u>), 142.0, 140.6 (Ph-*o*-<u>C</u>H), 130.6, 128.7, 128.6, 128.4, 125.1, 124.5, 124.4, 124.2 (Ph-*m*-<u>C</u>H), 123.3, 119.7, 119.0 (Ph-*p*-<u>C</u>H), 42.3.

**Elemental Analysis:** Calculated for $C_{60}H_{70}Li_2NiO_6$: C, 75.09; H, 7.35. Found: C, 74.91; H, 7.35.

**Synthesis of K$_2$(DME)$_4$Ph$_2$Ni($\eta^2$-coronene) (7K)**

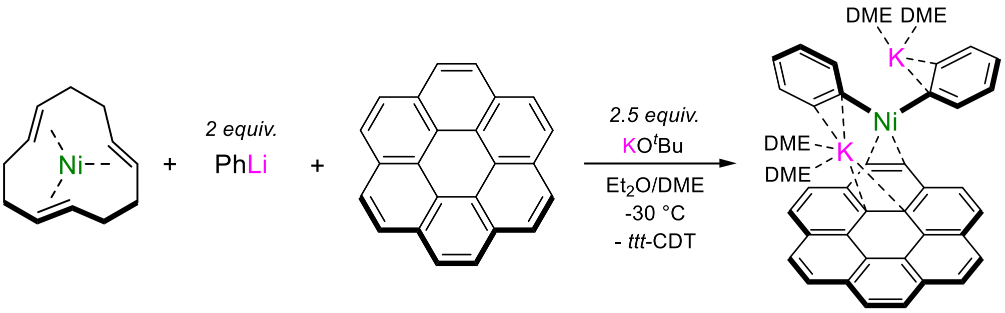

**7K**

Ni(*ttt*-CDT) (11.0 mg, 0.05 mmol) was dissolved in Et$_2$O (1 mL) and cooled to -30 °C. PhLi (8.6 mg, 0.1 mmol) was added and the solution was warmed to room temperature, stirred for 2-3 minutes, then recooled

to -30 °C. Coronene (15.0 mg, 0.05 mmol) was added slowly as a solution/suspension in DME (1 mL), followed by KO$^t$Bu (14 mg, 0.125 mmol) and the black reaction mixture was stirred at -30 °C for 5 minutes, then filtered and stored at -30 °C for crystallisation *via* pentane vapour diffusion. After 72 hours, the supernatant was decanted from the dark black crystals, which were washed with cold pentane (2 × 0.5 mL) and dried under argon. Yield – 21 mg (44%).

*N.B.* Isolated samples were found to be contaminated with the coronene radical anion by EPR spectroscopy (see **Figure S8**) and could therefore not be isolated in pure form for suitable elemental analysis.

**$^1$H NMR** (300.1 MHz, THF-$d_8$): δ 7.62 (d, *J* = 6.7 Hz, 4H, Ph-*o*-C<u>*H*</u>), 7.18 (vbr, coronene), 6.62 (t, *J* = 6.9 Hz, 4H, Ph-*m*-C<u>*H*</u>), 6.41 (t, *J* = 6.9 Hz, 2H, Ph-*p*-C<u>*H*</u>), 3.53 (DME-C<u>$H_2$</u>), 3.35 (DME-C<u>$H_3$</u>).

*N.B.* Due to poor solubility of **7K**, low temperature measurements were not possible.

**Synthesis of [$Li_2(THF)_3Ph_2Ni(\eta^2$-$Ph_2CO)]_2$ (8Li)**

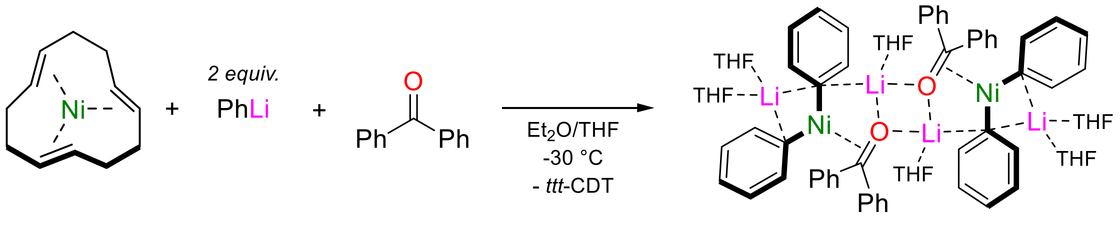


**8Li**

Ni(*ttt*-CDT) (22.1 mg, 0.1 mmol) was dissolved in $Et_2O$ (1 mL) and cooled to -30 °C. PhLi (17.1 mg, 0.2 mmol) was added and the solution was warmed to room temperature, stirred for 2-3 minutes, then recooled to -30 °C. Benzophenone (18.2 mg, 0.1 mmol) was added followed by THF (0.1 mL) and the dark purple/green reaction mixture was stirred at -30 °C for 5 minutes, then filtered and stored at -30 °C for crystallisation *via* pentane vapour diffusion. After 72 hours, the supernatant was decanted from the dark purple crystals, which were washed with cold pentane (2 × 0.5 mL) and dried under argon. Yield – 39 mg (62%).

*N.B.* The $^1$H NMR spectrum of **8Li** was very broad at room temperature and whilst cooling to -20 °C does lead to improvement of resolution and sharpening of signals, the significant overlap means it is not possible to confidently assign signals (see **Spectra 33–35**).

**Elemental Analysis:** Calculated for $C_{37}H_{44}Li_2NiO_4$: C, 71.07; H, 7.09. Found: C, 70.58; H, 7.14.

**Synthesis of [$K_2(DME)_3Ph_2Ni(\eta^2$-$Ph_2CO)]_2$ (8K)**

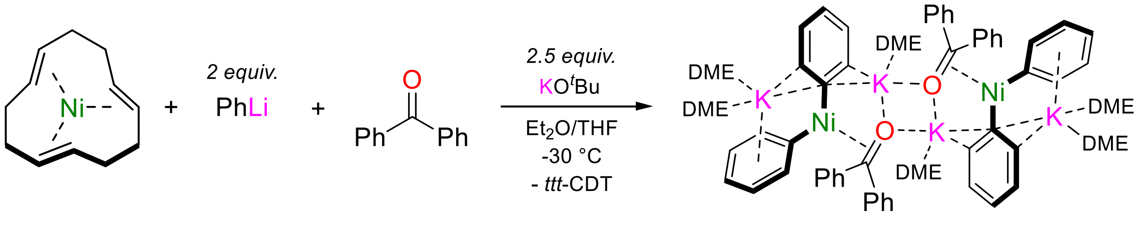


**8K**

Ni(*ttt*-CDT) (22.1 mg, 0.1 mmol) was dissolved in $Et_2O$ (1 mL) and cooled to -30 °C. PhLi (17.1 mg, 0.2 mmol) was added and the solution was warmed to room temperature, stirred for 2-3 minutes, then recooled to -30 °C. Benzophenone (18.2 mg, 0.1 mmol) was added followed by DME (0.5 mL) and KO$^t$Bu (28 mg, 0.25 mmol) and the black reaction mixture was stirred at -30 °C for 5 minutes, then filtered and stored at -30 °C for crystallisation *via* pentane vapour diffusion. After 72 hours, the supernatant was decanted from the dark blue crystals, which were washed with cold pentane (2 × 0.5 mL) and dried under argon. Yield – 37 mg (50%).

**$^1$H NMR** (300.1 MHz, THF-$d_8$): δ 7.99 (d, *J* = 5.9 Hz, 2H, Ph-*o*-C*H*), 7.40 (d, *J* = 7.0 Hz, 4H, $Ph_2$CO-*o*-C*H*), 7.28 (d, *J* = 7.0 Hz, 2H, Ph-*o*-C*H*), 6.90 (t, *J* = 6.9 Hz, 4H, $Ph_2$CO-*m*-C*H*), 6.77 (t, *J* = 6.3 Hz, 2H, $Ph_2$CO-*p*-C*H*), 6.64 (d, *J* = 6.9 Hz, 2H, Ph-*m*-C*H*), 6.59 (d, *J* = 6.7 Hz, 1H, Ph-*p*-C*H*), 6.41 (t, *J* = 7.0 Hz, 2H, Ph-*m*-C*H*), 6.29 (t, *J* = 7.0 Hz, 1H, Ph-*o*-C*H*), 3.43 (DME-C$H_2$), 3.27 (DME-C$H_3$).

**$^{13}$C{$^1$H} NMR** (75.5 MHz, THF-$d_8$): δ 185.4 (Ph-*ipso*-C), 153.6 ($Ph_2$CO-*ipso*-C), 143.1 (br, Ph-*o*-CH), 141.2 (Ph-*o*-CH), 127.9 ($Ph_2$CO-*m*-CH), 125.9 ($Ph_2$CO-*p*-CH), 124.8 (br, $Ph_2$CO-*o*-CH), 124.5 (Ph-*m*-CH), 120.0 Ph-*p*-CH), 118.7 (Ph-*m*-CH), 117.7 (Ph-*p*-CH), 72.9 (DME-$CH_2$), 59.1 (DME-$CH_3$).

*N.B.* Signals for the carbonyl carbon not observed in the $^{13}$C{$^1$H} or $^1$H-$^{13}$C HMBC spectra.

**Elemental Analysis:** Calculated for $C_{37}H_{50}K_2NiO_7$: C, 59.76; H, 6.78. Found: C, 59.72; H, 6.60.

**Synthesis of $Li_2(THF)_5Ph_2Ni(\eta^2$-PhCH=NPh) (9Li)**

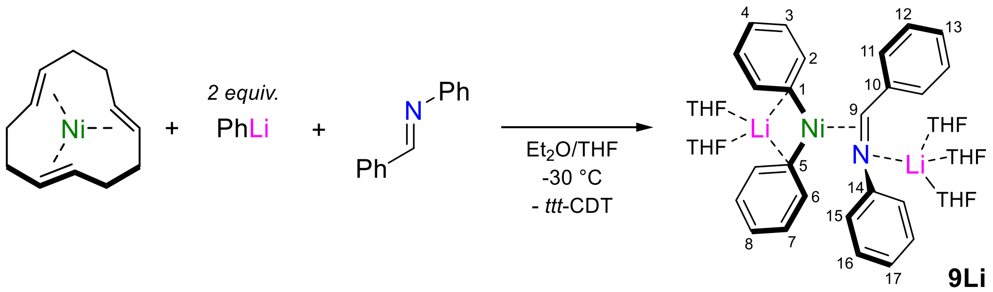

**9Li**

Ni(*ttt*-CDT) (22.1 mg, 0.1 mmol) was dissolved in $Et_2O$ (1 mL) and cooled to -30 °C. PhLi (17.1 mg, 0.2 mmol) was added and the solution was warmed to room temperature, stirred for 2-3 minutes, then recooled to -30 °C. *N*-benzylideneaniline (18.1 mg, 0.1 mmol) was added followed by THF (0.1 mL) and the red reaction mixture was stirred at -30 °C for 5 minutes, then filtered and stored at -30 °C for crystallisation *via* pentane vapour diffusion. After 72 hours, the supernatant was decanted from the bright red crystals, which were washed with cold pentane (2 × 0.5 mL) and dried under argon. Yield – 31 mg (50%).

**$^1$H NMR** (300.1 MHz, THF-$d_8$): δ 8.00 (d, *J* = 6.4 Hz, 2H, *H*2/6), 7.87 (d, *J* = 6.4 Hz, 2H, *H*2/6), 6.92–6.83 (m, 2H + 2H, *H*3/7 + *H*15), 6.77–6.68 (m, 2H + 2H + 2H +1H, *H*3/7 + *H*11, *H*12, *H*4/8), 6.68–6.59 (m, 2H, *H*16), 6.59–6.48 (m, 1H + 1H, *H*4/8 + *H*13), 6.38 (t, *J* = 7.1 Hz, 1H, *H*17), 3.81 (s, 1H, *H*9), 3.62 (THF), 1.77 (THF).

**$^7$Li NMR** (116.6 MHz, THF-$d_8$): δ -0.12 (s).

**$^{13}C\{^1H\}$ NMR** (75.5 MHz, THF-$d_8$): δ 181.8 (C1/5), 179.7 (C1/5), 161.0 (C14), 153.6 (C10), 143.7 (C2/6), 142.4 (C2/6), 128.6 (C15), 127.8 (C12), 126.0 (C3/7), 125.7 (C3/7), 124.8 (C11), 121.7 (C4/8), 120.9 (C16), 120.3 (C13), 119.8 (C4/8), 114.4 (C17), 67.6 (THF), 53.7 (C9), 25.5 (THF).

**Elemental Analysis:** Calculated for $C_{45}H_{61}Li_2NNiO_5$: C, 70.33; H, 8.00; N, 1.82. Found: C, 70.30; H, 8.07; N, 1.74.

*N.B.* NMR spectroscopy and elemental analysis consistent with three molecules of coordinated THF.

**Synthesis of [$Li_2(THF)_3Ph_2Ni(\eta^2$-$Ph_2CHCN)]_2$ (10Li)**

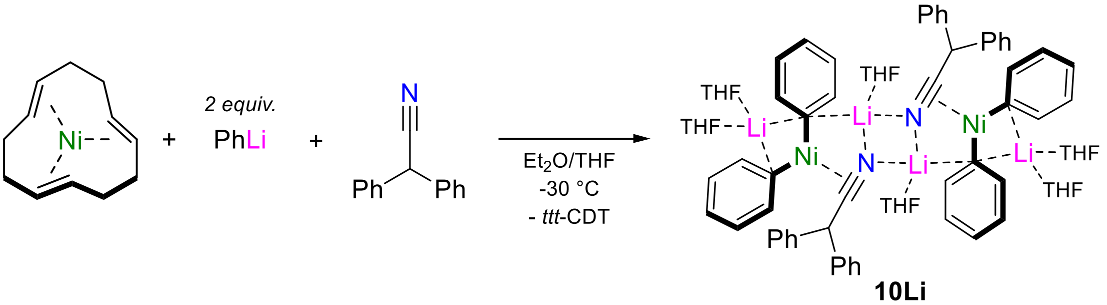


**10Li**

Ni(*ttt*-CDT) (22.1 mg, 0.1 mmol) was dissolved in $Et_2O$ (1 mL) and cooled to -30 °C. PhLi (17.1 mg, 0.2 mmol) was added and the solution was warmed to room temperature, stirred for 2-3 minutes, then recooled to -30 °C. Diphenylacetonitrile (19.3 mg, 0.1 mmol) was added slowly as a chilled solution in THF (1 mL) and the amber reaction mixture was stirred at -30 °C for 5 minutes, then filtered and stored at -30 °C for crystallisation *via* pentane vapour diffusion. After 48 hours, the supernatant was decanted from the bright yellow crystals, which were washed with cold pentane (2 × 0.5 mL) and dried under argon. Yield – 27 mg (42%).

**$^1H$ NMR** (400.1 MHz, THF-$d_8$, -20 °C): δ 8.10 (d, *J* = 6.9 Hz, 2H), 7.89 (d, *J* = 6.7 Hz, 2H), 7.52–7.35 (m, 4H), 7.05 (m, 4H), 6.98 (m, 2H), 6.90 (br, 2H), 6.72 (br, 3H), 6.60–6.47 (m, 2H), 5.29 (br, 1H), 3.62 (THF), 1.78 (THF).

**$^7Li$ NMR** (155.5 MHz, THF-$d_8$, -20 °C): δ 0.33 (br).

*N.B.* Due to poor solution-state stability and low solubility at low temperatures, suitable $^{13}C\{^1H\}$ NMR spectral data could not be obtained.

**Elemental Analysis:** Calculated for $C_{38}H_{45}Li_2NNiO_3$: C, 71.72; H, 7.13; N, 2.20. Found: C, 71.01; H, 7.09; N, 2.09.

**Synthesis of $Li(THF)_3(PPh_3)_2(CO)_3Ni_2(\mu$-$PPh_2)$ (11Li)**

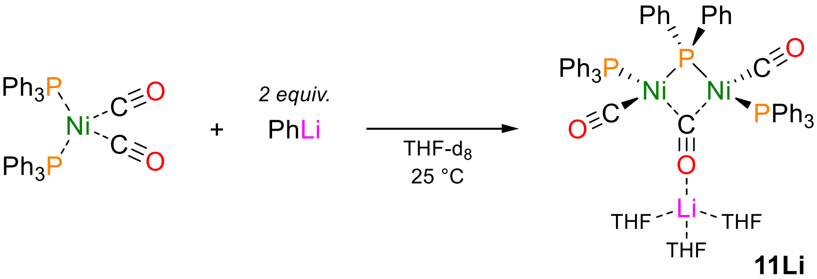


**11Li**

$(Ph_3P)_2Ni(CO)_2$ (25.5 mg, 0.04 mmol) was dissolved in THF-$d_8$ (0.5 mL) and PhLi (6.8 mg, 0.08 mmol) was added at room temperature resulting in a colour change from colourless to deep red. New signals were observed in the $^{31}P$ NMR spectrum at δ 45.5 and 28.7 ppm which grew in intensity over the course of 2 days as the signal for the starting material (δ 32.6) disappeared. Additional signals at δ 31.5 (unidentified species) and -5.5 ($Ph_3P$) were also observed, alongside numerous minor signals. The solution was decanted into a vial and stored at -30 °C for crystallisation *via* pentane vapour diffusion. After 72 hours, red crystals of **11Li** suitable for X-ray diffraction were obtained. The supernatant was decanted and the solids were washed with cold pentane (0.5 mL) and dried under argon. Yield – 6 mg (26%).

**$^1$H NMR** (300.1 MHz, THF-$d_8$): δ 7.34 (br, 12H), 7.20–7.02 (m, 22H), 6.48 (m, 6H).

**$^7$Li NMR** (116.6 MHz, THF-$d_8$): δ -0.62 (br).

**$^{31}$P NMR** (121.5 MHz, THF-$d_8$): δ 45.7 (s, 1P), 28.7 (s, 2P).

## Variable Temperature NMR Spectroscopy

### [Li(THF)$_2$Ph$_2$Ni($\eta^2$-coronene)][Li(THF)$_4$] (7Li)

15 mg of [Li(THF)$_2$Ph$_2$Ni($\eta^2$-coronene)][Li(THF)$_4$] (**7Li**) was dissolved in THF-$d_8$ (0.5 mL) and analysed by $^1$H and $^7$Li NMR spectroscopy at +20 °C, +10 °C, 0 °C, -10 °C, -20 °C, -30 °C and -40 °C (**Figures S1–2**).

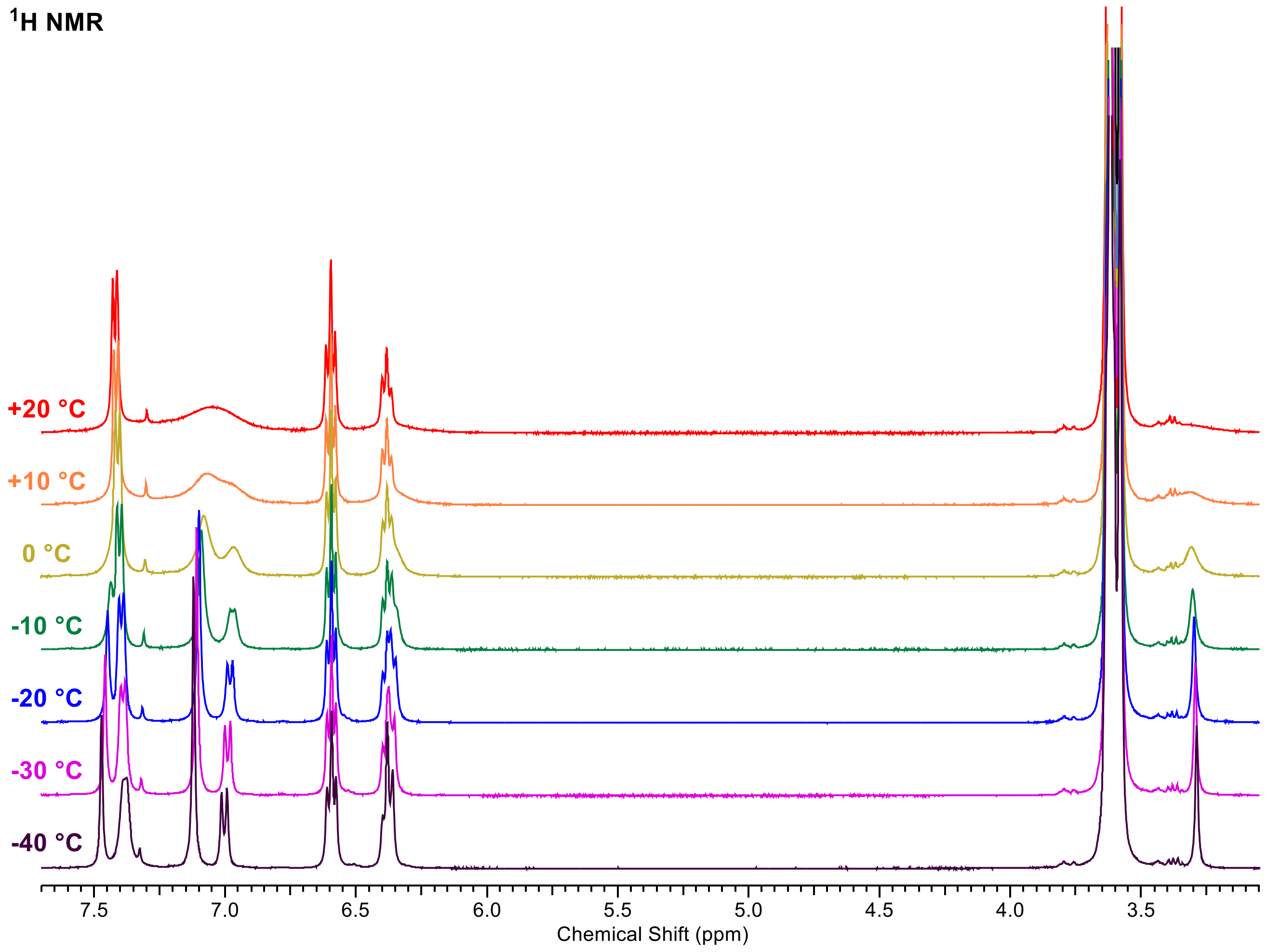


**Figure S1:** Stacked $^1$H NMR spectra at variable temperatures for [Li(THF)$_2$Ph$_2$Ni($\eta^2$-coronene)][Li(THF)$_4$] (**7Li**).

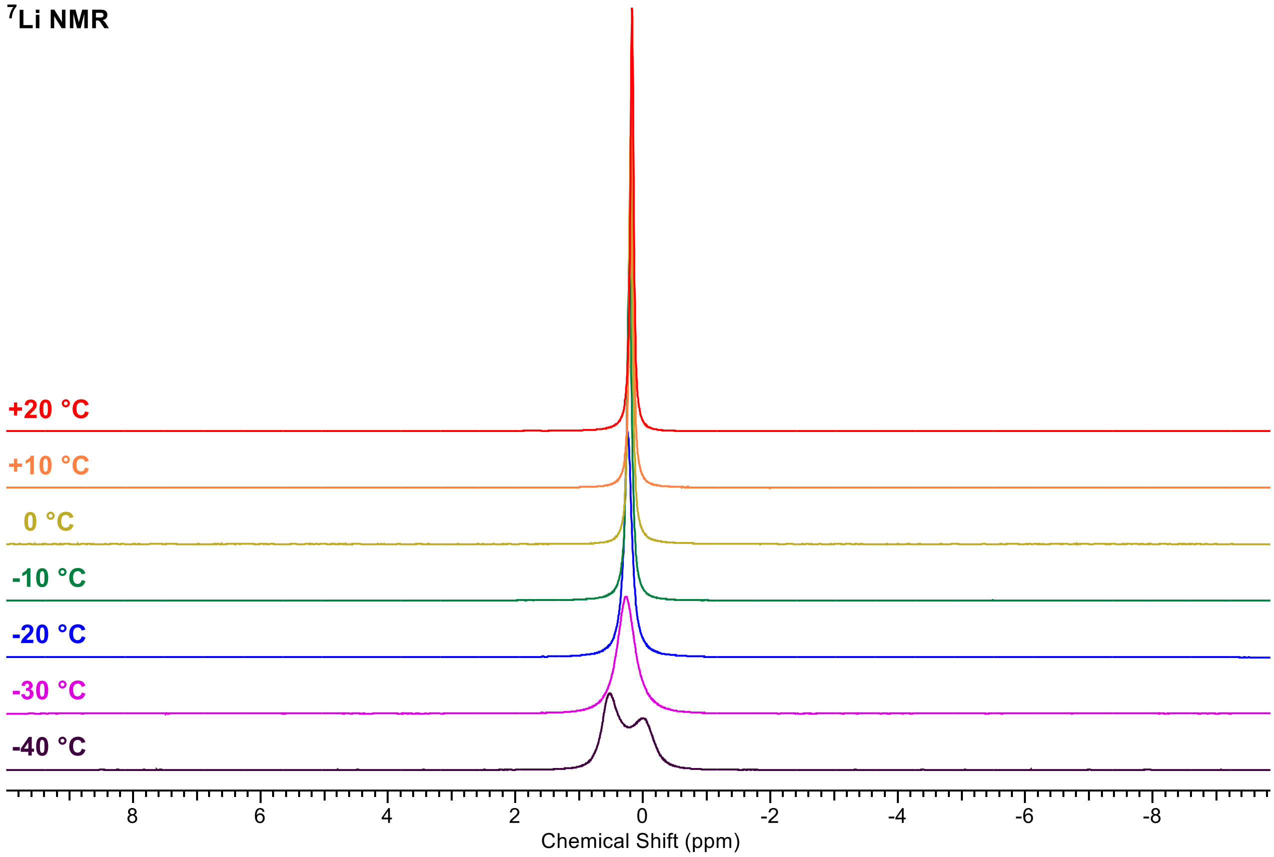


**Figure S2:** Stacked $^{7}Li$ NMR spectra at variable temperatures for $[Li(THF)_2Ph_2Ni(\eta^2\text{-coronene})][Li(THF)_4]$ (**7Li**).

## DOSY NMR Spectroscopy

Estimated molecular weights (MW) were calculated from the diffusion coefficients established from the $^{1}H$ DOSY NMR spectrum using Stalke's external calibration curve (ECC) method[5–7] and using tetramethylsilane as an internal standard. Samples were prepared by dissolving 0.0075 mmol each of complex and internal standard in 500 µL of THF-$d_8$ to give a concentration of 15 mM.

### $[Na_2(THF)_3Ph_2Ni(\eta^2\text{-phenanthrene})]_2$ (5Na)

Predicted molecular weight for $Na_2(THF)_4Ph_2Ni(\eta^2\text{-phenanthrene})$ = 725.55 g mol$^{-1}$. Estimated molecular weights determined from diffusion coefficients = 683 g mol$^{-1}$ (Merge), 6% difference; 626 g mol$^{-1}$ (DSE), 16% difference (**Figure S3**).

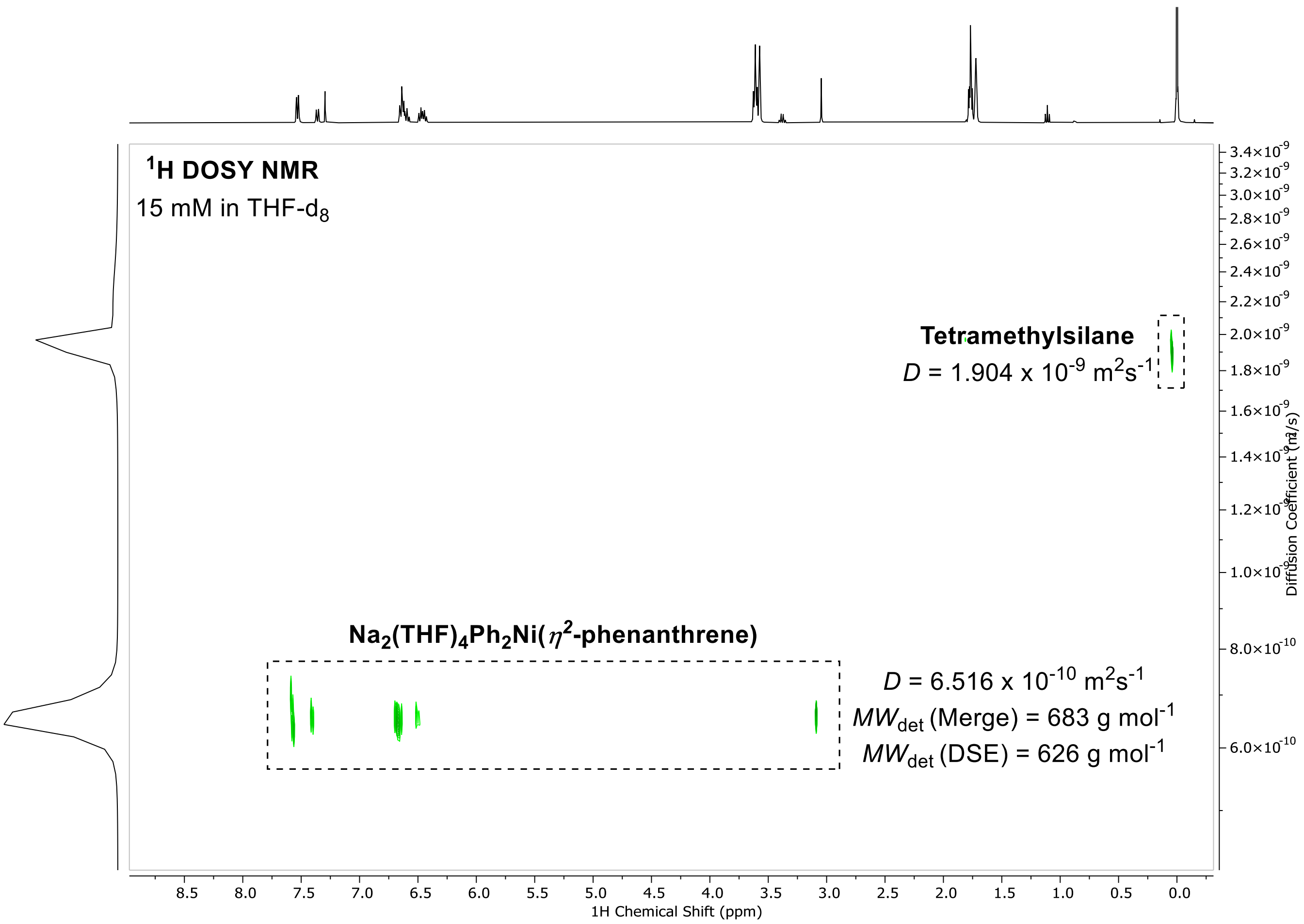


**Figure S3:** $^{1}$H DOSY NMR spectrum of $[Na_2(THF)_3Ph_2Ni(\eta^2\text{-phenanthrene})]_2$ (**5Na**) in THF-$d_8$.

**$[K_2(THF)_1Ph_2Ni(\eta^2\text{-phenanthrene})]_\infty$ (5K)**

Predicted molecular weight for $K_2(THF)_4Ph_2Ni(\eta^2\text{-phenanthrene})$ = 757.76 g mol$^{-1}$. Estimated molecular weights determined from diffusion coefficients = 713 g mol$^{-1}$ (Merge), 6% difference; 652 g mol$^{-1}$ (DSE), 16% difference (**Figure S4**).

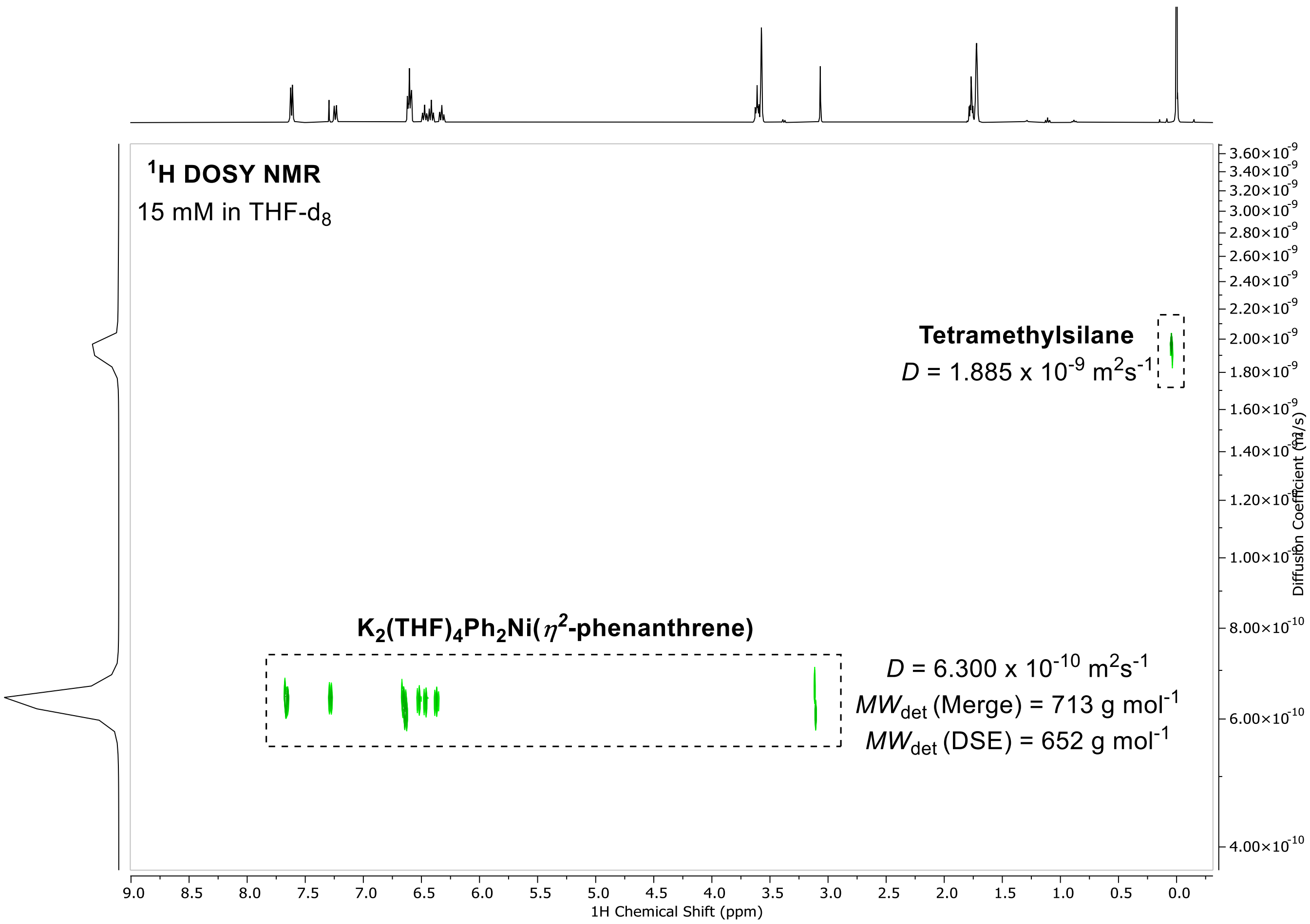


**Figure S4:** [1]H DOSY NMR spectrum of $[K_2(THF)_1Ph_2Ni(\eta^2\text{-phenanthrene})]_\infty$ (**5K**) in THF-$d_8$.

## EPR Spectroscopy

Samples for EPR spectroscopy were prepared by dissolving approximately 1 mg of compound in 1 mL of THF and transferring 50 µL to a 1.5 mm inset capillary that was placed within a quartz EPR tube and sealed.

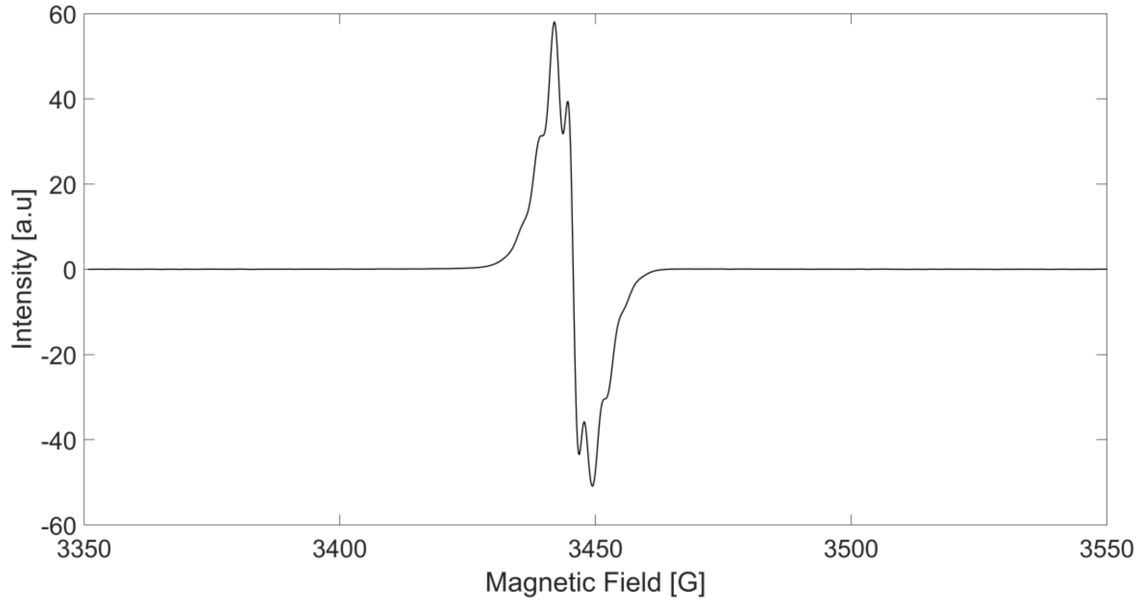

**Figure S5:** EPR spectrum of crystalline [Li(THF)$_2$Ph$_2$Ni($\eta^3$-perylene)][Li(THF)$_4$] (**6Li**) contaminated with the perylene radical anion.

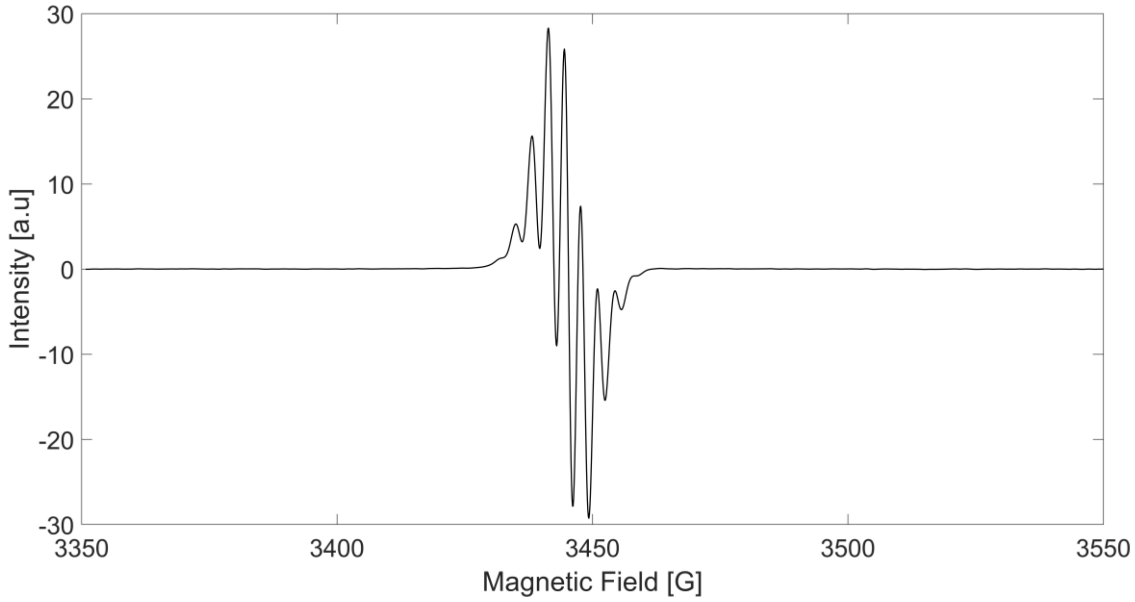


**Figure S6:** EPR spectrum of the solids isolated during the attempted synthesis of Na$_2$(solv)$_n$Ph$_2$Ni(perylene).

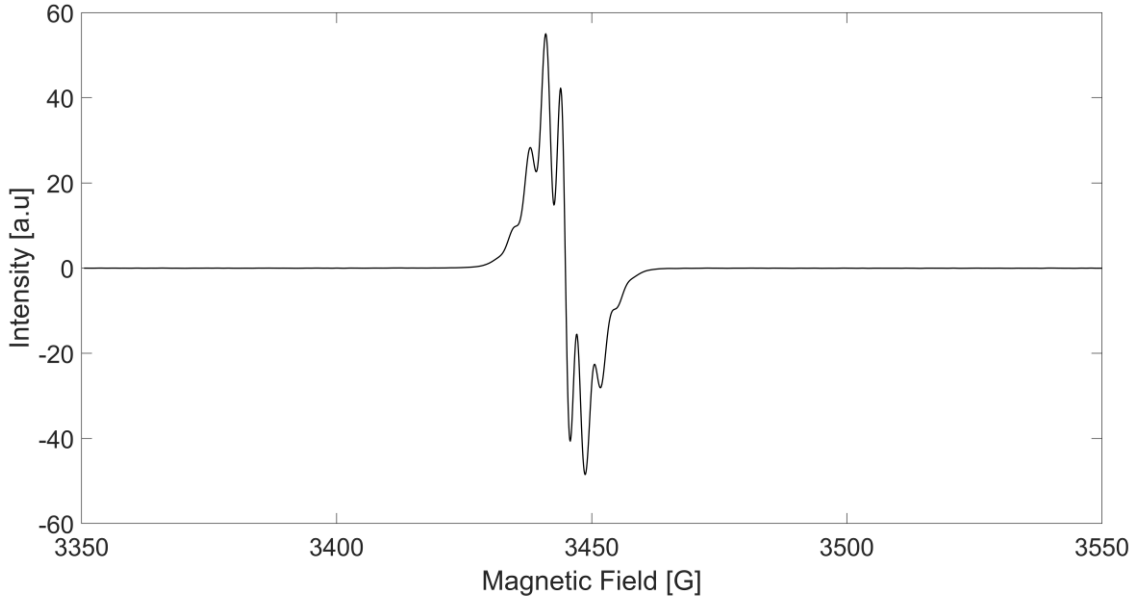


**Figure S7:** EPR spectrum of isolated [perylene][K(DME)$_4$] during the attempted synthesis of K$_2$(solv)$_n$Ph$_2$Ni(perylene).

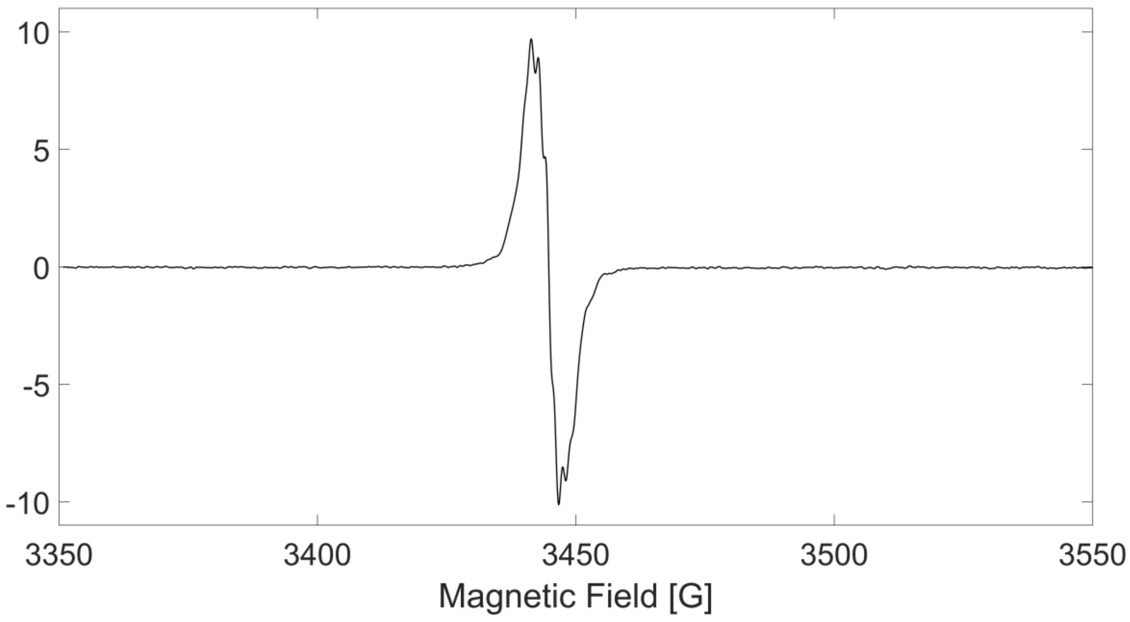


**Figure S8:** EPR spectrum of crystalline $K_2(DME)_4Ph_2Ni(\eta^2$-coronene) (**7K**) contaminated with the coronene radical anion.

### X-Ray Crystallography

The crystal structures of all novel compounds have been deposited into the Cambridge Crystallographic Data Centre (CCDC) and have been assigned the following numbers: 2326086–2326102. Selected crystallographic and refinement parameters are presented below (**Tables S1–5**). In all cases, crystals immersed in an inert parabar oil were mounted at low temperatures and transferred into the nitrogen stream (100 or 173 K). Perfluorinated oils must be avoided for the alkali-metal nickelates.

All measurements were made on a *RIGAKU Synergy S* area-detector diffractometer using mirror optics monochromated Cu Kα radiation (λ = 1.54184 Å) or on a *RIGAKU XtaLAB Synergy R*, HyPix-Arc 100 area-detector diffractometer using mirror optics monochromated Mo Kα radiation (λ = 0.71073 Å). Data reduction was performed using the *CrysAlisPro* program.[8] The intensities were corrected for Lorentz and polarization effects, and an absorption correction based on the Gaussian method using SCALE3 ABSPACK in *CrysAlisPro* was applied. The structure was solved by direct methods or intrinsic phasing using *SHELXT*,[9] which revealed the positions of all non-hydrogen atoms of the compounds. All non-hydrogen atoms were refined anisotropically. H-atoms were assigned in geometrically calculated positions and refined using a riding model where each H-atom was assigned a fixed isotropic displacement parameter with a value equal to 1.2Ueq of its parent atom (1.5Ueq for methyl groups). Refinement of the structure was carried out on F2 using full-matrix least-squares procedures, which minimized the function $\Sigma w(F_o^2 - F_c^2)^2$. The weighting scheme was based on counting statistics and included a factor to downweight the intense reflections. All calculations were performed using the *SHELXL*-2014/7 program[10] in *OLEX2*.[11]

For [(*ttt*-CDT)NiPh][$Li(12\text{-crown-}4)_2$] (**1**), a disorder model was used for parts of the *ttt*-CDT ligand and 12-crown-4 where the occupancies of each disorder component was refined through the use of a free variable. The sum of equivalent components was constrained to 1, i.e. 100%.

For $Li_4(TMEDA)_2(Et_2O)_2Ph_4Ni_2(\mu;\eta^2;\eta^2\text{-}C_6H_4)$ (**3**), a disorder model was used for parts of the coordinated $Et_2O$ and TMEDA where the occupancies of each disorder component was refined through the use of a free variable. The sum of equivalent components was constrained to 1, i.e. 100%.

For $Li_2(THF)_4Ph_2Ni(\eta^2$-anthracene) (**4Li**), a disorder model was used for the parts of the coordinated THF where the occupancies of each disorder component were refined with the use of a free variable. The sum of equivalent components was constraint to 1, i.e. 100%.

For $Na_2(TMEDA)_2Ph_2Ni(\eta^2$-anthracene) (**4Na**), a disorder model was used for the parts of the coordinated TMEDA where the occupancies of each disorder component was refined through the use of a free variable. The sum of equivalent components was constrained to 1, i.e. 100%.

For $K_2(PMDETA)_2Ph_2Ni(\eta^2$-anthracene) (**4K**), a disorder model was used for several parts of the structure where the occupancies of each disorder component was refined through the use of a free variable. The sum of equivalent components was constrained to 1, i.e. 100%.

For $Li_2(THF)_4Ph_2Ni(\eta^2$-phenanthrene) (**5Li**), a disorder model was used for parts of the coordinated THF where the occupancies of each disorder component was refined through the use of a free variable. The sum of equivalent components was constrained to 1, i.e. 100%.

For $[Na_2(THF)_3Ph_2Ni(\eta^2\text{-phenanthrene})]_2$ (**5Na**), a disorder model was used for parts of coordinated THF where the occupancies of each disorder component was refined through the use of a free variable. The sum of equivalent components was constrained to 1, i.e. 100%. Areas containing disordered solvents were found where a satisfactory solvent model could not be achieved, therefore, a solvent mask was used to include the contribution of electron density found in void areas into the calculated structure factor.

For $[K_2(THF)_1Ph_2Ni(\eta^2\text{-phenanthrene})]_\infty$ (**5K**), twinning could be detected where the second and third twin components corresponded to a rotation of -179.9272 degrees around [-0.00 -0.00 1.00] (reciprocal space), or [0.11 0.00 0.99] (direct space) for twin component 2 and -162.9797 degrees around [-0.11 -0.01 0.99] (reciprocal space), or [-0.20 -0.05 0.98] (direct space) for twin component 3. The refinement was performed against the reflection file containing detwinned data of all 3 components on a hkl 5 format. The final refined volume fractional contribution of twins 2 and 3 were 0.4870(9) and 0.02401(17) respectively.

For $[Li(THF)_2Ph_2Ni(\eta^3\text{-perylene})][Li(THF)_4]$ (**6Li**), a disorder model was used for coordinated THF molecules where the occupancies of each disorder component was refined through the use of a free variable. The sum of equivalent components was constrained to 1, i.e. 100%.

For $[Li(THF)_2Ph_2Ni(\eta^2\text{-coronene})][Li(THF)_4]$ (**7Li**), a disorder model was used for coordinated THF molecules where the occupancies of each disorder component was refined through the use of a free variable. The sum of equivalent components was constrained to 1, i.e. 100%.

For $[Li_2(THF)_3Ph_2Ni(\eta^2\text{-}Ph_2CO)]_2$ (**8Li**), a disorder model was used for parts of the coordinated THF where the occupancies of each disorder component was refined through the use of a free variable. The sum of equivalent components was constrained to 1, i.e. 100%. Areas containing disordered solvents were found

where a satisfactory solvent model could not be achieved, therefore, a solvent mask was used to include the contribution of electron density found in void areas into the calculated structure factor.

For $[K_2(DME)_3Ph_2Ni(\eta^2\text{-}Ph_2CO)]_2$ (**8K**), A disorder model was used for parts of the coordinated DME where the occupancies of each disorder component was refined through the use of a free variable. The sum of equivalent components was constrained to 1, i.e. 100%. Areas containing disordered solvents were found where a satisfactory solvent model could not be achieved, therefore, a solvent mask was used to include the contribution of electron density found in void areas into the calculated structure factor.

For $[Li_2(THF)_3Ph_2Ni(\eta^2\text{-}Ph_2CHCN)]_2$ (**10Li**), a disorder model was used for parts of the coordinated THF where the occupancies of each disorder component was refined through the use of a free variable. The sum of equivalent components was constrained to 1, i.e. 100%. Areas containing disordered solvents were found where a satisfactory solvent model could not be achieved, therefore, a solvent mask was used to include the contribution of electron density found in void areas into the calculated structure factor.

For $Li(THF)_3(PPh_3)_2(CO)_3Ni_2(\mu\text{-}PPh_2)$ (**11Li**), a disorder model was used for parts of the coordinated THF where the occupancies of each disorder component was refined through the use of a free variable. The sum of equivalent components was constrained to 1, i.e. 100%. Areas containing disordered solvents were found where a satisfactory solvent model could not be achieved, therefore, a solvent mask was used to include the contribution of electron density found in void areas into the calculated structure factor.

| Identification code | **1** | **3** | **4Li** | **4Na** |
|---|---|---|---|---|
| CCDC Number | 2326086 | 2326087 | 2326088 | 2326089 |
| Empirical formula | $C_{34}H_{55}LiNiO_8$ | $C_{50}H_{76}Li_4N_4Ni_2O_2$ | $C_{42}H_{52}Li_2NiO_4$ | $C_{38}H_{52}N_4Na_2Ni$ |
| Formula weight | 657.43 | 910.32 | 693.42 | 669.52 |
| Temperature/K | 100.01(10) | 173.00(10) | 173.00(10) | 100.00(10) |
| Crystal system | monoclinic | monoclinic | monoclinic | monoclinic |
| Space group | $P2_1/n$ | $P2_1/n$ | C2/c | $P2_1/n$ |
| a/Å | 15.15520(10) | 10.94240(10) | 23.83845(18) | 10.5871(2) |
| b/Å | 10.28020(10) | 20.8208(2) | 10.06099(9) | 22.0003(3) |
| c/Å | 21.0871(2) | 22.7000(2) | 31.2245(3) | 16.2275(2) |
| α/° | 90 | 90 | 90 | 90 |
| β/° | 93.3440(10) | 95.7330(10) | 92.8991(8) | 94.8440(10) |
| γ/° | 90 | 90 | 90 | 90 |
| Volume/Å$^3$ | 3279.74(5) | 5145.86(8) | 7479.25(11) | 3766.20(10) |
| Z | 4 | 4 | 8 | 4 |
| $\rho_{calc}$g/cm$^3$ | 1.331 | 1.175 | 1.232 | 1.181 |
| μ/mm$^{-1}$ | 0.641 | 0.771 | 1.048 | 0.568 |
| F(000) | 1416 | 1952 | 2960 | 1432 |
| Crystal size/mm$^3$ | 0.173 × 0.107 × 0.051 | 0.16 × 0.123 × 0.05 | 0.291 × 0.062 × 0.041 | 0.266 × 0.093 × 0.069 |
| Radiation | Mo Kα (λ = 0.71073) | Mo Kα (λ = 0.71073) | Cu Kα (λ = 1.54184) | Mo Kα (λ = 0.71073) |
| 2Θ range for data collection/° | 4.41 to 60.162 | 4.104 to 60.068 | 5.668 to 148.956 | 4.282 to 61.016 |
| Index ranges | -21 ≤ h ≤ 21, -14 ≤ k ≤ 14, -29 ≤ l ≤ 29 | -15 ≤ h ≤ 15, -29 ≤ k ≤ 29, -31 ≤ l ≤ 31 | -29 ≤ h ≤ 29, -12 ≤ k ≤ 12, -39 ≤ l ≤ 39 | -15 ≤ h ≤ 15, -31 ≤ k ≤ 31, -23 ≤ l ≤ 23 |
| Reflections collected | 191061 | 154783 | 75114 | 114953 |
| Independent reflections | 9636 [$R_{int}$ = 0.0343, $R_{sigma}$ = 0.0129] | 15039 [$R_{int}$ = 0.0334, $R_{sigma}$ = 0.0203] | 7639 [$R_{int}$ = 0.0328, $R_{sigma}$ = 0.0179] | 11482 [$R_{int}$ = 0.0436, $R_{sigma}$ = 0.0275] |
| Data/restraints/parameters | 9636/279/561 | 15039/245/717 | 7639/172/548 | 11482/100/486 |
| Goodness-of-fit on $F^2$ | 1.07 | 1.027 | 1.064 | 1.023 |
| Final R indexes [I>=2σ (I)] | $R_1$ = 0.0319, $wR_2$ = 0.0778 | $R_1$ = 0.0307, $wR_2$ = 0.0764 | $R_1$ = 0.0412, $wR_2$ = 0.1189 | $R_1$ = 0.0429, $wR_2$ = 0.0962 |
| Final R indexes [all data] | $R_1$ = 0.0360, $wR_2$ = 0.0794 | $R_1$ = 0.0419, $wR_2$ = 0.0809 | $R_1$ = 0.0498, $wR_2$ = 0.1260 | $R_1$ = 0.0672, $wR_2$ = 0.1051 |
| Largest diff. peak/hole / e Å$^{-3}$ | 0.46/-0.37 | 0.30/-0.24 | 0.45/-0.33 | 0.44/-0.31 |

**Table S1:** Crystal data and structure refinement details for compounds **1**, **3**, **4Li** and **4Na**.

| Identification code | **4K** | **5Li** | **5Na** | **5K** |
|---|---|---|---|---|
| CCDC Number | 2326090 | 2326091 | 2326092 | 2326093 |
| Empirical formula | $C_{44}H_{66}K_2N_6Ni$ | $C_{42}H_{52}Li_2NiO_4$ | $C_{76}H_{88}Na_4Ni_2O_6$ | $C_{30}H_{28}K_2NiO$ |
| Formula weight | 815.93 | 693.42 | 1306.84 | 541.43 |
| Temperature/K | 100.00(10) | 173.00(10) | 100.01(10) | 173.01(10) |
| Crystal system | monoclinic | monoclinic | triclinic | monoclinic |
| Space group | $P2_1/n$ | $P2_1/n$ | P-1 | $P2_1/c$ |
| a/Å | 19.97570(10) | 16.7487(2) | 12.22970(10) | 12.46834(19) |
| b/Å | 11.46590(10) | 12.4289(2) | 13.7829(2) | 9.88563(16) |
| c/Å | 21.46230(10) | 18.2015(3) | 21.3400(2) | 20.5241(3) |
| α/° | 90 | 90 | 106.7570(10) | 90 |
| β/° | 112.3960(10) | 101.5340(10) | 99.6030(10) | 93.8253(14) |
| γ/° | 90 | 90 | 92.9580(10) | 90 |
| Volume/Å$^3$ | 4544.93(6) | 3712.45(10) | 3377.08(7) | 2524.11(7) |
| Z | 4 | 4 | 2 | 4 |
| $\rho_{calc}$g/cm$^3$ | 1.192 | 1.241 | 1.285 | 1.425 |
| μ/mm$^{-1}$ | 2.517 | 0.562 | 0.635 | 1.12 |
| F(000) | 1752 | 1480 | 1384 | 1128 |
| Crystal size/mm$^3$ | 0.215 × 0.172 × 0.097 | 0.176 × 0.134 × 0.071 | 0.254 × 0.16 × 0.08 | 0.178 × 0.144 × 0.110 |
| Radiation | Cu Kα (λ = 1.54184) | Mo Kα (λ = 0.71073) | Mo Kα (λ = 0.71073) | Mo Kα (λ = 0.71073) |
| 2Θ range for data collection/° | 5.146 to 149.002 | 4.11 to 60.06 | 4.19 to 61.016 | 4.576 to 61.044 |
| Index ranges | -24 ≤ h ≤ 24, -14 ≤ k ≤ 13, -26 ≤ l ≤ 26 | -23 ≤ h ≤ 23, -17 ≤ k ≤ 17, -25 ≤ l ≤ 25 | -17 ≤ h ≤ 17, -19 ≤ k ≤ 19, -30 ≤ l ≤ 30 | -17 ≤ h ≤ 17, -14 ≤ k ≤ 14, -29 ≤ l ≤ 29 |
| Reflections collected | 102150 | 108687 | 204216 | 23178 |
| Independent reflections | 9294 [$R_{int}$ = 0.0240, $R_{sigma}$ = 0.0125] | 10860 [$R_{int}$ = 0.0355, $R_{sigma}$ = 0.0199] | 20582 [$R_{int}$ = 0.0484, $R_{sigma}$ = 0.0307] | 23178 [$R_{int}$ = ?, $R_{sigma}$ = 0.0390] |
| Data/restraints/parameters | 9294/1134/735 | 10860/136/495 | 20582/202/893 | 23178/0/309 |
| Goodness-of-fit on $F^2$ | 1.086 | 1.029 | 1.048 | 1.163 |
| Final R indexes [I>=2σ (I)] | $R_1$ = 0.0557, $wR_2$ = 0.1471 | $R_1$ = 0.0377, $wR_2$ = 0.0971 | $R_1$ = 0.0610, $wR_2$ = 0.1613 | $R_1$ = 0.0398, $wR_2$ = 0.1211 |
| Final R indexes [all data] | $R_1$ = 0.0583, $wR_2$ = 0.1494 | $R_1$ = 0.0463, $wR_2$ = 0.1012 | $R_1$ = 0.0884, $wR_2$ = 0.1769 | $R_1$ = 0.0631, $wR_2$ = 0.1279 |
| Largest diff. peak/hole / e Å$^{-3}$ | 0.65/-0.63 | 0.45/-0.33 | 1.34/-0.61 | 1.21/-1.42 |

**Table S2:** Crystal data and structure refinement details for compounds **4K**, **5Li**, **5Na** and **5K**.

| Identification code | **6Li** | [perylene][K(DME)$_4$] | **7Li** | **7K** |
|---|---|---|---|---|
| CCDC Number | 2326094 | 2326095 | 2326096 | 2326097 |
| Empirical formula | $C_{56}H_{69}Li_2NiO_6$ | $C_{36}H_{52}KO_8$ | $C_{60}H_{70}Li_2NiO_6$ | $C_{52}H_{62}K_2NiO_8$ |
| Formula weight | 910.7 | 651.87 | 959.75 | 951.92 |
| Temperature/K | 173.00(10) | 100.01(10) | 173.01(10) | 173.00(10) |
| Crystal system | monoclinic | monoclinic | orthorhombic | orthorhombic |
| Space group | $P2_1/c$ | C2/c | Pbca | $Pca2_1$ |
| a/Å | 17.8080(4) | 23.11620(10) | 13.7527(2) | 18.7188(3) |
| b/Å | 13.0662(2) | 13.75360(10) | 21.6279(4) | 26.1144(5) |
| c/Å | 22.5123(4) | 11.45110(10) | 34.0256(5) | 19.8447(3) |
| α/° | 90 | 90 | 90 | 90 |
| β/° | 111.093(2) | 96.5740(10) | 90 | 90 |
| γ/° | 90 | 90 | 90 | 90 |
| Volume/Å$^3$ | 4887.25(17) | 3616.72(4) | 10120.6(3) | 9700.7(3) |
| Z | 4 | 4 | 8 | 8 |
| $\rho_{calc}$g/cm$^3$ | 1.238 | 1.197 | 1.26 | 1.304 |
| μ/mm$^{-1}$ | 0.447 | 1.67 | 0.435 | 2.532 |
| F(000) | 1948 | 1404 | 4096 | 4032 |
| Crystal size/mm$^3$ | 0.528 × 0.451 × 0.298 | 0.169 × 0.126 × 0.109 | 0.277 × 0.1 × 0.067 | 0.173 × 0.114 × 0.063 |
| Radiation | Mo Kα (λ = 0.71073) | Cu Kα (λ = 1.54184) | Mo Kα (λ = 0.71073) | Cu Kα (λ = 1.54184) |
| 2Θ range for data collection/° | 4.786 to 61.014 | 7.492 to 148.994 | 4.248 to 61.016 | 7.322 to 148.98 |
| Index ranges | -25 ≤ h ≤ 25, -18 ≤ k ≤ 18, -32 ≤ l ≤ 32 | -23 ≤ h ≤ 28, -17 ≤ k ≤ 17, -14 ≤ l ≤ 14 | -19 ≤ h ≤ 19, -30 ≤ k ≤ 30, -46 ≤ l ≤ 48 | -23 ≤ h ≤ 22, -31 ≤ k ≤ 32, -24 ≤ l ≤ 20 |
| Reflections collected | 145032 | 36959 | 242758 | 87034 |
| Independent reflections | 14918 [$R_{int}$ = 0.0386, $R_{sigma}$ = 0.0226] | 3694 [$R_{int}$ = 0.0296, $R_{sigma}$ = 0.0148] | 15457 [$R_{int}$ = 0.0536, $R_{sigma}$ = 0.0282] | 17300 [$R_{int}$ = 0.0773, $R_{sigma}$ = 0.0557] |
| Data/restraints/parameters | 14918/198/699 | 3694/0/308 | 15457/110/708 | 17300/7/1168 |
| Goodness-of-fit on F$^2$ | 1.031 | 1.06 | 1.034 | 1.026 |
| Final R indexes [I>=2σ (I)] | $R_1$ = 0.0497, $wR_2$ = 0.1336 | $R_1$ = 0.0261, $wR_2$ = 0.0714 | $R_1$ = 0.0460, $wR_2$ = 0.1154 | $R_1$ = 0.0486, $wR_2$ = 0.1061 |
| Final R indexes [all data] | $R_1$ = 0.0697, $wR_2$ = 0.1447 | $R_1$ = 0.0271, $wR_2$ = 0.0720 | $R_1$ = 0.0730, $wR_2$ = 0.1276 | $R_1$ = 0.0855, $wR_2$ = 0.1253 |
| Largest diff. peak/hole / e Å$^{-3}$ | 0.46/-0.24 | 0.21/-0.21 | 0.60/-0.37 | 0.34/-0.42 |
| Flack Parameter | - | - | - | 0.003(6) |

**Table S3:** Crystal data and structure refinement details for compounds **6Li**, [perylene][K(DME)$_4$], **7Li** and **7K**.

| Identification code | **8Li** | **8K** | **9Li** |
|---|---|---|---|
| CCDC Number | 2326098 | 2326099 | 2326100 |
| Empirical formula | $C_{74}H_{88}Li_4Ni_2O_8$ | $C_{37}H_{50}K_2NiO_7$ | $C_{45}H_{61}Li_2NO_5Ni$ |
| Formula weight | 1250.62 | 743.68 | 768.53 |
| Temperature/K | 100.01(10) | 100.15 | 173.01(10) |
| Crystal system | triclinic | monoclinic | monoclinic |
| Space group | P-1 | I2/a | $P2_1/n$ |
| a/Å | 18.8977(2) | 26.5914(4) | 10.61066(4) |
| b/Å | 19.4534(2) | 15.6226(2) | 10.14415(4) |
| c/Å | 22.3454(4) | 19.9575(3) | 39.27151(16) |
| α/° | 80.1410(10) | 90 | 90 |
| β/° | 87.6060(10) | 98.4080(10) | 94.5490(4) |
| γ/° | 89.5510(10) | 90 | 90 |
| Volume/Å$^3$ | 8086.34(19) | 8201.8(2) | 4213.72(3) |
| Z | 4 | 8 | 4 |
| $\rho_{calc}$g/cm$^3$ | 1.027 | 1.205 | 1.211 |
| μ/mm$^{-1}$ | 0.51 | 0.717 | 1.001 |
| F(000) | 2656 | 3152 | 1648 |
| Crystal size/mm$^3$ | 0.197 × 0.154 × 0.046 | 0.393 × 0.243 × 0.168 | 0.236 × 0.174 × 0.076 |
| Radiation | Mo Kα (λ = 0.71073) | Mo Kα (λ = 0.71073) | Cu Kα (λ = 1.54184) |
| 2Θ range for data collection/° | 4.208 to 60.068 | 4.334 to 60.068 | 4.514 to 148.988 |
| Index ranges | -26 ≤ h ≤ 26, -27 ≤ k ≤ 27, -31 ≤ l ≤ 31 | -37 ≤ h ≤ 37, -22 ≤ k ≤ 22, -28 ≤ l ≤ 28 | -10 ≤ h ≤ 13, -12 ≤ k ≤ 12, -49 ≤ l ≤ 49 |
| Reflections collected | 486489 | 121855 | 87197 |
| Independent reflections | 47321 [$R_{int}$ = 0.0640, $R_{sigma}$ = 0.0441] | 12004 [$R_{int}$ = 0.0359, $R_{sigma}$ = 0.0196] | 8616 [$R_{int}$ = 0.0237, $R_{sigma}$ = 0.0117] |
| Data/restraints/parameters | 47321/461/1816 | 12004/80/487 | 8616/0/491 |
| Goodness-of-fit on F$^2$ | 1.035 | 1.036 | 1.057 |
| Final R indexes [I>=2σ (I)] | $R_1$ = 0.0534, $wR_2$ = 0.1316 | $R_1$ = 0.0370, $wR_2$ = 0.0933 | $R_1$ = 0.0388, $wR_2$ = 0.1080 |
| Final R indexes [all data] | $R_1$ = 0.0848, $wR_2$ = 0.1440 | $R_1$ = 0.0460, $wR_2$ = 0.0970 | $R_1$ = 0.0402, $wR_2$ = 0.1092 |
| Largest diff. peak/hole / e Å$^{-3}$ | 0.84/-0.53 | 0.71/-0.34 | 0.49/-0.38 |

**Table S4:** Crystal data and structure refinement details for compounds **8Li**, **8K** and **9Li**.

| Identification code | **10Li** | **11Li** |
|---|---|---|
| CCDC Number | 2326101 | 2326102 |
| Empirical formula | $C_{76}H_{90}Li_4N_2Ni_2O_6$ | $C_{74}H_{88}Li_4Ni_2O_8$ |
| Formula weight | 1272.67 | 1250.62 |
| Temperature/K | 100.00(10) | 100.01(10) |
| Crystal system | monoclinic | triclinic |
| Space group | $P2_1/n$ | P-1 |
| a/Å | 13.77390(10) | 18.8977(2) |
| b/Å | 19.3181(2) | 19.4534(2) |
| c/Å | 14.30610(10) | 22.3454(4) |
| α/° | 90 | 80.1410(10) |
| β/° | 93.2280(10) | 87.6060(10) |
| γ/° | 90 | 89.5510(10) |
| Volume/Å$^3$ | 3800.61(6) | 8086.34(19) |
| Z | 2 | 4 |
| $\rho_{calc}$g/cm$^3$ | 1.112 | 1.027 |
| μ/mm$^{-1}$ | 0.543 | 0.51 |
| F(000) | 1352 | 2656 |
| Crystal size/mm$^3$ | 0.261 × 0.159 × 0.108 | 0.197 × 0.154 × 0.046 |
| Radiation | Mo Kα (λ = 0.71073) | Mo Kα (λ = 0.71073) |
| 2Θ range for data collection/° | 4.216 to 60.444 | 4.208 to 60.068 |
| Index ranges | -19 ≤ h ≤ 19, -27 ≤ k ≤ 27, -20 ≤ l ≤ 20 | -26 ≤ h ≤ 26, -27 ≤ k ≤ 27, -31 ≤ l ≤ 31 |
| Reflections collected | 225483 | 486489 |
| Independent reflections | 11310 [$R_{int}$ = 0.0408, $R_{sigma}$ = 0.0151] | 47321 [$R_{int}$ = 0.0640, $R_{sigma}$ = 0.0441] |
| Data/restraints/parameters | 11310/76/444 | 47321/461/1816 |
| Goodness-of-fit on F$^2$ | 1.049 | 1.035 |
| Final R indexes [I>=2σ (I)] | $R_1$ = 0.0394, $wR_2$ = 0.1053 | $R_1$ = 0.0534, $wR_2$ = 0.1316 |
| Final R indexes [all data] | $R_1$ = 0.0447, $wR_2$ = 0.1079 | $R_1$ = 0.0848, $wR_2$ = 0.1440 |
| Largest diff. peak/hole / e Å$^{-3}$ | 0.92/-0.31 | 0.84/-0.53 |

**Table S5:** Crystal data and structure refinement details for compounds **10Li** and **11Li**.

**Solid-State Structure of [(*ttt*-CDT)NiPh][Li(12-crown-4)$_2$] (1)**

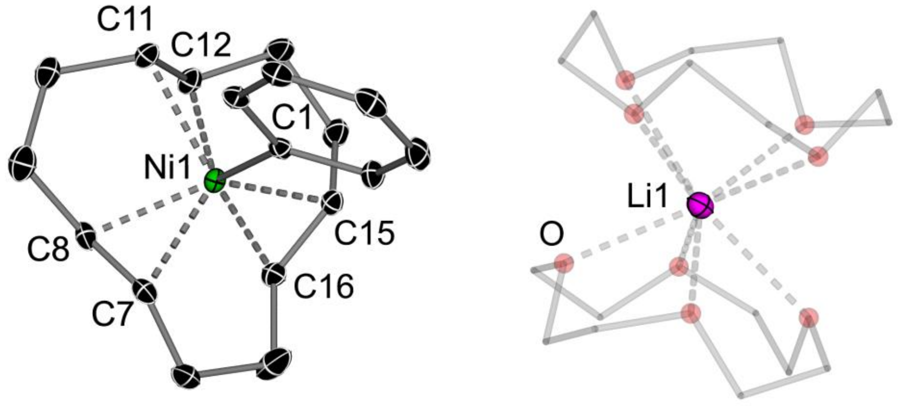


**Figure S9:** Solid-state structure of [(*ttt*-CDT)NiPh][Li(12-crown-4)$_2$] (**1**). Thermal ellipsoids shown at 30% probability. Hydrogen atoms and disordered components removed and coordinated 12-crown-4 shown as wireframes for clarity. Selected bond lengths [Å]: Ni1–C1 2.024(1); Ni1–C7 2.077(2); Ni1–C8 2.056(2); Ni1–C11 2.106(2); Ni1–C12 2.087(2); Ni1–C15 2.114(2); Ni1–C16 2.083(2); C7–C8 1.384(3); C11–C12 1.386(3); C15–C16 1.384(3).

**Solid-State Structure of $Li_4(TMEDA)_2(Et_2O)_2Ph_4Ni_2$(*μ;η*$^2$*;η*$^2$-$C_6H_4$) (3)**

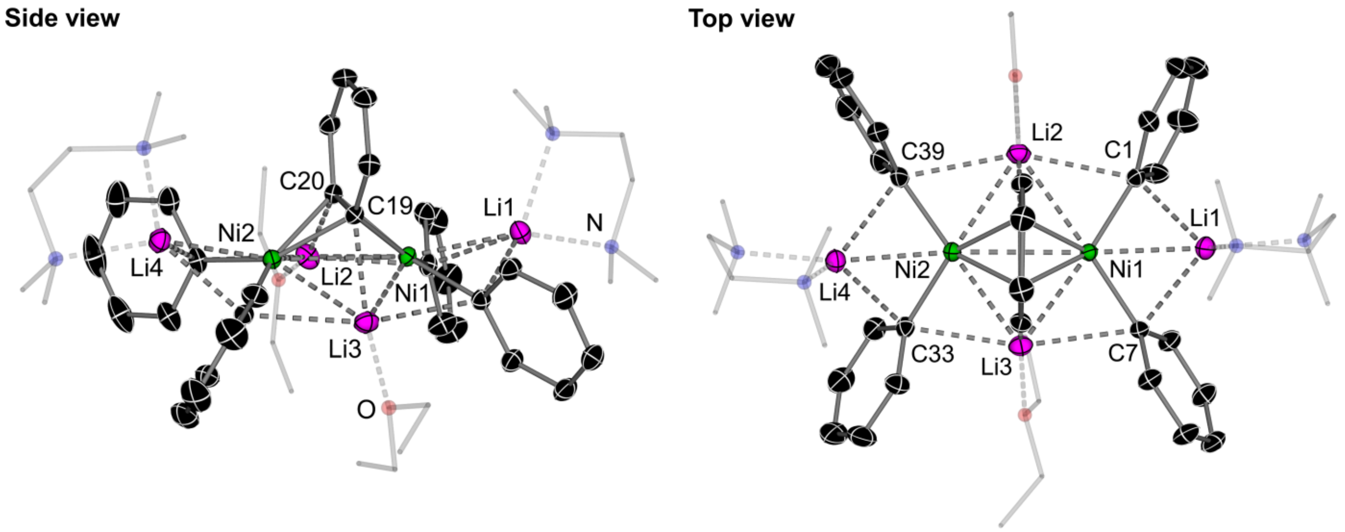


**Figure S10:** Solid-state structure of $Li_4(TMEDA)_2(Et_2O)_2Ph_4Ni_2$(*μ*;*η*$^2$;*η*$^2$-$C_6H_4$) (**3**). Thermal ellipsoids shown at 30% probability. Hydrogen atoms and disordered components removed and coordinated TMEDA and $Et_2O$ shown as wireframes for clarity. Selected bond lengths [Å]: Ni1–C1 1.975(1); Ni1–C7 1.983(1); Ni2–C33 1.973(1); Ni2–C39 1.971(1); Ni1–C19 1.946(1); Ni1–C20 1.966(1); Ni2–C19 1.952(1); Ni2–C20 1.955(1); Ni1–Ni2 2.8538(6); C19–C20 1.424(2).

**Solid-State Structure of $Li_2(THF)_4Ph_2Ni(\eta^2$-anthracene) (4Li)**

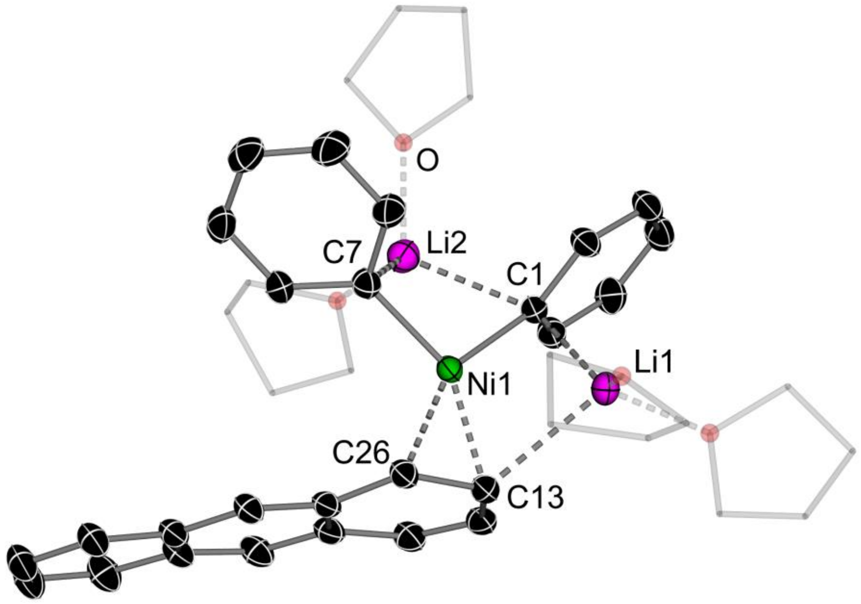


**Figure S11:** Solid-state structure of $Li_2(THF)_4Ph_2Ni(\eta^2$-anthracene) (**4Li**). Thermal ellipsoids shown at 30% probability. Hydrogen atoms and disordered components removed and coordinated THF shown as wireframes for clarity. Selected bond lengths [Å]: Ni1–C1 1.966(2); Ni1–C7 1.945(2); Ni1–C13 1.984(2); Ni1–C26 1.986(2); C13–C16 1.462(2).

**Solid-State Structure of $Na_2(TMEDA)_2Ph_2Ni(\eta^2$-anthracene) (4Na)**

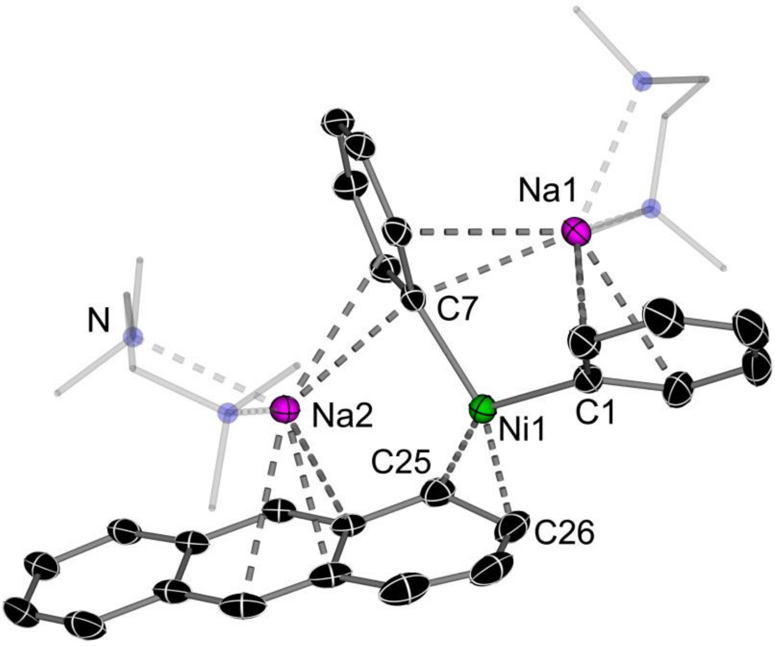


**Figure S12:** Solid-state structure of $Na_2(TMEDA)_2Ph_2Ni(\eta^2$-anthracene) (**4Na**). Thermal ellipsoids shown at 30% probability. Hydrogen atoms and disordered components removed and coordinated TMEDA shown as wireframes for clarity. Selected bond lengths [Å]: Ni1–C1 1.964(2); Ni1–C7 1.949(1); Ni1–C25 2.057(2); Ni1–1.954(2); C25–C26 1.449(2).

**Solid-State Structure of $K_2(PMDETA)_2Ph_2Ni(\eta^2$-anthracene) (4K)**

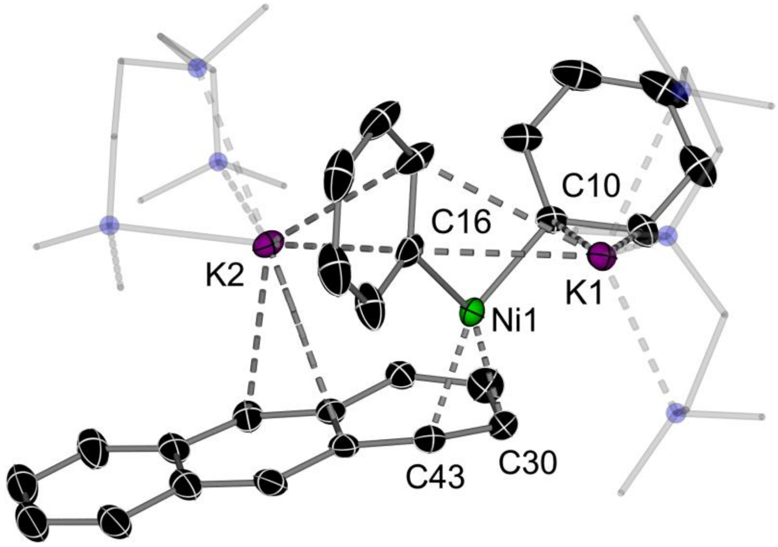


**Figure S13:** Solid-state structure of $K_2(PMDETA)_2Ph_2Ni(\eta^2$-anthracene) (**4K**). Thermal ellipsoids shown at 30% probability. Hydrogen atoms and disordered components removed and coordinated PMDETA shown as wireframes for clarity. Selected bond lengths [Å]: Ni1–C10 1.946(3); Ni1–C16 2.124(3); Ni1–C30 1.886(4); Ni1–C43 2.054(5); C30–C43 1.397(7).

**Solid-State Structure of $Li_2(THF)_4Ph_2Ni(\eta^2$-phenanthrene) (5Li)**

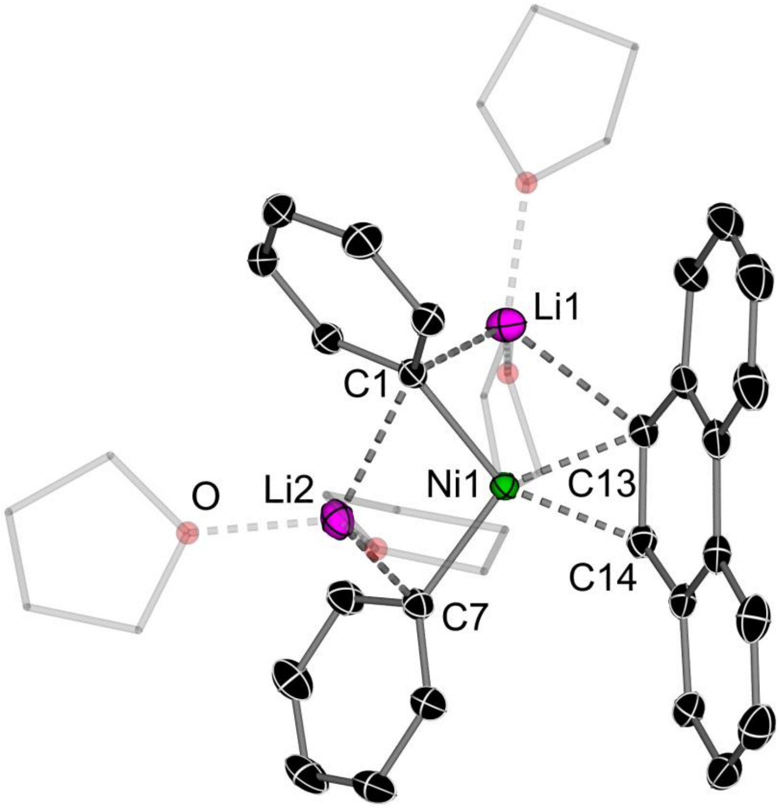


**Figure S14:** Solid-state structure of $Li_2(THF)_4Ph_2Ni(\eta^2$-phenanthrene) (**5Li**). Thermal ellipsoids shown at 30% probability. Hydrogen atoms and disordered components removed and coordinated THF shown as wireframes for clarity. Selected bond lengths [Å]: Ni1–C1 1.955(1); Ni1–C7 1.952(2); Ni1–C13 1.983(2); Ni1–C14 1.976(1); C13–C14 1.453(2).

**Solid-State Structure of $[Na_2(THF)_3Ph_2Ni(\eta^2\text{-phenanthrene})]_2$ (5Na)**

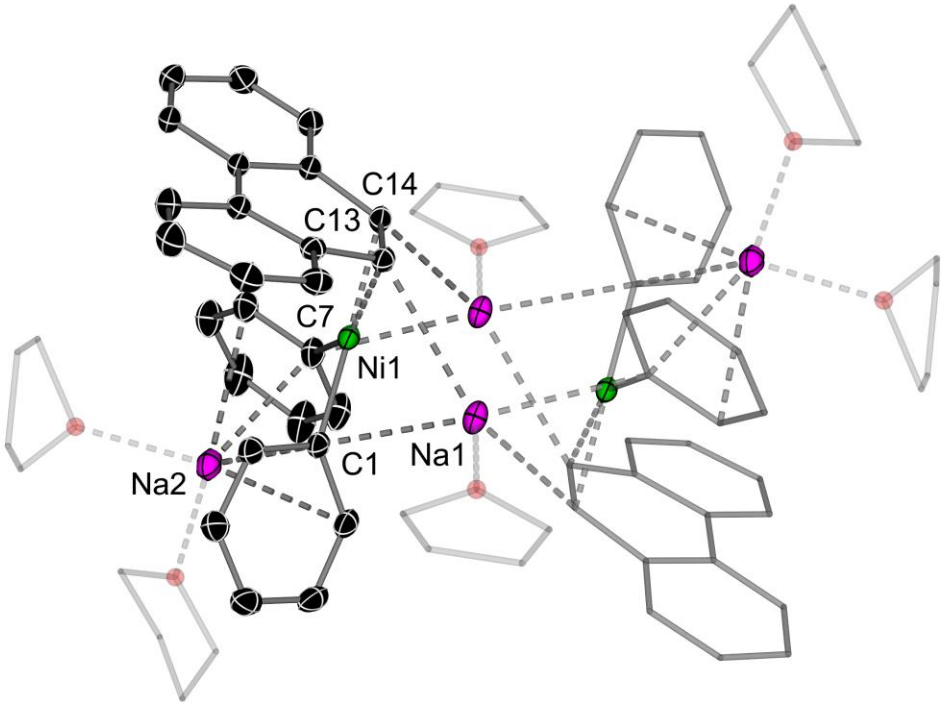


**Figure S15:** Solid-state structure of $[Na_2(THF)_3Ph_2Ni(\eta^2\text{-phenanthrene})]_2$ (**5Na**). Thermal ellipsoids shown at 30% probability. Hydrogen atoms and disordered components removed and coordinated THF shown as wireframes for clarity. Only one molecule of the asymmetric unit is displayed. Selected bond lengths [Å]: Ni1–C1 1.950(2); Ni1–C7 1.948(2); Ni1–C13 1.976(2); Ni1–C14 1.987(2); C13–C14 1.452(3).

**Solid-State Structure of $[K_2(THF)_1Ph_2Ni(\eta^2\text{-phenanthrene})]_\infty$ (5K)**

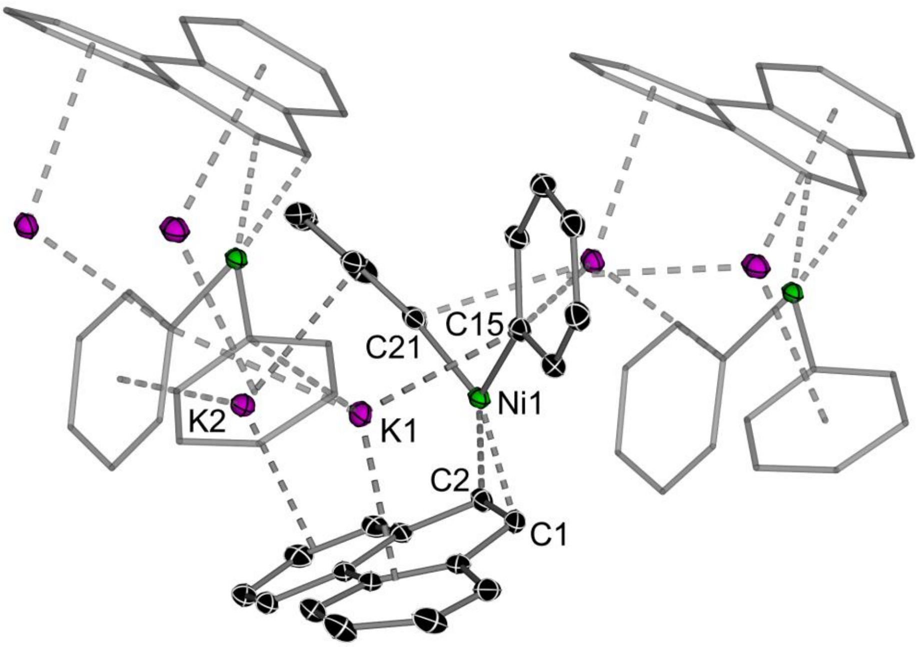


**Figure S16:** Solid-state structure of $[K_2(THF)_1Ph_2Ni(\eta^2\text{-phenanthrene})]_\infty$ (**5Na**). Thermal ellipsoids shown at 30% probability. Hydrogen atoms and disordered components removed and coordinated THF shown as wireframes for clarity. Selected bond lengths [Å]: Ni1–C15 1.976(3); Ni1–C21 1.946(3); Ni1–C1 2.013(3); Ni1–C2 1.971(3); C1–C2 1.447(4).

**Solid-State Structure of [Li(THF)$_2$Ph$_2$Ni($\eta^3$-perylene)][Li(THF)$_4$] (6Li)**

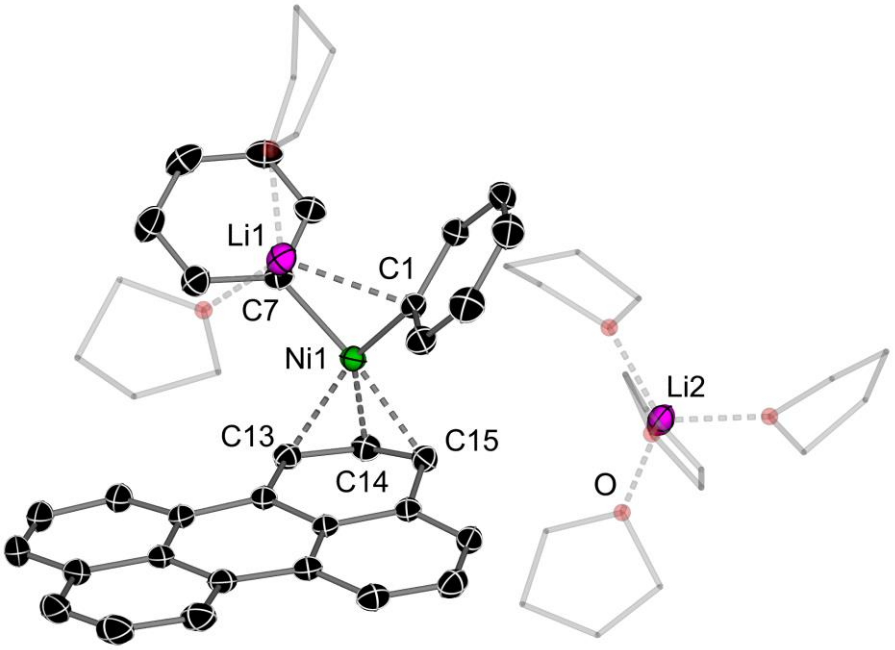


**Figure S17:** Solid-state structure of [Li(THF)$_2$Ph$_2$Ni($\eta^3$-perylene)][Li(THF)$_4$] (**6Li**). Thermal ellipsoids shown at 30% probability. Hydrogen atoms and disordered components removed and coordinated THF shown as wireframes for clarity. Selected bond lengths [Å]: Ni1–C1 1.944(2); Ni1–C7 1.954(2); Ni1–C13 2.171(2); Ni1–C14 1.977(2); Ni1–C15 2.088(2); C13–C14 1.401(3); C14–C15 1.413(3).

**Solid-State Structure of [perylene][K(DME)$_4$]**

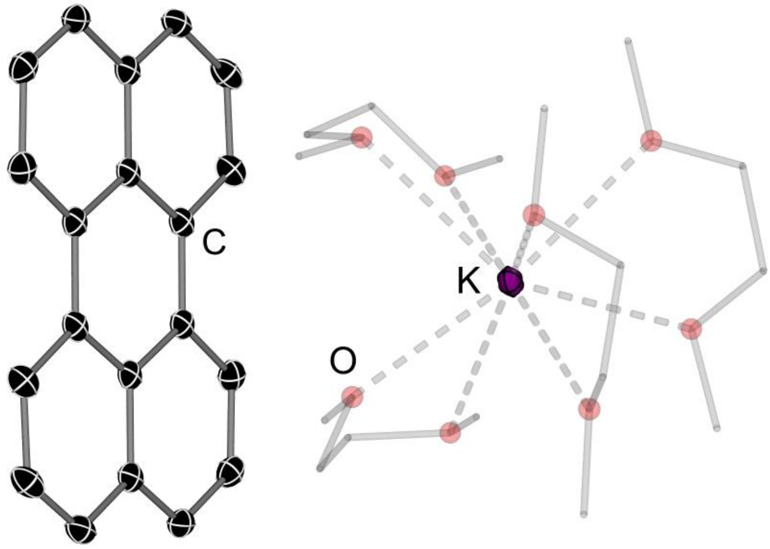


**Figure S18:** Solid-state structure of [perylene][K(DME)$_4$]. Thermal ellipsoids shown at 30% probability. Hydrogen atoms removed and coordinated DME shown as wireframes for clarity. During the preparation of this manuscript, an identical crystal structure of [perylene][K(DME)$_4$] was also reported by Balashova and co-workers.[12]

**Solid-State Structure of [Li(THF)$_2$Ph$_2$Ni($\eta^2$-coronene)][Li(THF)$_4$] (7Li)**

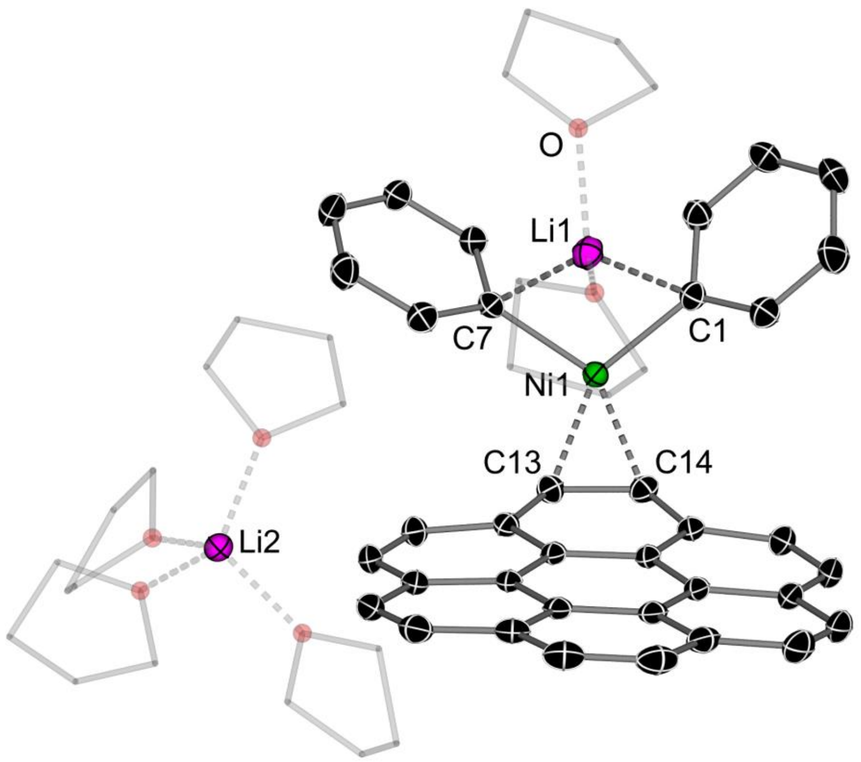


**Figure S19:** Solid-state structure of [Li(THF)$_2$Ph$_2$Ni($\eta^2$-coronene)][Li(THF)$_4$] (**7Li**). Thermal ellipsoids shown at 30% probability. Hydrogen atoms and disordered components removed and coordinated THF shown as wireframes for clarity. Selected bond lengths [Å]: Ni1–C1 1.960(2); Ni1–C7 1.957(2); Ni1–C13 1.983(2); Ni1–C14 2.005(2); C13–C14 1.460(2).

**Solid-State Structure of $K_2(DME)_4Ph_2Ni(\eta^2$-coronene) (7K)**

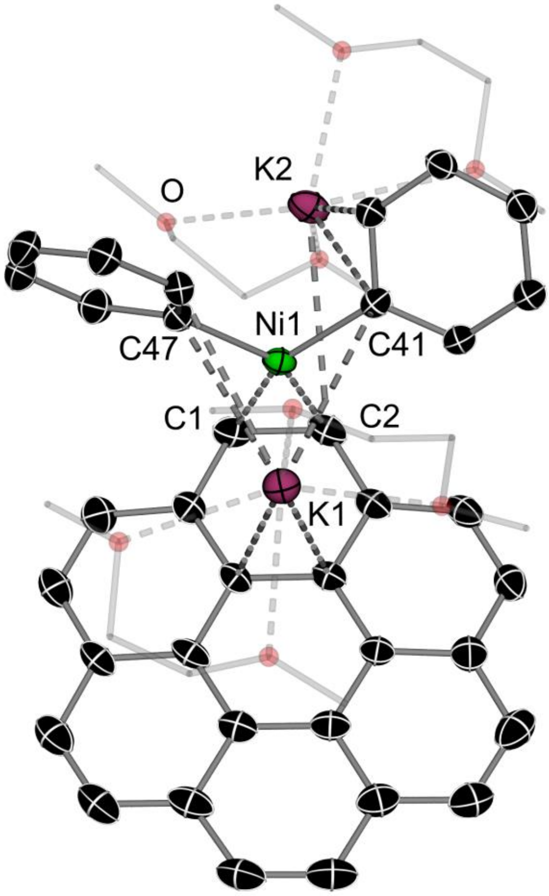


**Figure S20:** Solid-state structure of $K_2(DME)_4Ph_2Ni(\eta^2$-coronene) (**7K**). Thermal ellipsoids shown at 30% probability. Hydrogen atoms and disordered components removed and coordinated DME shown as wireframes for clarity. Only one molecule in the asymmetric unit is displayed. Selected bond lengths [Å]: Ni1–C41 1.937(6); Ni1–C47 1.936(6); Ni1–C1 1.986(6); Ni1–C2 1.989(7); C1–C2 1.48(1).

**Solid-State Structure of $[Li_2(THF)_3Ph_2Ni(\eta^2$-$Ph_2CO)]_2$ (8Li)**

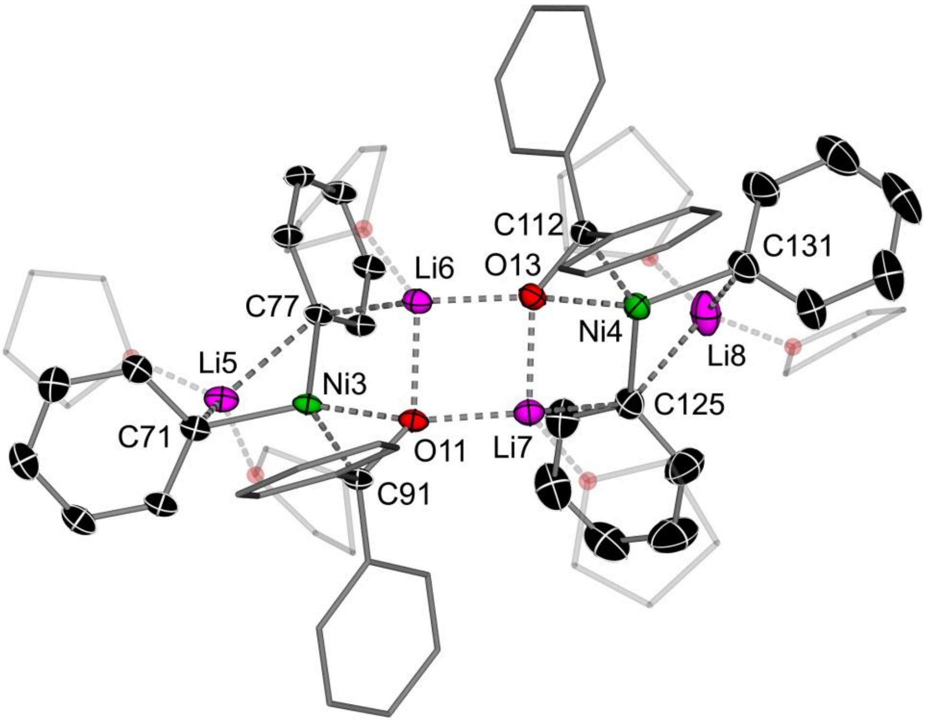

**Figure S21:** Solid-state structure of [$Li_2(THF)_3Ph_2Ni(\eta^2$-$Ph_2CO)]_2$ (**8Li**). Thermal ellipsoids shown at 30% probability. Hydrogen atoms and disordered components removed and coordinated THF and phenyl-substituents of $Ph_2CO$ shown as wireframes for clarity. Only one molecule in the asymmetric unit is displayed. Selected bond lengths [Å]: Ni3–C71 1.897(2); Ni3–C77 1.970(2); Ni3–C91 1.919(2); Ni3–O11 1.928(1); C91–O11 1.391(2); Ni4–C125 1.956(2); Ni4–C131 1.892(2); Ni4–C112 1.916(2); Ni4–O13 1.924(1); C112–O13 1.389(2).

### Solid-State Structure of [$K_2(DME)_3Ph_2Ni(\eta^2$-$Ph_2CO)]_2$ (8K)

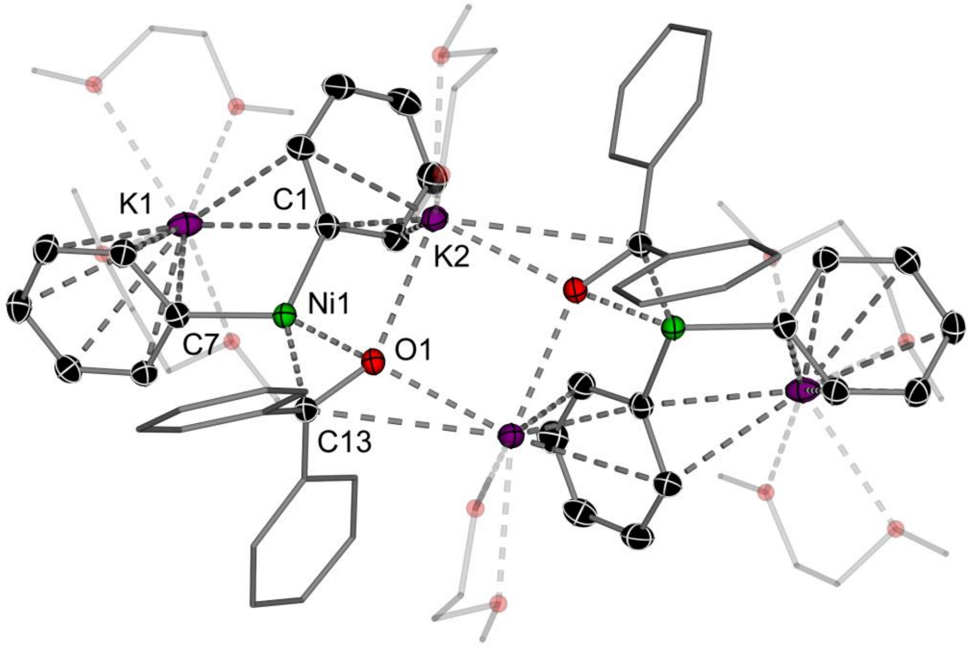


**Figure S22:** Solid-state structure of [$K_2(DME)_3Ph_2Ni(\eta^2$-$Ph_2CO)]_2$ (**8K**). Thermal ellipsoids shown at 30% probability. Hydrogen atoms and disordered components removed and coordinated DME and phenyl-substituents of $Ph_2CO$ shown as wireframes for clarity. Selected bond lengths [Å]: Ni1–C1 1.929(1); Ni1–C7 1.880(1); Ni1–C13 1.929(2); Ni1–O1 1.928(1); C13–O1 1.375(2).

**Solid-State Structure of $Li_2(THF)_5Ph_2Ni(\eta^2\text{-}PhCH{=}NPh)$ (9Li)**

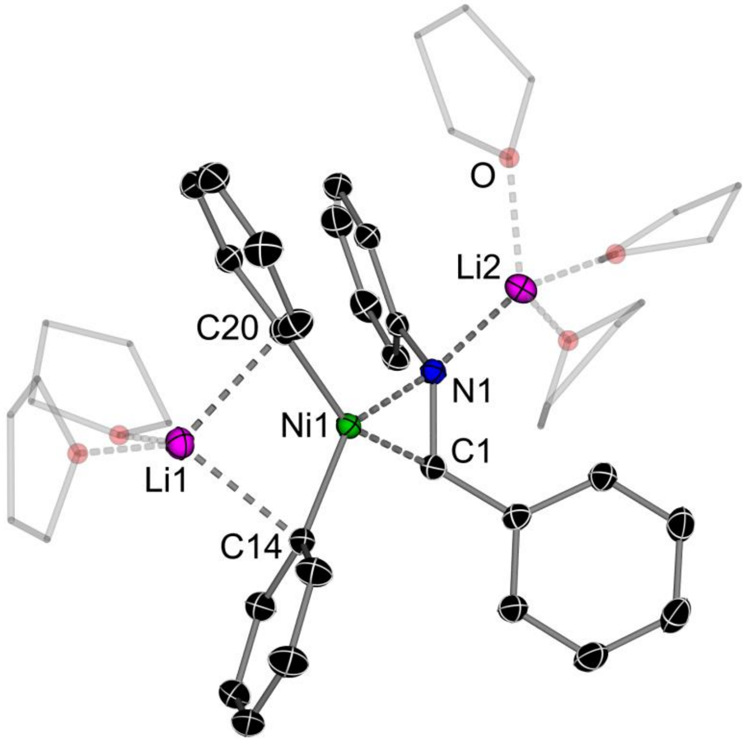


**Figure S23:** Solid-state structure of $Li_2(THF)_5Ph_2Ni(\eta^2\text{-}PhCH{=}NPh)$ (**9Li**). Thermal ellipsoids shown at 30% probability. Hydrogen atoms removed and coordinated THF shown as wireframes for clarity. Selected bond lengths [Å]: Ni1–C14 1.929(1); Ni1–C20 1.966(1); Ni1–C1 1.925(1); Ni1–N1 1.956(1); C1–N1 1.419(2).

**Solid-State Structure of $[Li_2(THF)_3Ph_2Ni(\eta^2\text{-}Ph_2CHCN)]_2$ (10Li)**

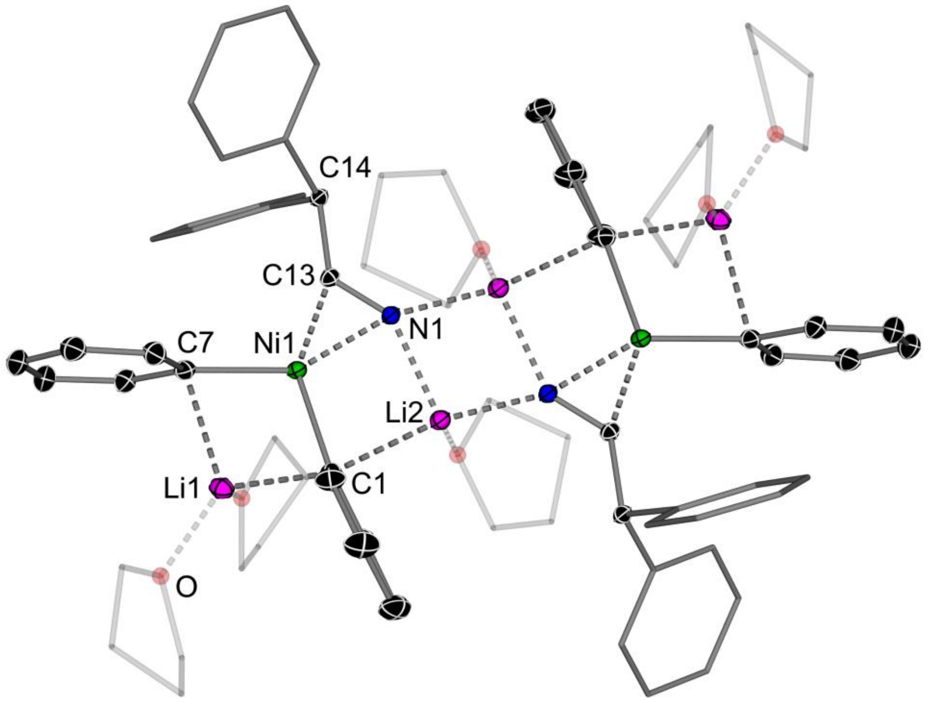


**Figure S24:** Solid-state structure of $[Li_2(THF)_3Ph_2Ni(\eta^2\text{-}Ph_2CHCN)]_2$ (**10Li**). Thermal ellipsoids shown at 30% probability. Hydrogen atoms and disordered components removed and coordinated THF and phenyl-substituents of $Ph_2CHCN$ shown as wireframes for clarity. Selected bond lengths [Å]: Ni1–C1 1.986(1); Ni1–C7 1.932(1); Ni1–C13 1.839(1); Ni1N1 1.954(2); C13–N1 1.242(2).

**Solid-State Structure of $Li(THF)_3(PPh_3)_2(CO)_3Ni_2(\mu\text{-}PPh_2)$ (11Li)**

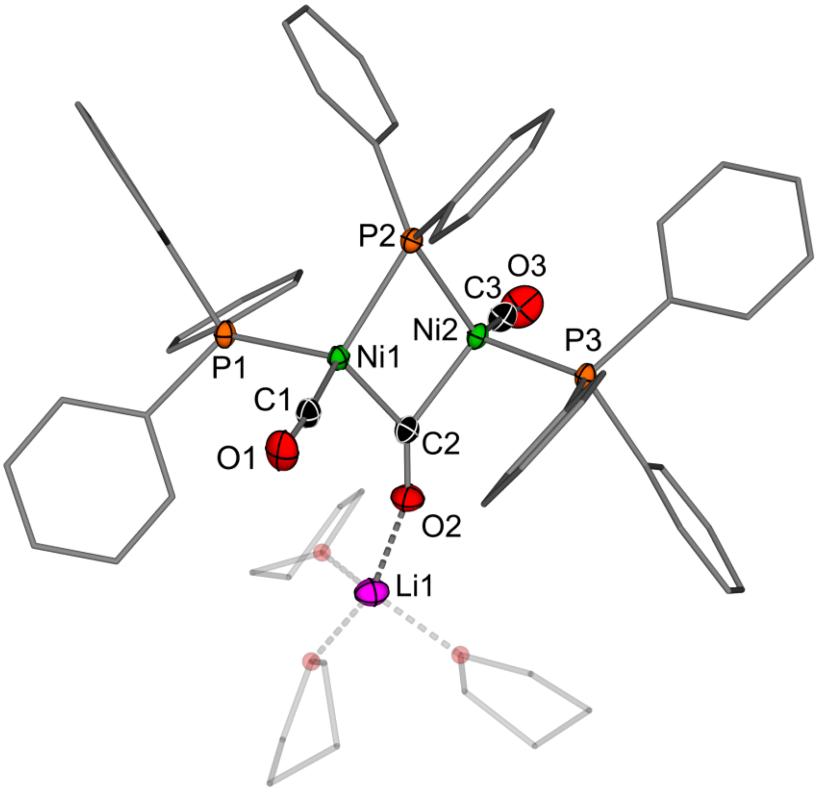


**Figure S25:** Solid-state structure of $Li(THF)_3(PPh_3)_2(CO)_3Ni_2(\mu\text{-}PPh_2)$ (**11Li**). Thermal ellipsoids shown at 30% probability. Hydrogen atoms and disordered components removed and coordinated THF and phenyl-substituents shown as wireframes for clarity. Selected bond lengths [Å]: Ni1–P1 2.1928(6); Ni1–C1 1.757(2); C1–O1 1.150(3); Ni1–P2 2.2245(4); Ni1–C2 1.902(2); C2–O2 1.187(2); Ni1–Ni2 2.4845(4); Ni2–P2 2.2257(5); Ni2–C2 1.902(2); Ni2–C3 1.746(2); C3–O3 1.154(3); Ni2–P3 2.1967(6).

## NMR Spectra of Reported Compounds

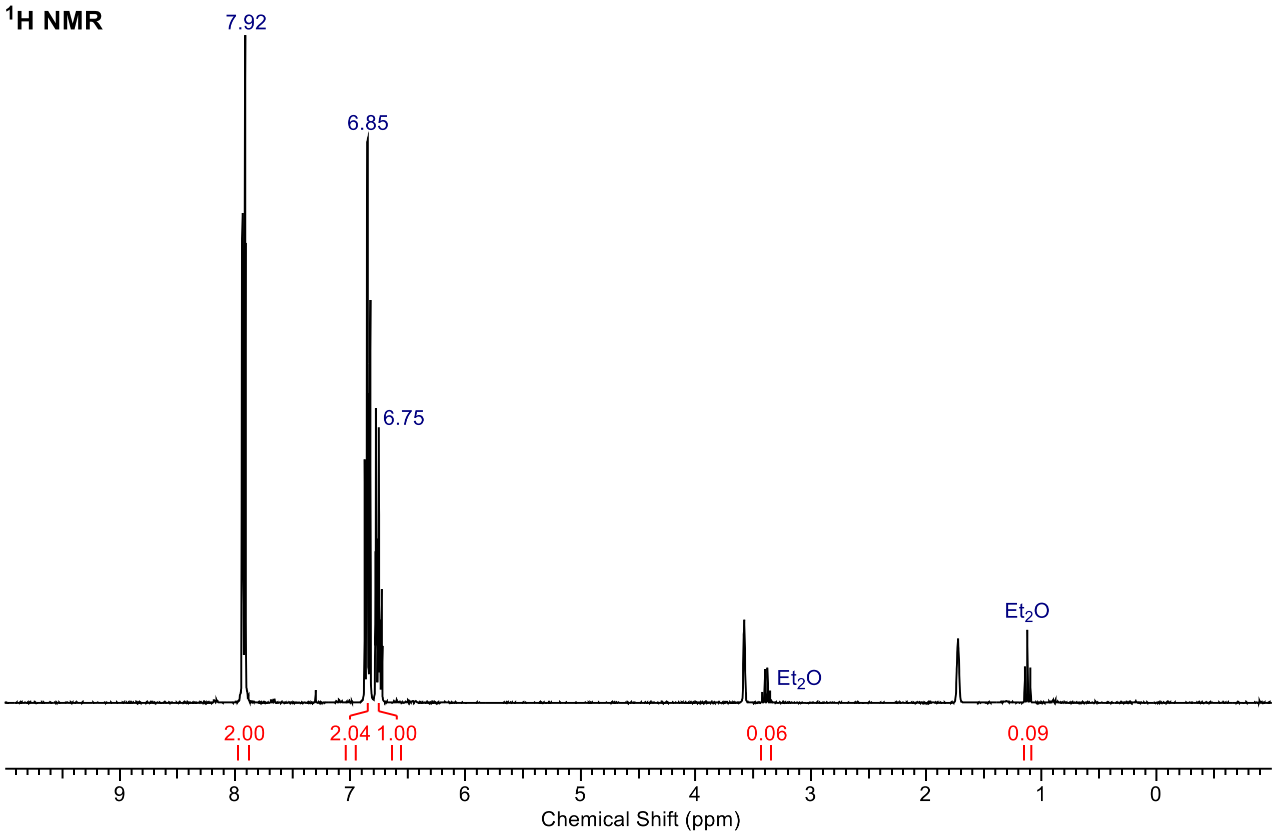


**Spectra S1:** $^{1}H$ NMR spectrum of $PhLi(Et_2O)_{0.02}$ in THF-$d_8$.

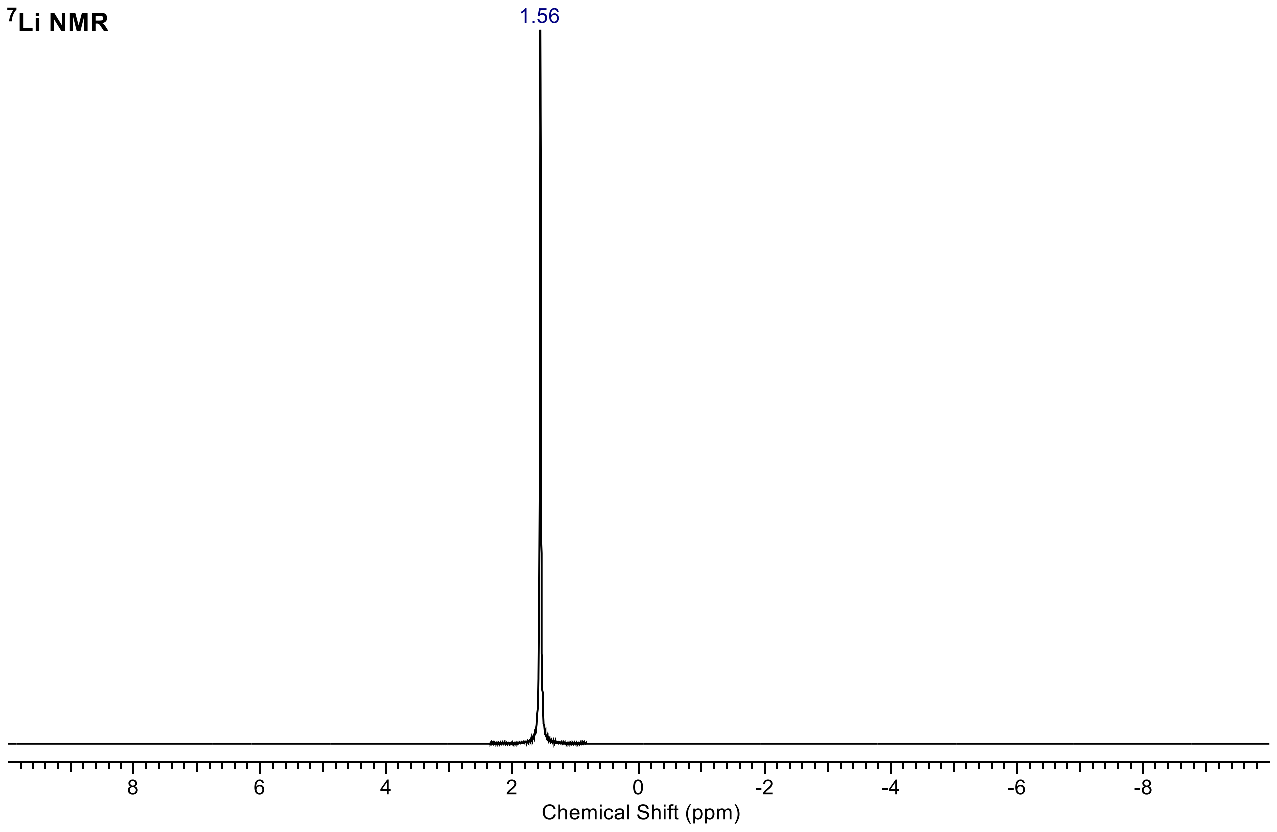


**Spectra S2:** $^{7}Li$ NMR spectrum of $PhLi(Et_2O)_{0.02}$ in THF-$d_8$.

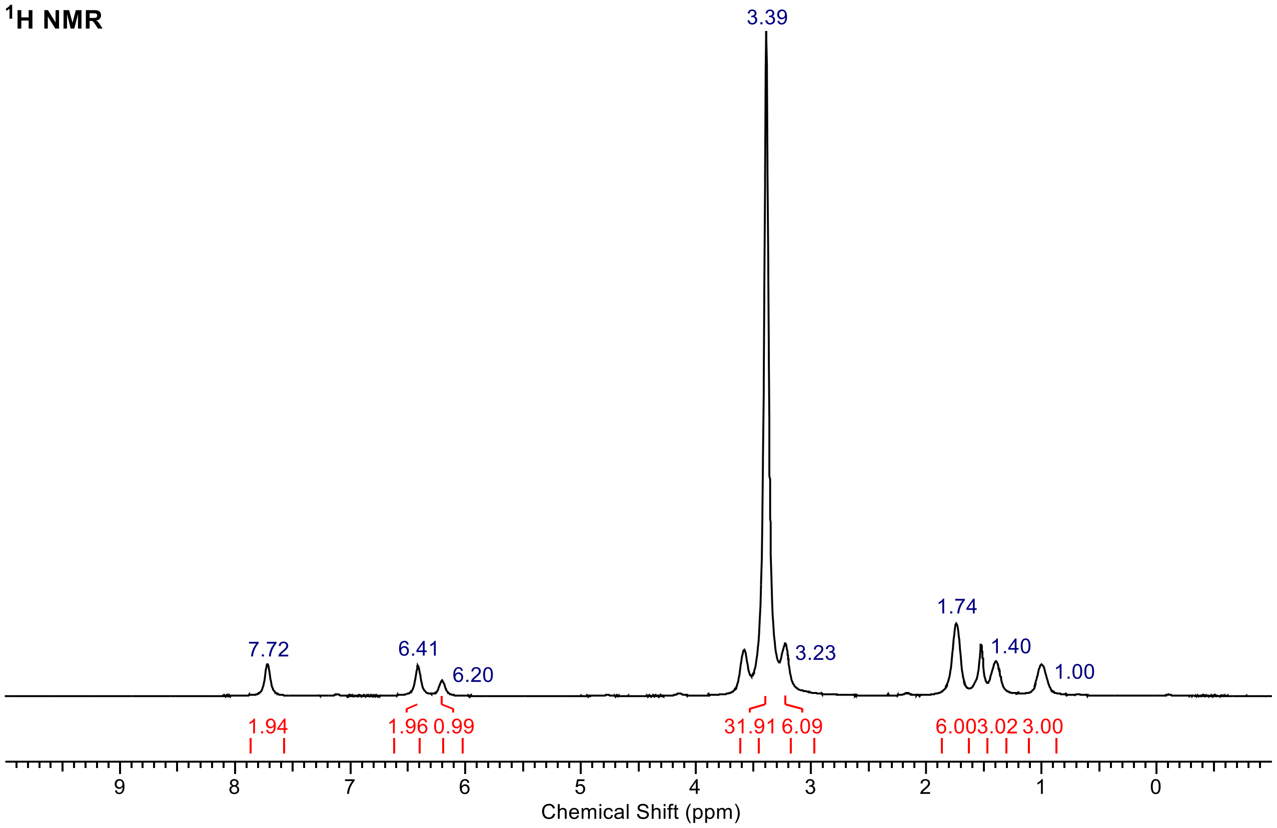


**Spectra S3:** $^{1}H$ NMR spectrum of [(*ttt*-CDT)NiPh][Li(12-crown-4)$_2$] (**1**) in THF-$d_8$ at -20 °C.

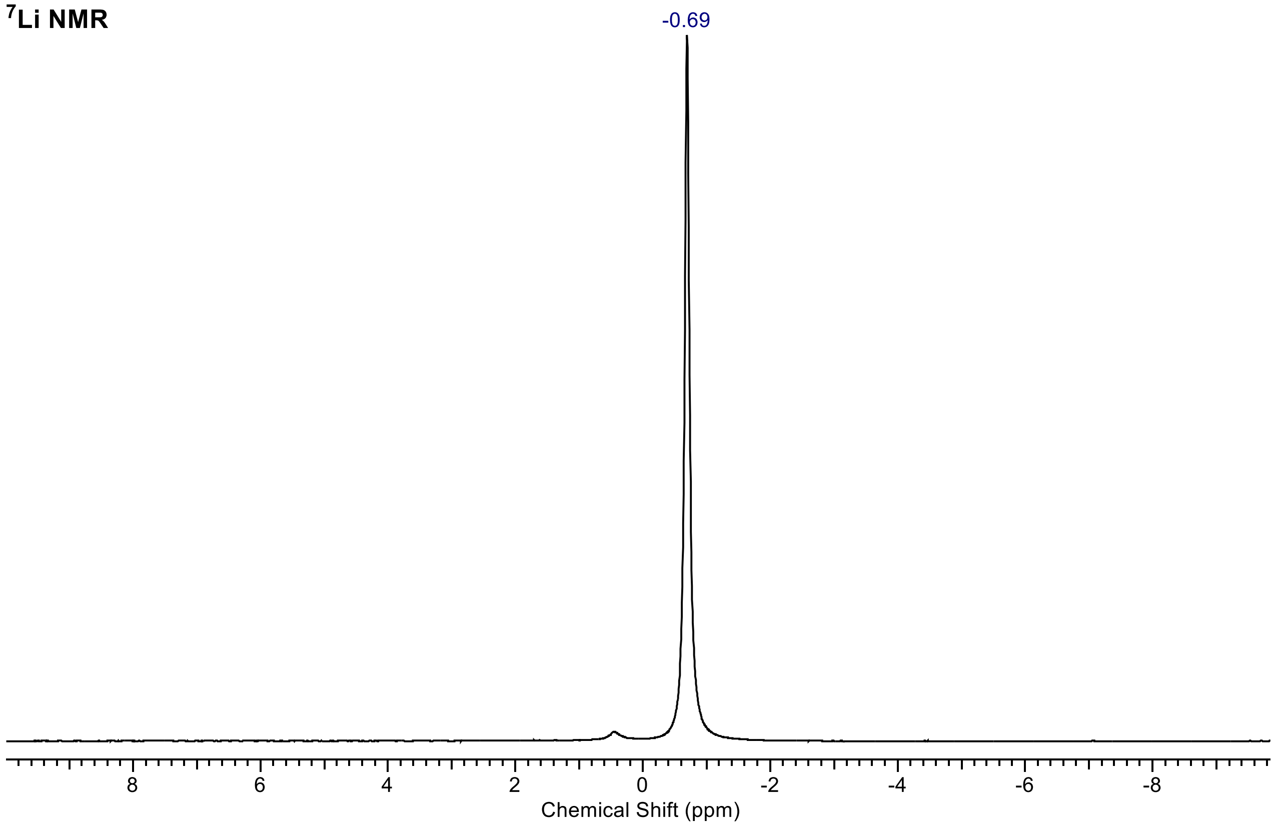


**Spectra S4:** $^{7}Li$ NMR spectrum of [(*ttt*-CDT)NiPh][Li(12-crown-4)$_2$] (**1**) in THF-$d_8$ at -20 °C.

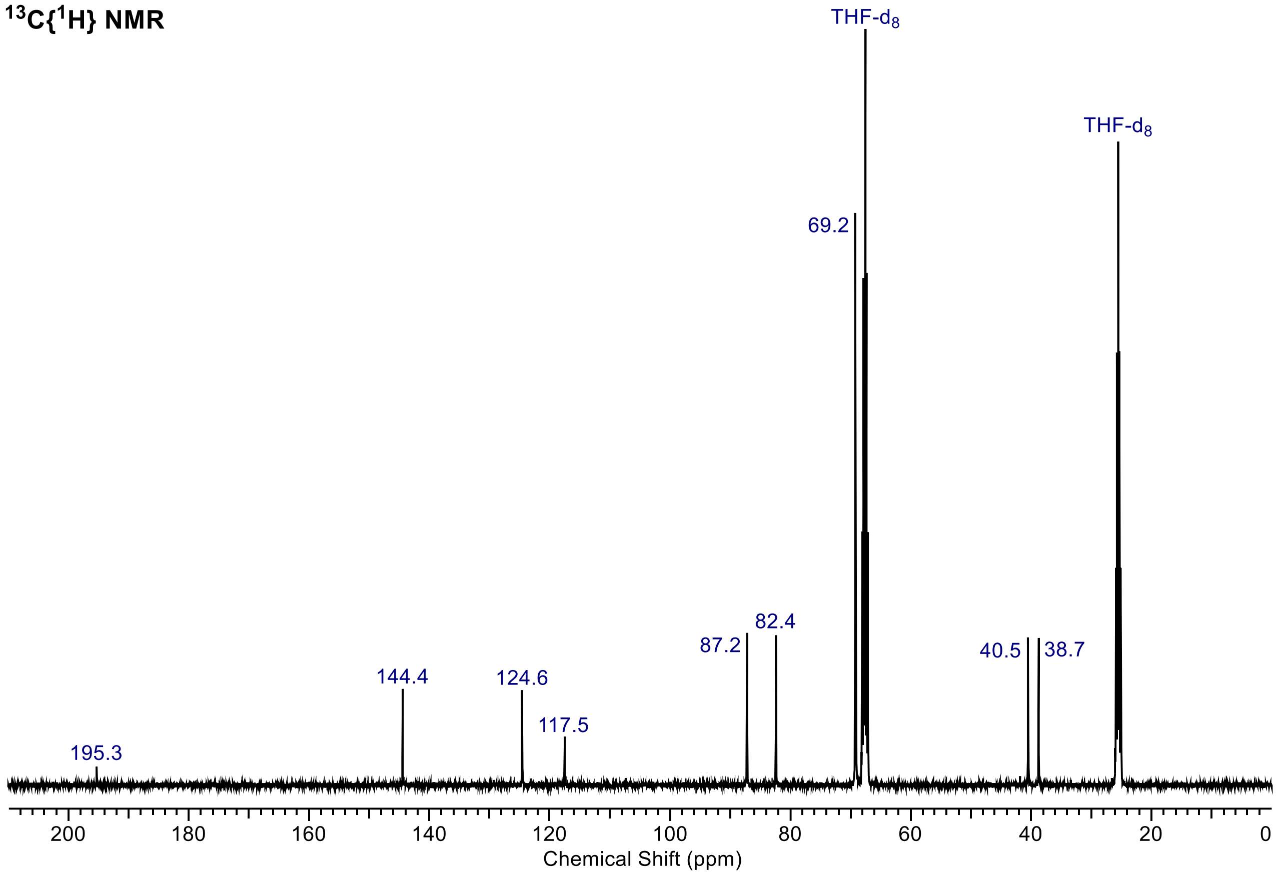


**Spectra S5:** $^{13}C\{^{1}H\}$ NMR spectrum of [(*ttt*-CDT)NiPh][Li(12-crown-4)$_2$] (**1**) in THF-d$_8$ at -20 °C.

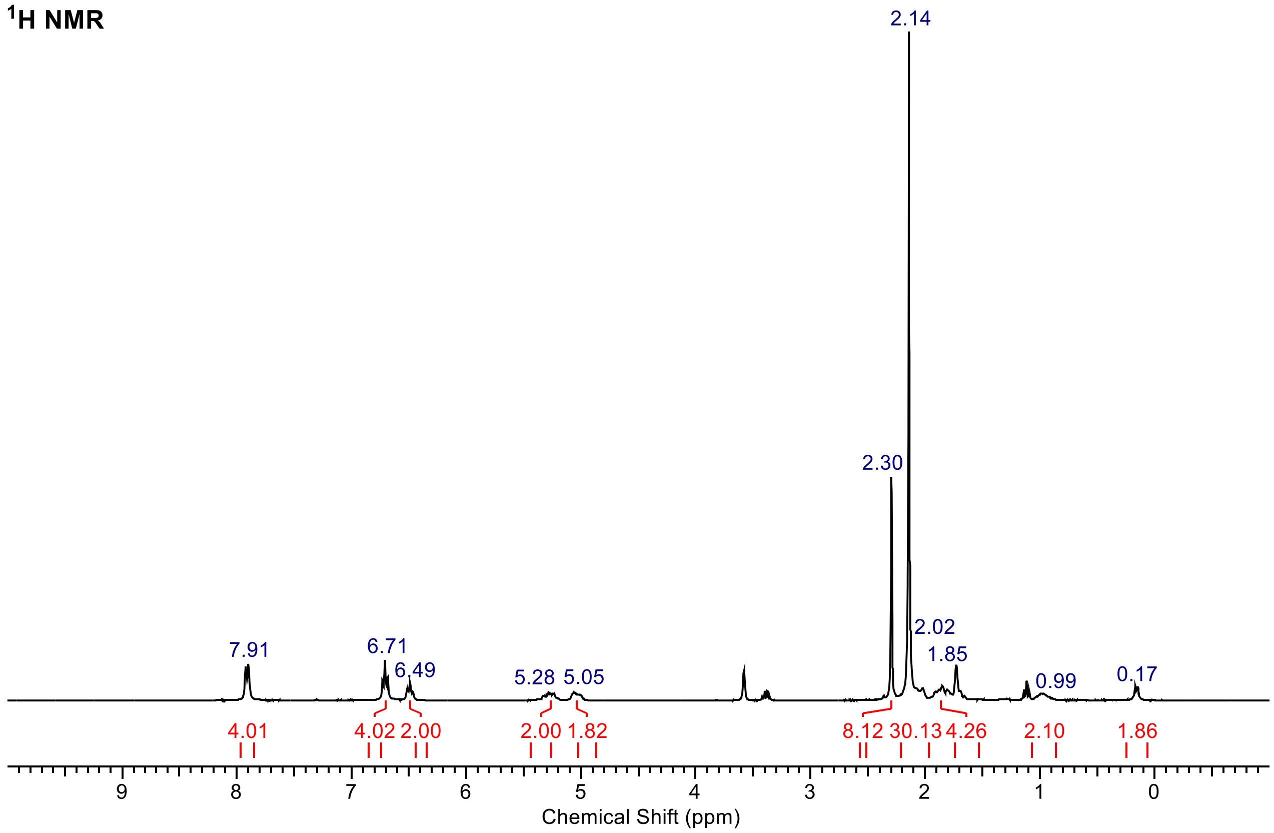


**Spectra S6:** $^{1}H$ NMR spectrum of $Li_2(TMEDA)_2Ph_2Ni(\eta^2\text{-CDT})$ (**2**) in THF-d$_8$.

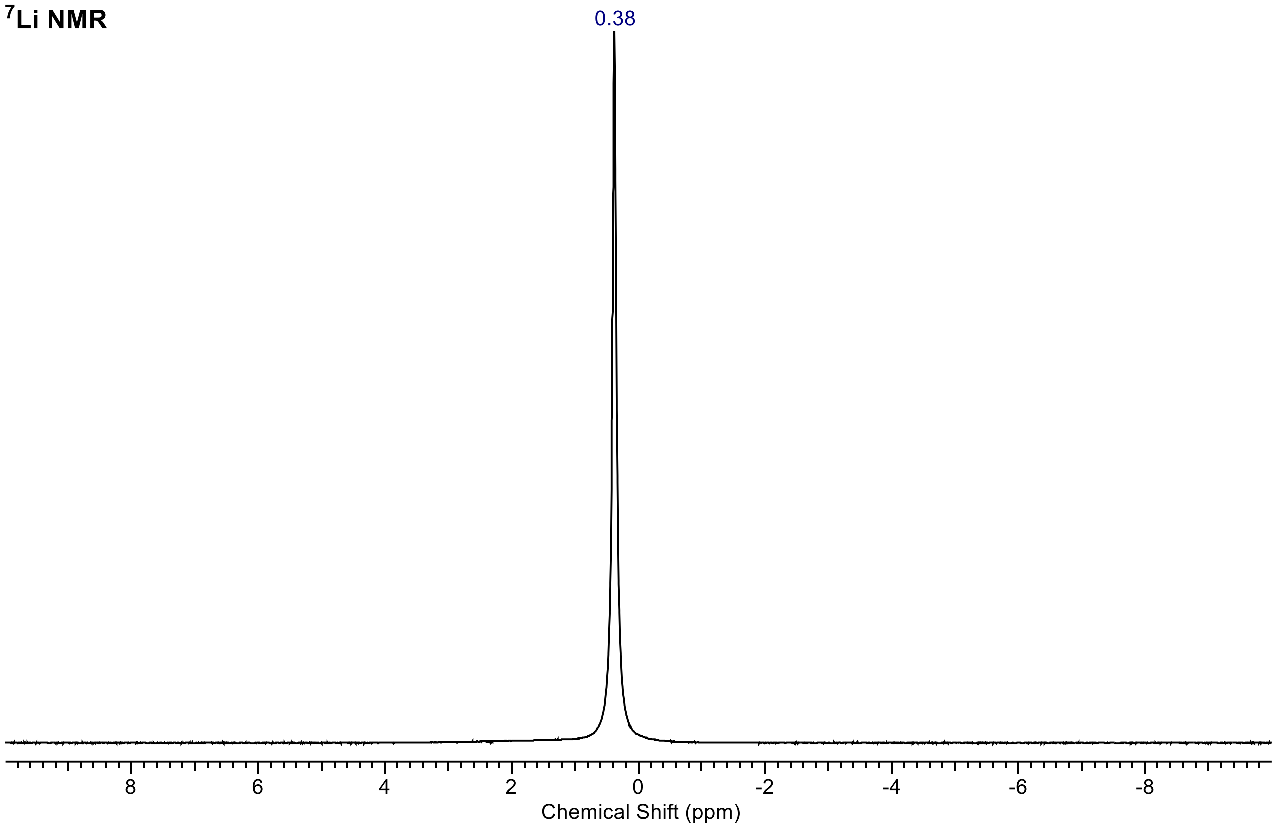


**Spectra S7:** $^{7}$Li NMR spectrum of $Li_2(TMEDA)_2Ph_2Ni(\eta^2$-CDT) (**2**) in THF-$d_8$.

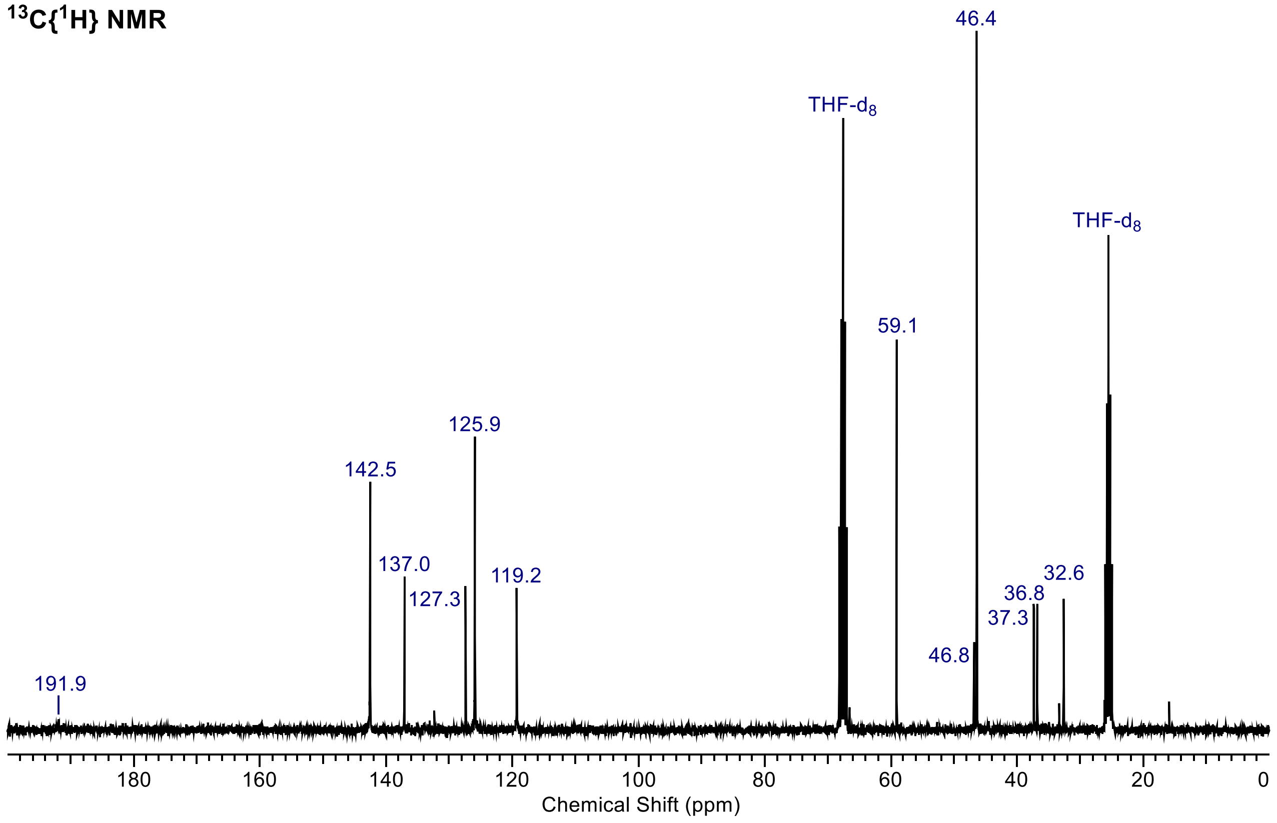


**Spectra S8:** $^{13}$C{$^{1}$H} NMR spectrum of $Li_2(TMEDA)_2Ph_2Ni(\eta^2$-CDT) (**2**) in THF-$d_8$.

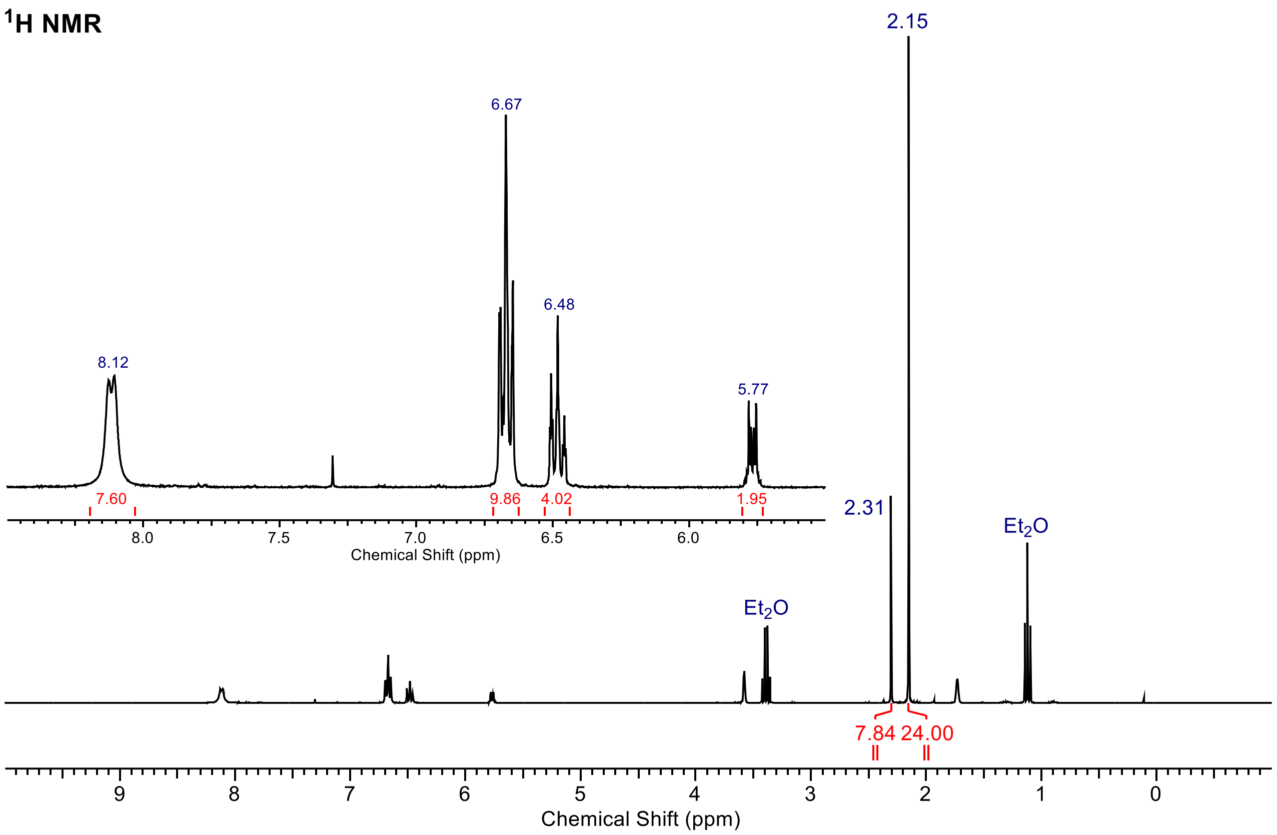


**Spectra S9:** $^{1}$H NMR spectrum of $Li_4(TMEDA)_2(Et_2O)_2Ph_4Ni_2(\mu;\eta^2;\eta^2\text{-}C_6H_4)$ (**3**) in THF-$d_8$.

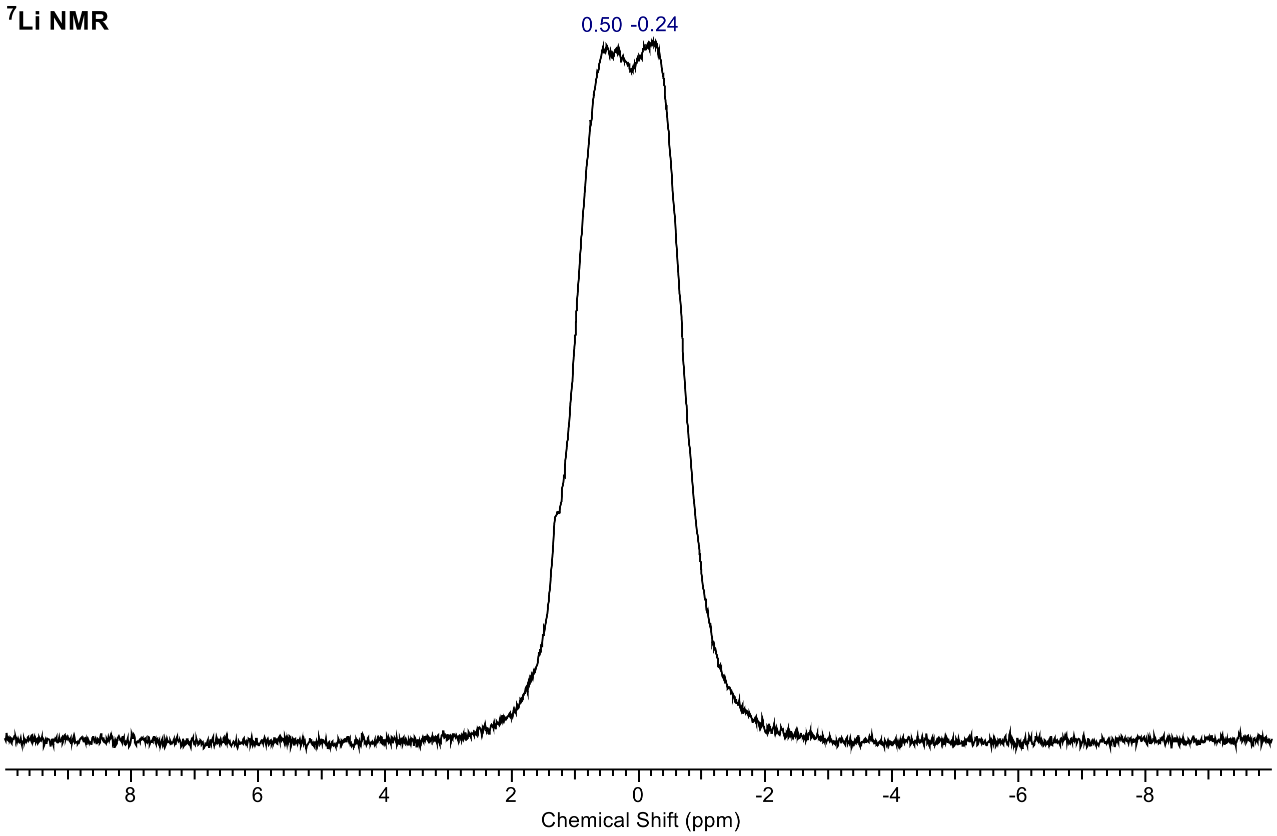


**Spectra S10:** $^{7}$Li NMR spectrum of $Li_4(TMEDA)_2(Et_2O)_2Ph_4Ni_2(\mu;\eta^2;\eta^2\text{-}C_6H_4)$ (**3**) in THF-$d_8$.

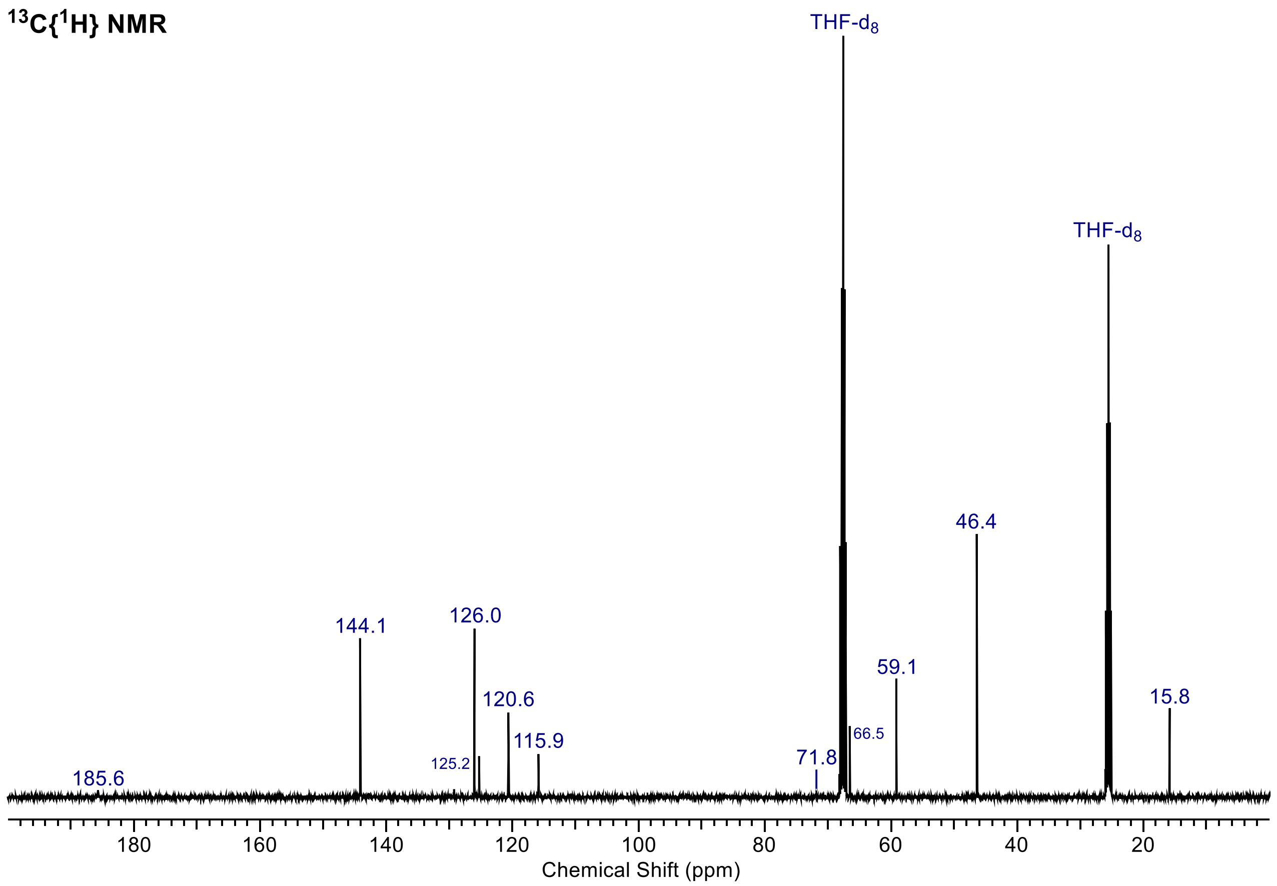


**Spectra S11:** $^{13}C\{^1H\}$ NMR spectrum of $Li_4(TMEDA)_2(Et_2O)_2Ph_4Ni_2(\mu;\eta^2;\eta^2\text{-}C_6H_4)$ (**3**) in THF-$d_8$.

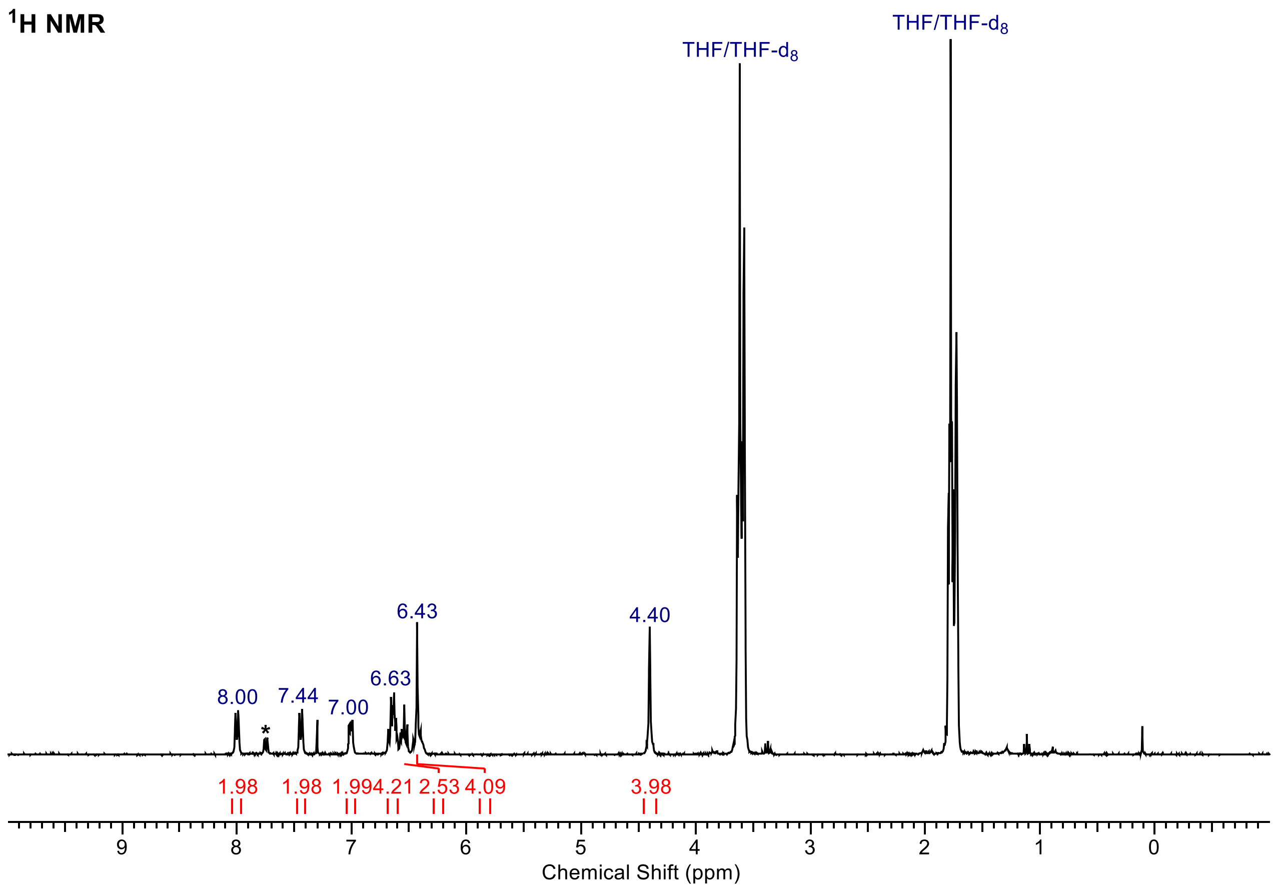


**Spectra S12:** $^1H$ NMR spectrum of $Li_2(THF)_4Ph_2Ni(\eta^2\text{-anthracene})$ (**4Li**) in THF-$d_8$. * Unidentified impurity.

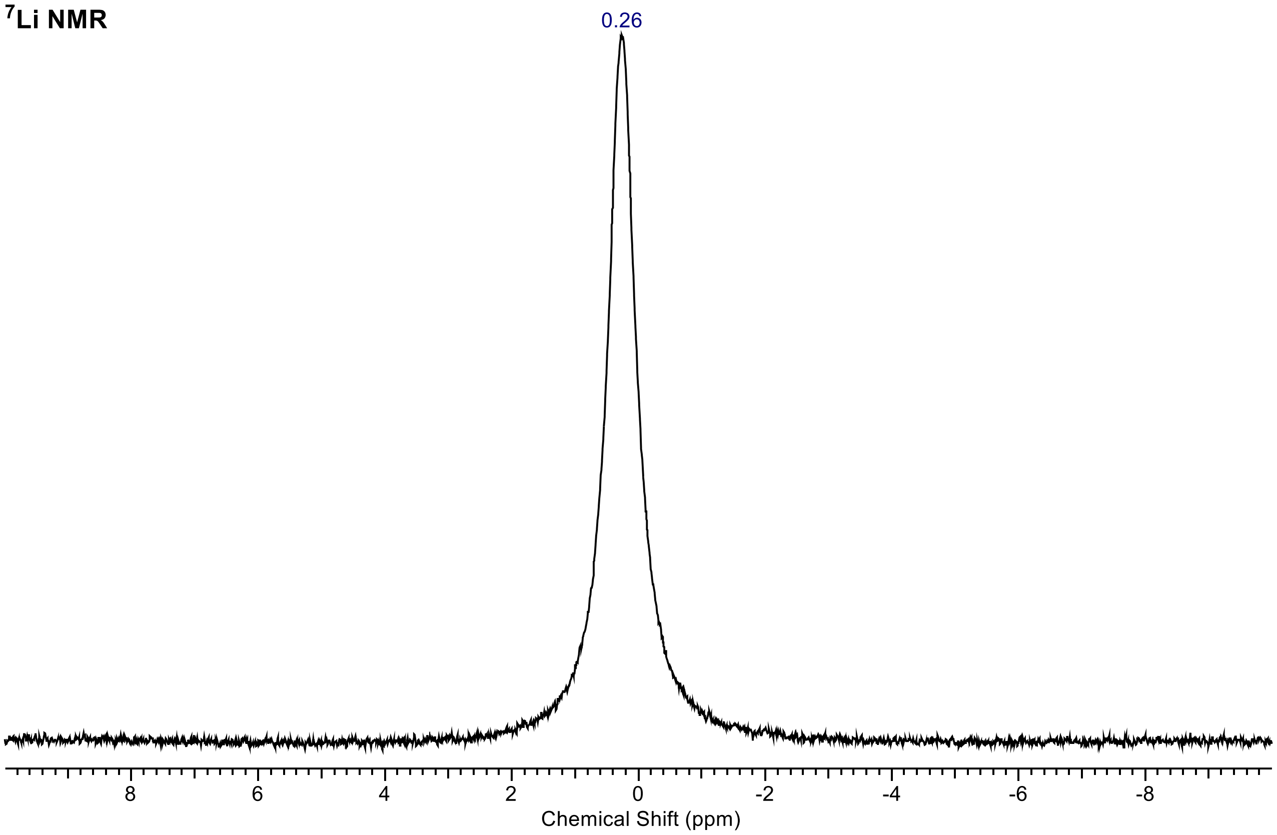


**Spectra S13:** $^{7}Li$ NMR spectrum of $Li_2(THF)_4Ph_2Ni(\eta^2$-anthracene) (**4Li**) in THF-$d_8$.

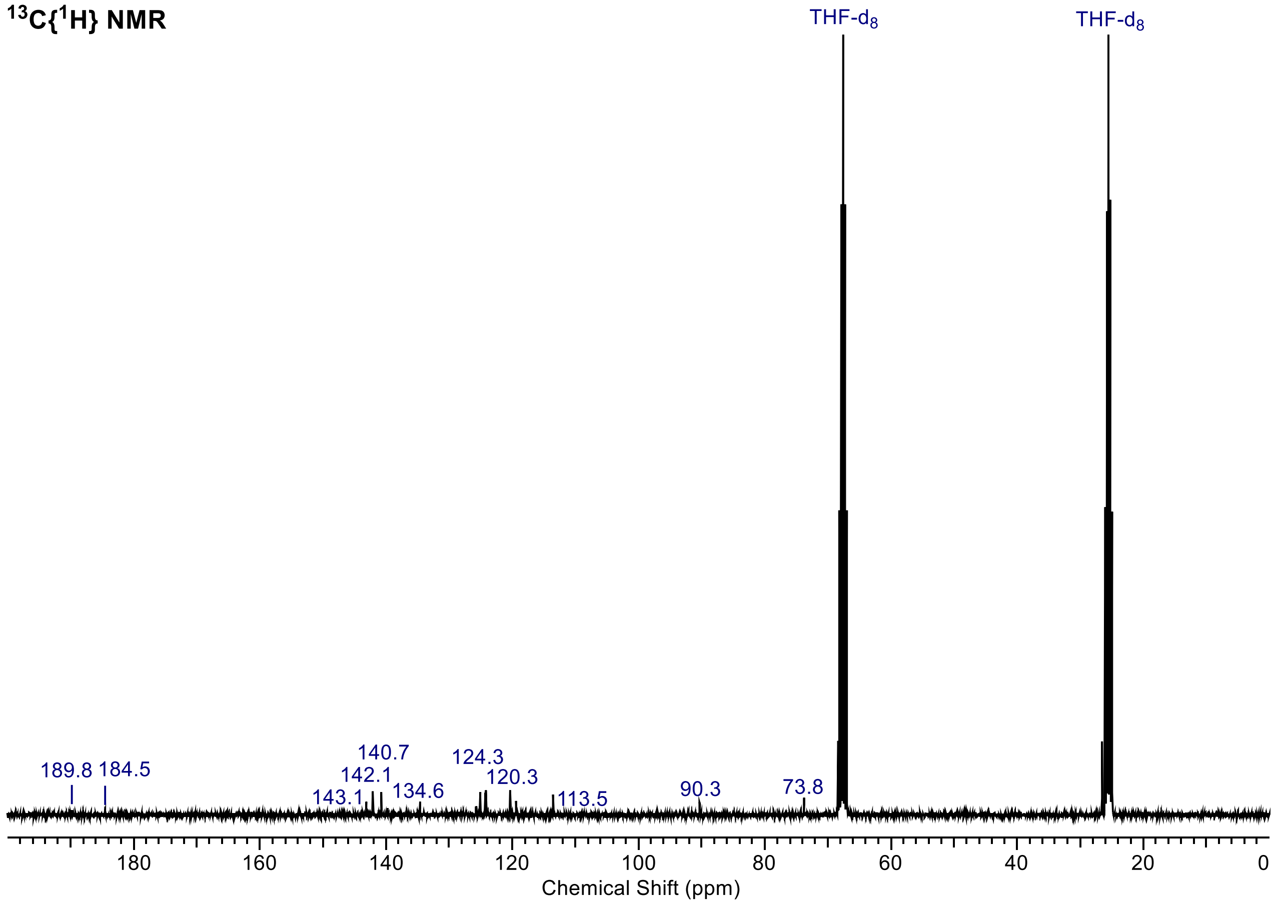


**Spectra S14:** $^{13}C\{^{1}H\}$ NMR spectrum of $Li_2(THF)_4Ph_2Ni(\eta^2$-anthracene) (**4Li**) in THF-$d_8$.

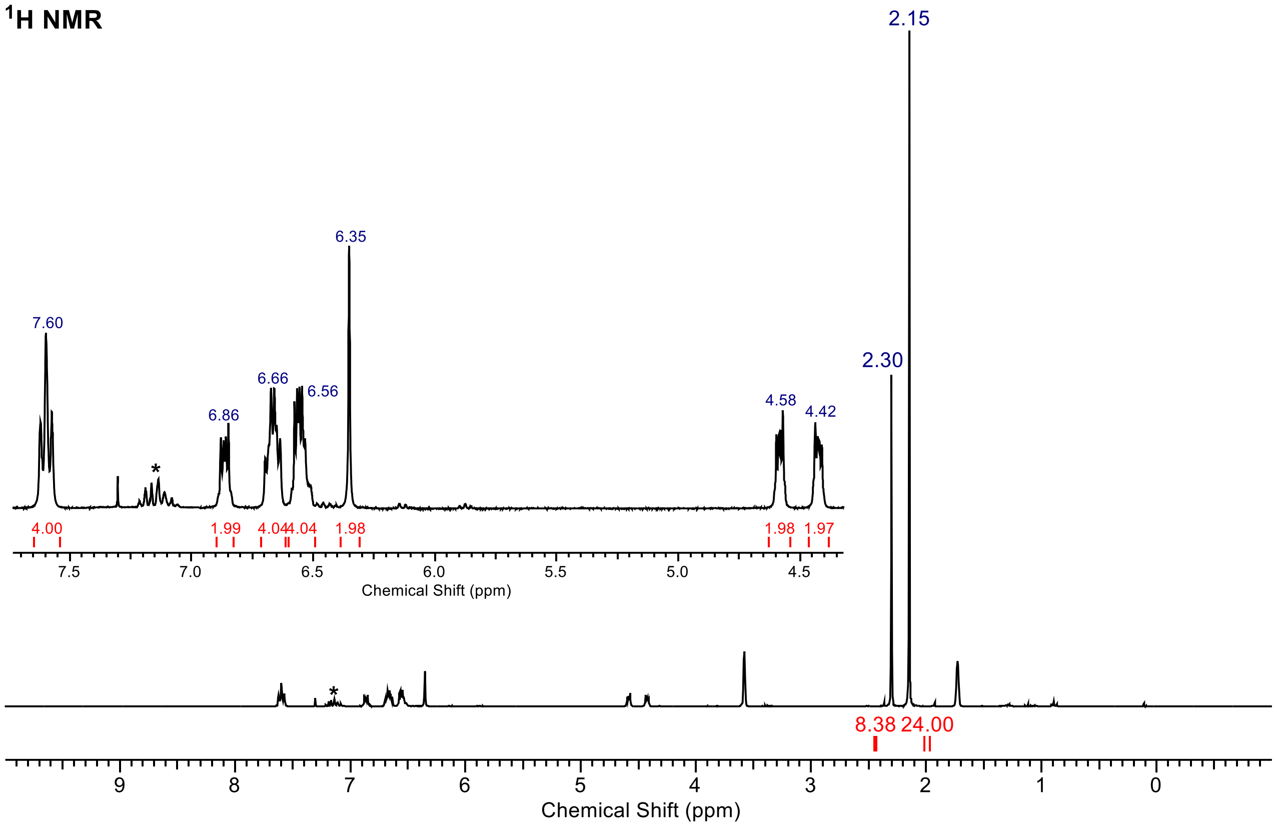


**Spectra S15:** $^1$H NMR spectrum of $Na_2(TMEDA)_2Ph_2Ni(\eta^2$-anthracene) (**4Na**) in THF-$d_8$. * Unidentified impurity.

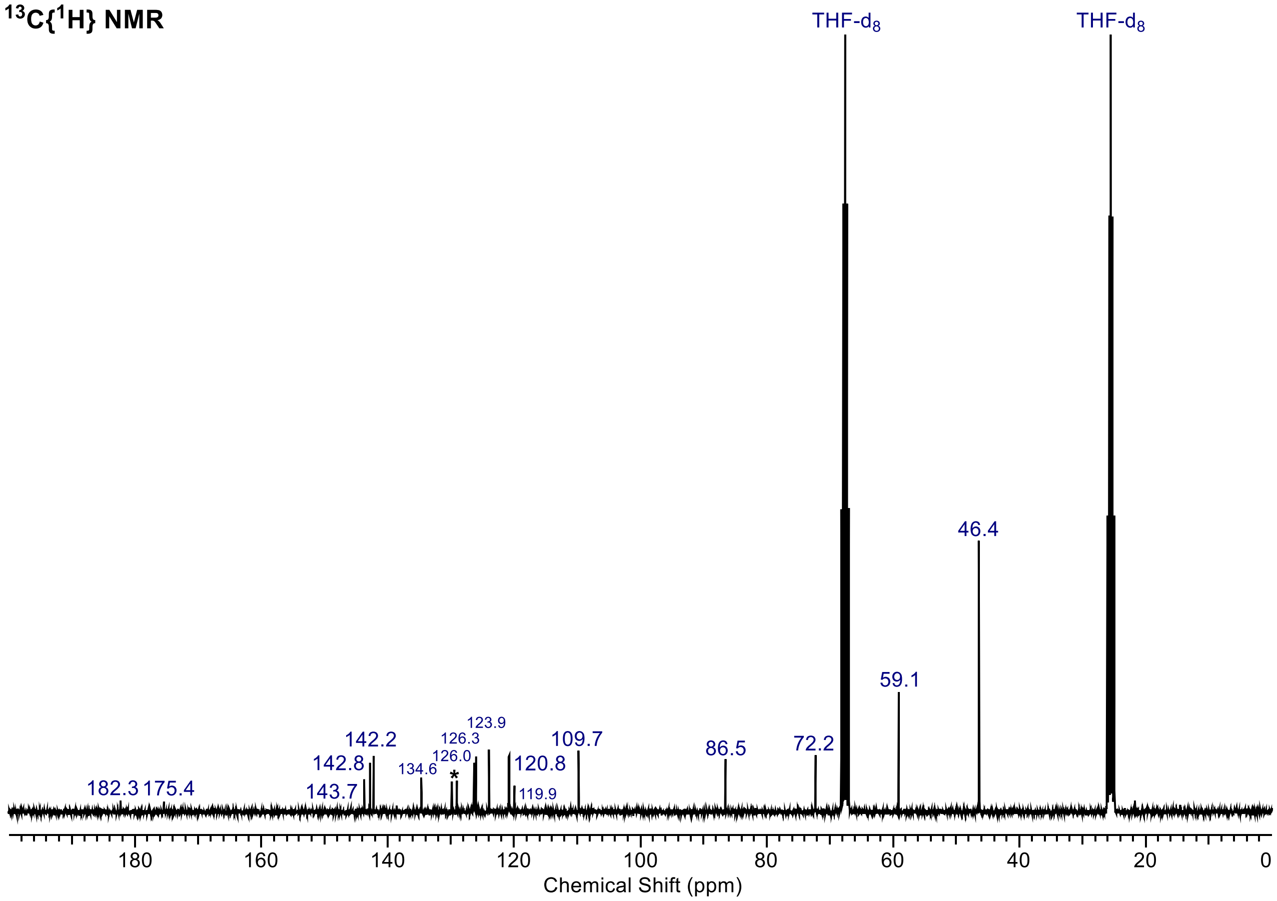


**Spectra S16:** $^{13}C\{^1H\}$ NMR spectrum of $Na_2(TMEDA)_2Ph_2Ni(\eta^2$-anthracene) (**4Na**) in THF-$d_8$. * $C_6H_6$.

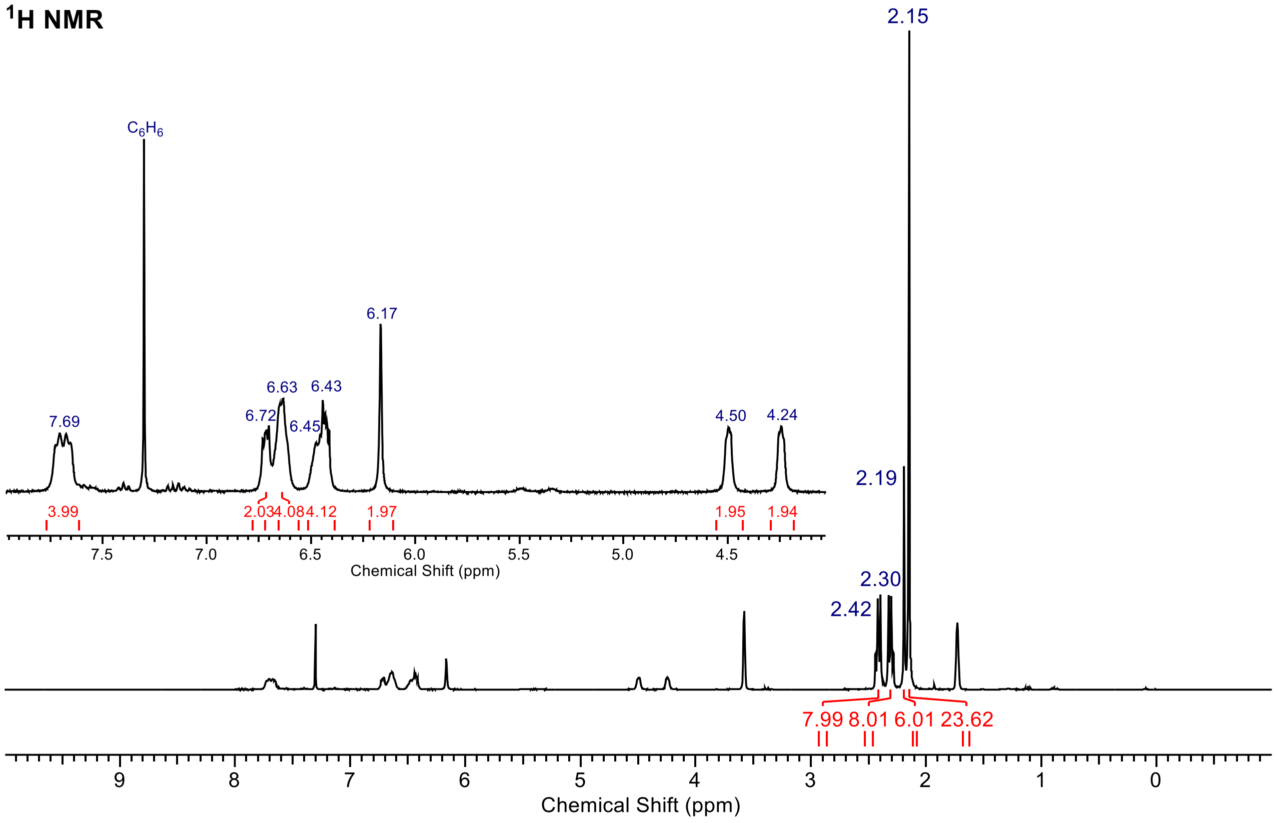


**Spectra S17:** $^1$H NMR spectrum of $K_2(PMDETA)_2Ph_2Ni(\eta^2$-anthracene) (**4K**) in THF-$d_8$.

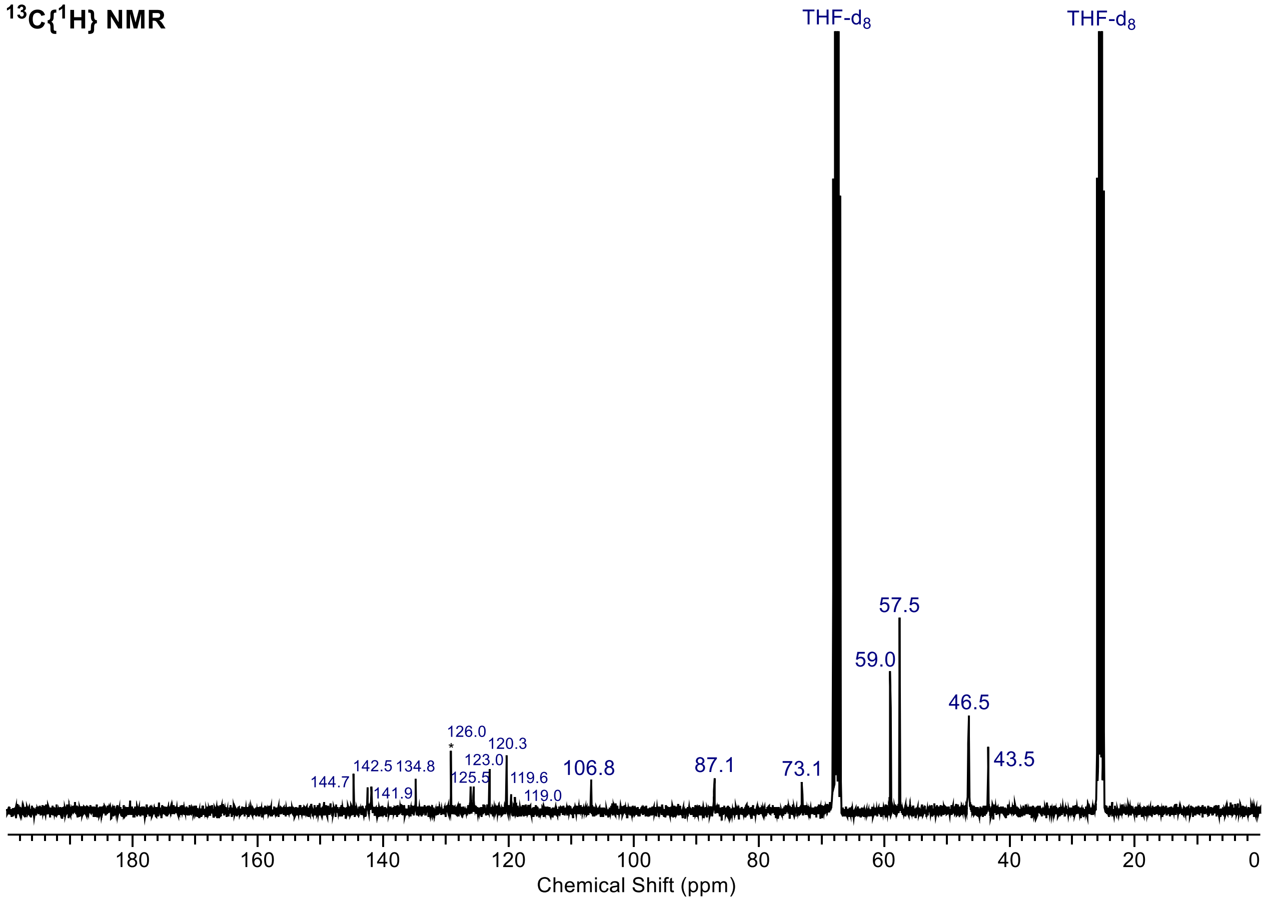


**Spectra S18:** $^{13}$C{$^1$H} NMR spectrum of $K_2(PMDETA)_2Ph_2Ni(\eta^2$-anthracene) (**4K**) in THF-$d_8$.

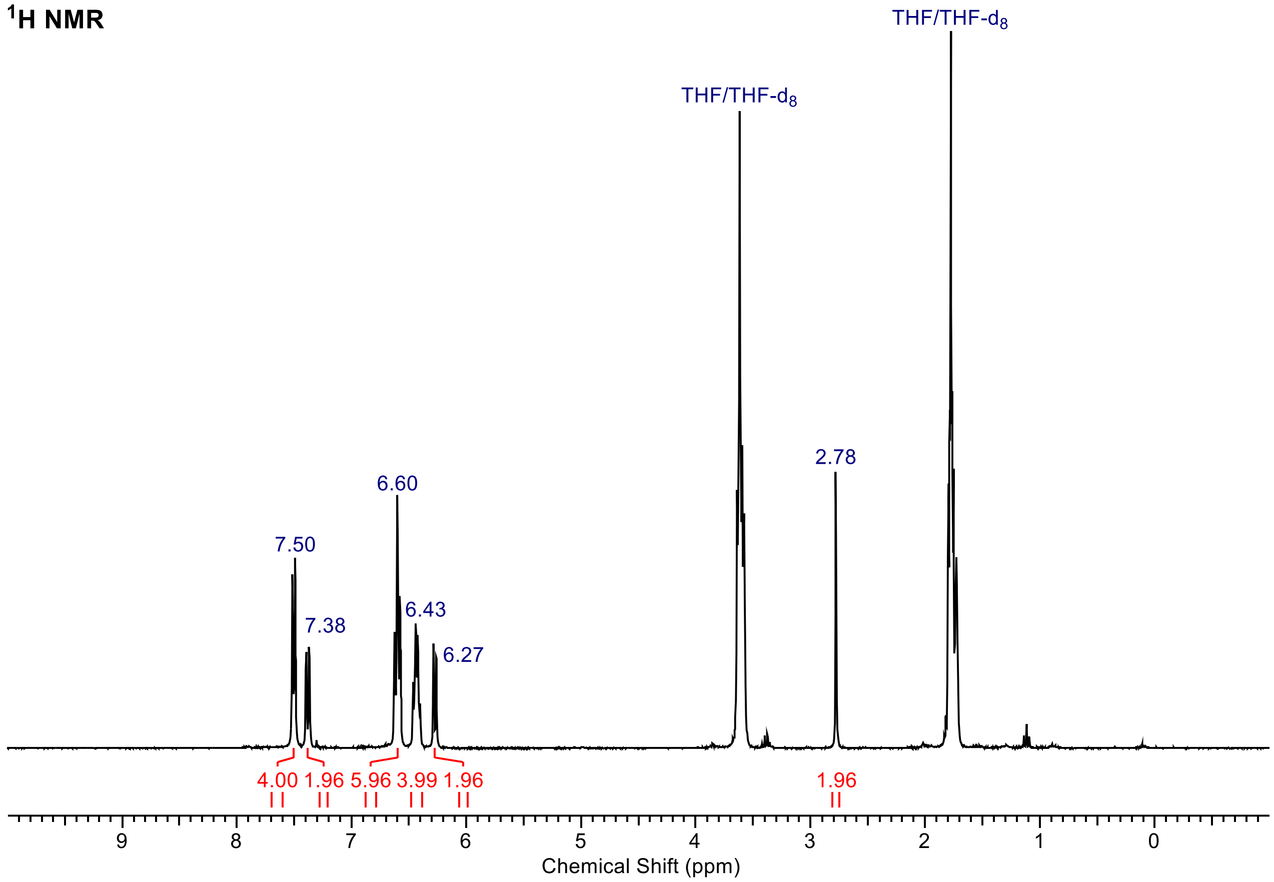


**Spectra S19:** $^{1}H$ NMR spectrum of $Li_2(THF)_4Ph_2Ni(\eta^2$-phenanthrene) (**5Li**) in THF-$d_8$.

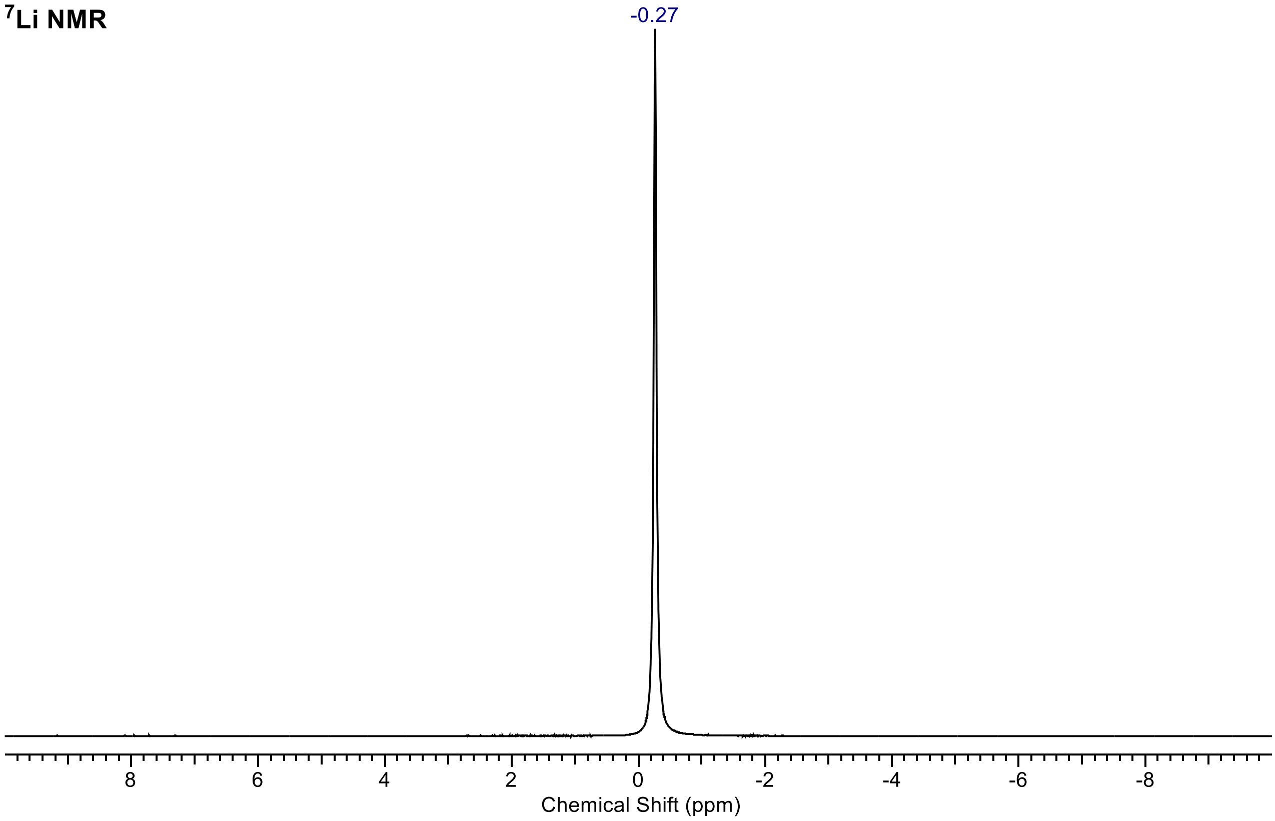


**Spectra S20:** $^{7}Li$ NMR spectrum of $Li_2(THF)_4Ph_2Ni(\eta^2$-phenanthrene) (**5Li**) in THF-$d_8$.

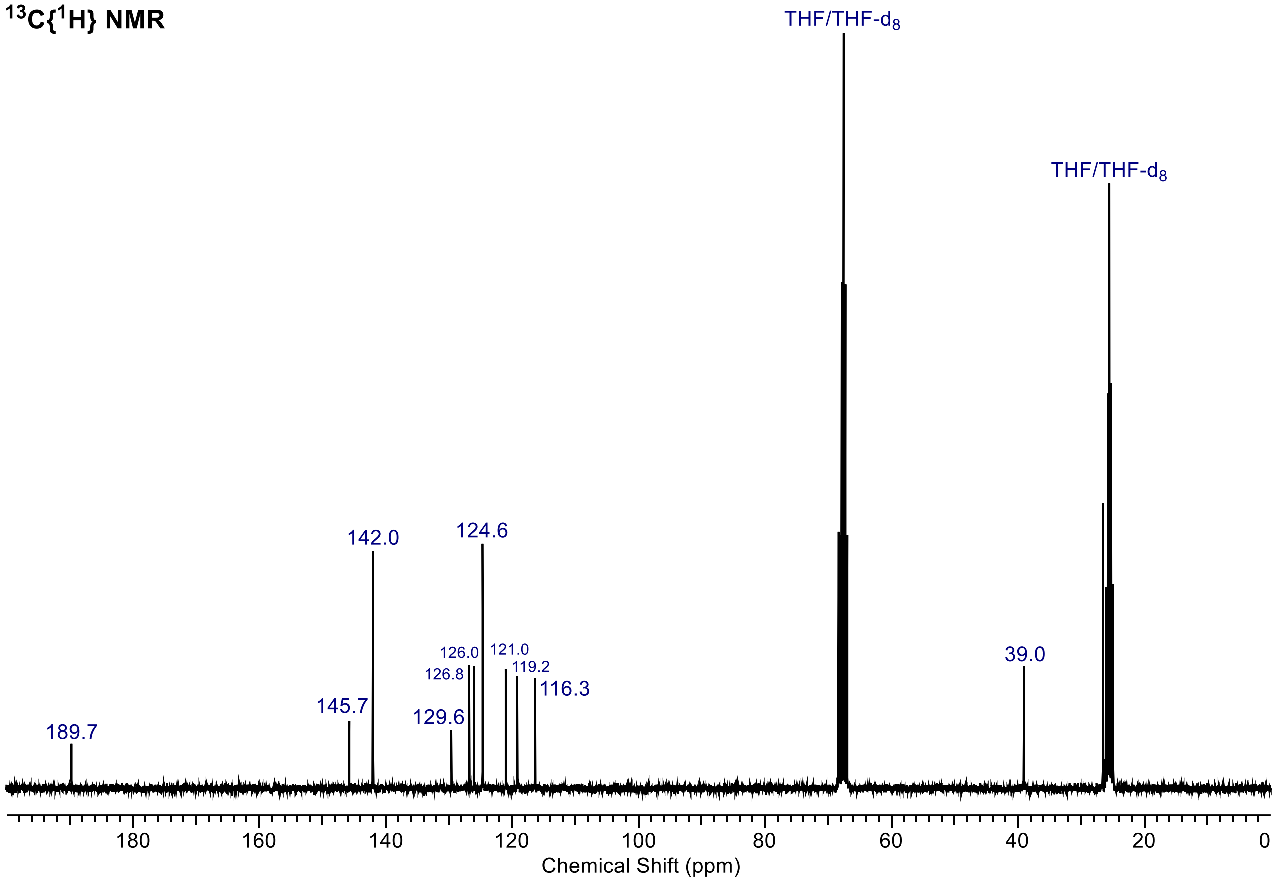


**Spectra S21:** $^{13}C\{^1H\}$ NMR spectrum of $Li_2(THF)_4Ph_2Ni(\eta^2\text{-phenanthrene})$ (**5Li**) in THF-$d_8$.

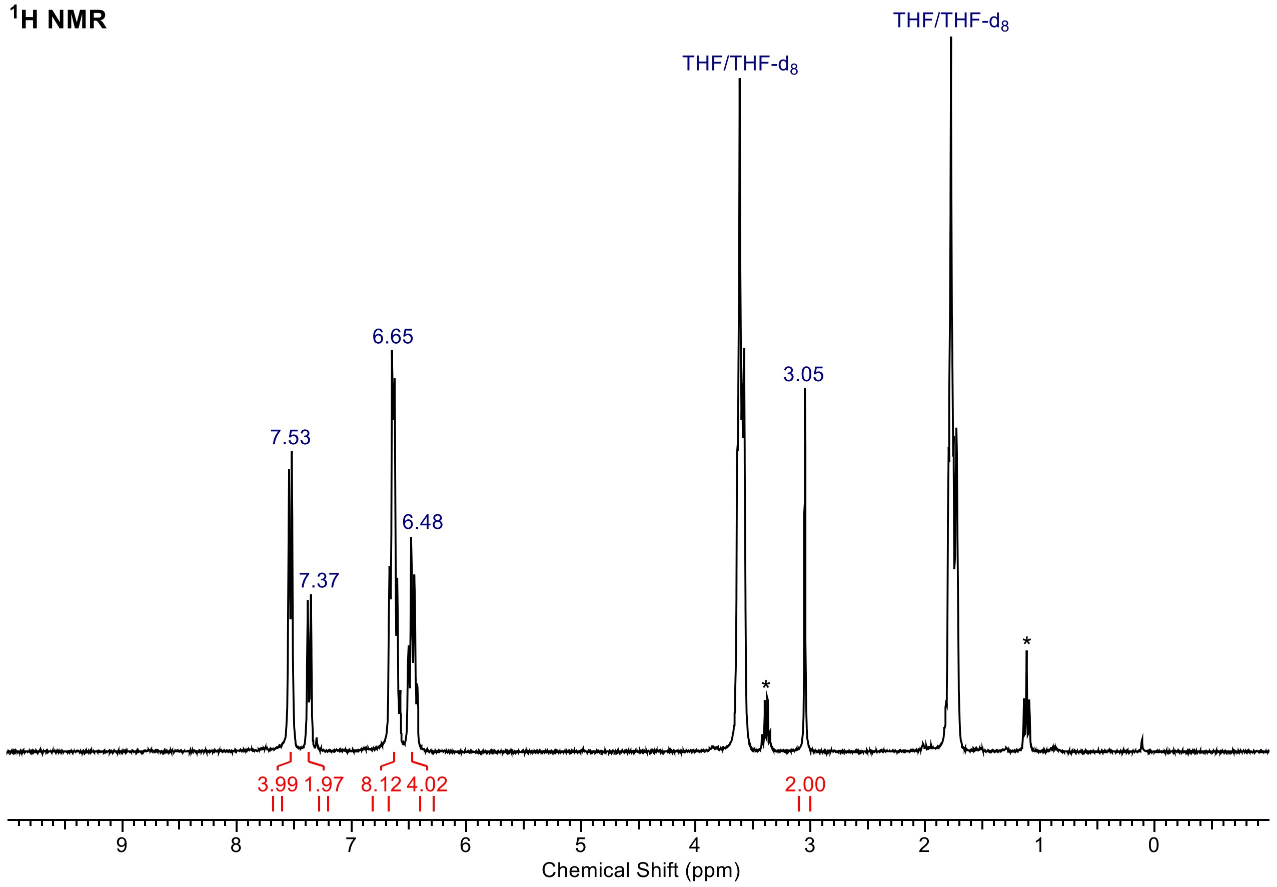


**Spectra S22:** $^1H$ NMR spectrum of $[Na_2(THF)_3Ph_2Ni(\eta^2\text{-phenanthrene})]_2$ (**5Na**) in THF-$d_8$. * Residual $Et_2O$.

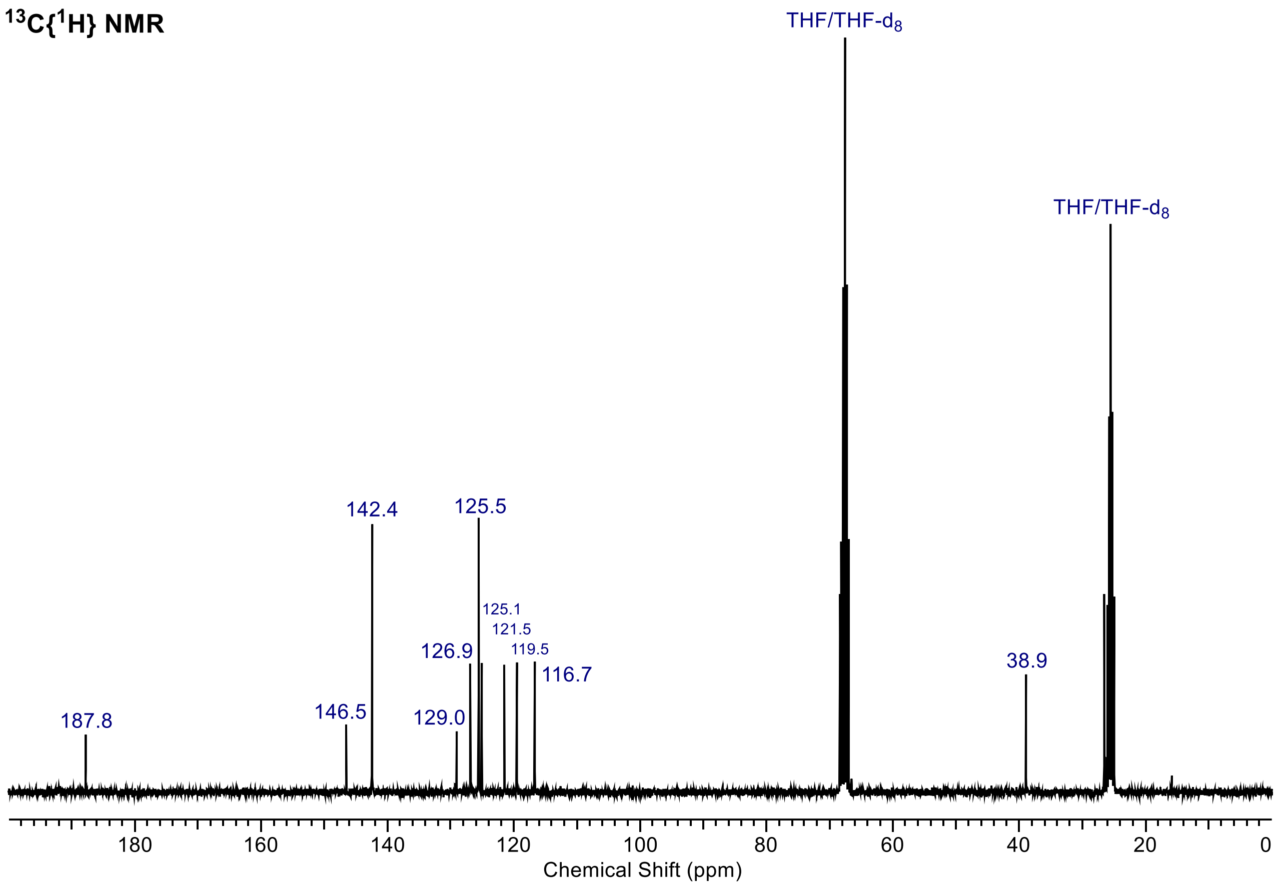


**Spectra S23:** $^{13}C\{^{1}H\}$ NMR spectrum of $[Na_2(THF)_3Ph_2Ni(\eta^2\text{-phenanthrene})]_2$ (**5Na**) in THF-$d_8$.

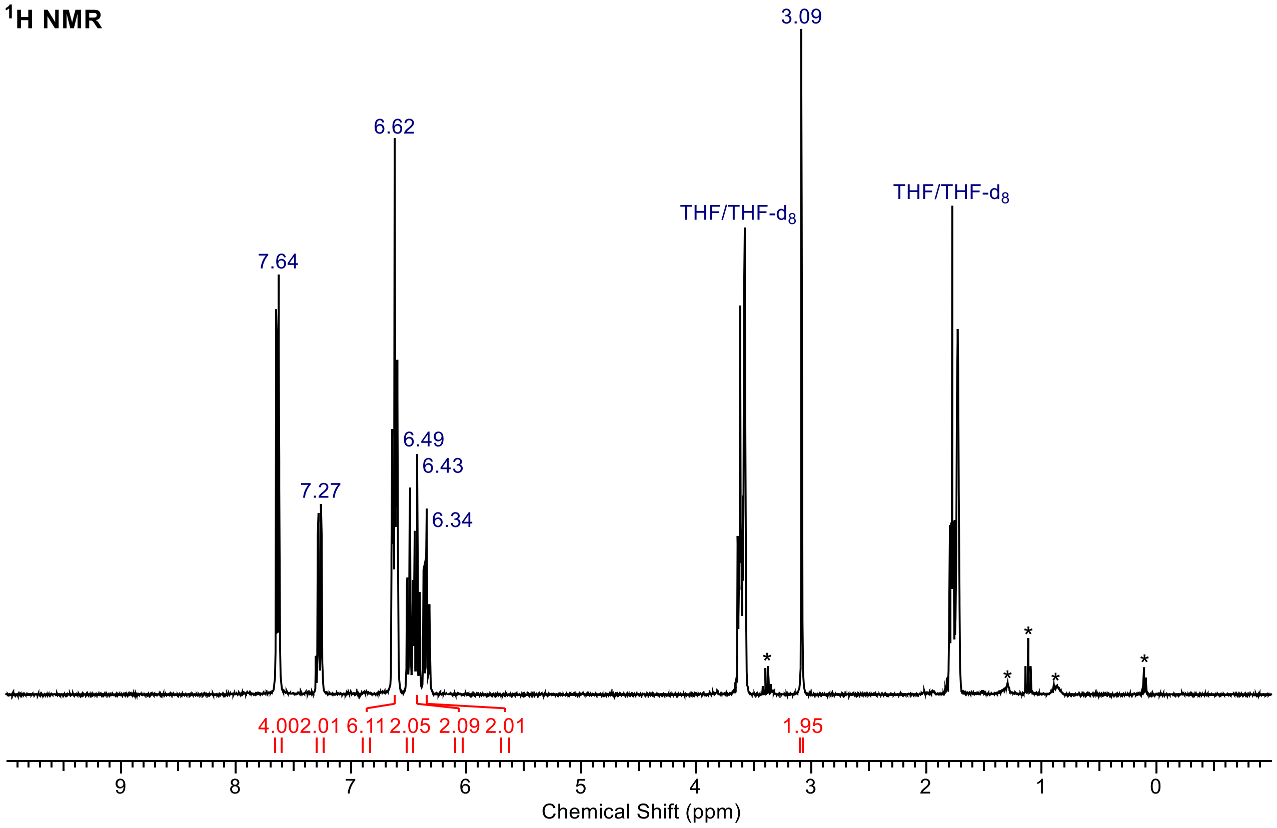


**Spectra S24:** $^{1}H$ NMR spectrum of $[K_2(THF)_1Ph_2Ni(\eta^2\text{-phenanthrene})]_\infty$ (**5K**) in THF-$d_8$. * Residual $Et_2O$ and pentane.

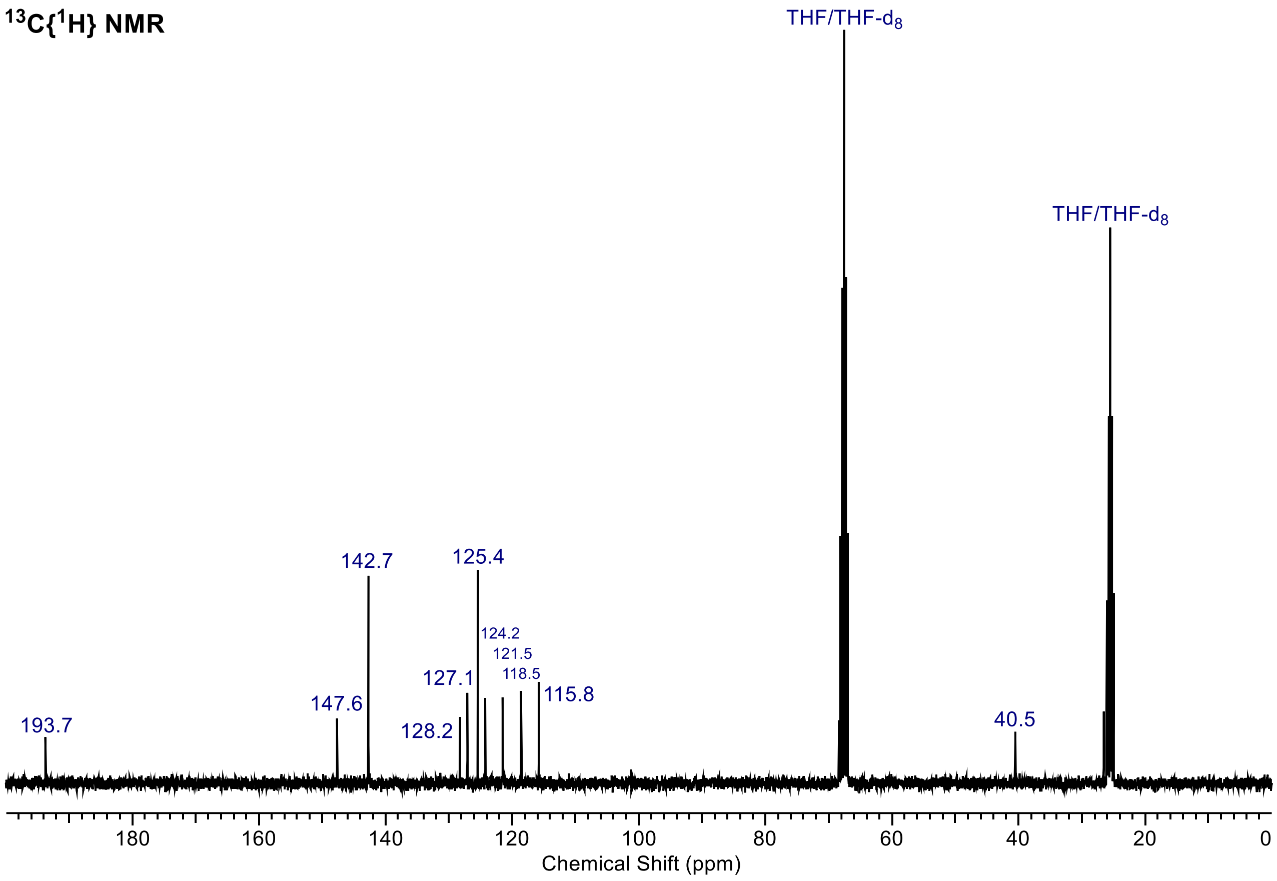


**Spectra S25:** $^{13}C\{^{1}H\}$ NMR spectrum of $[K_2(THF)_1Ph_2Ni(\eta^2\text{-phenanthrene})]_\infty$ (**5K**) in THF-$d_8$.

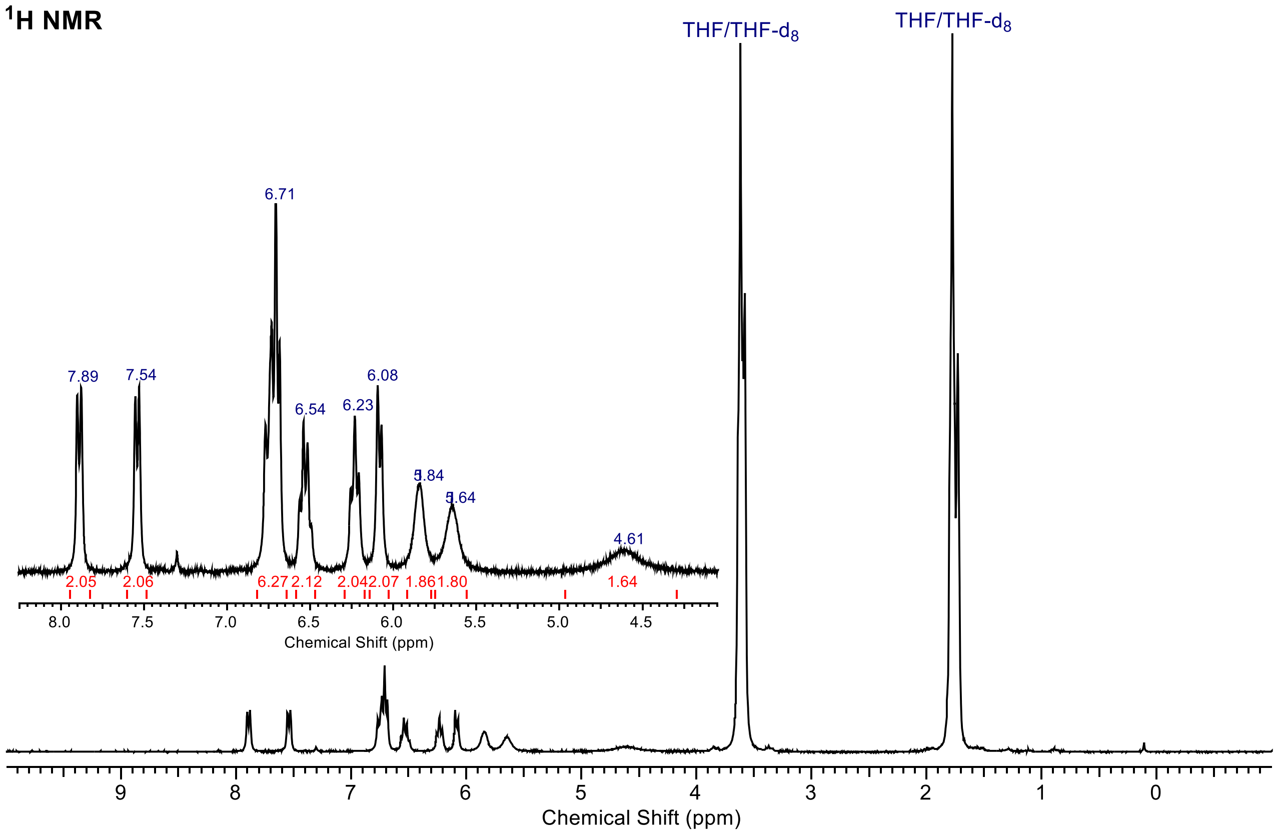


**Spectra S26:** $^{1}H$ NMR spectrum of $[Li(THF)_2Ph_2Ni(\eta^3\text{-perylene})][Li(THF)_4]$ (**6Li**) in THF-$d_8$.

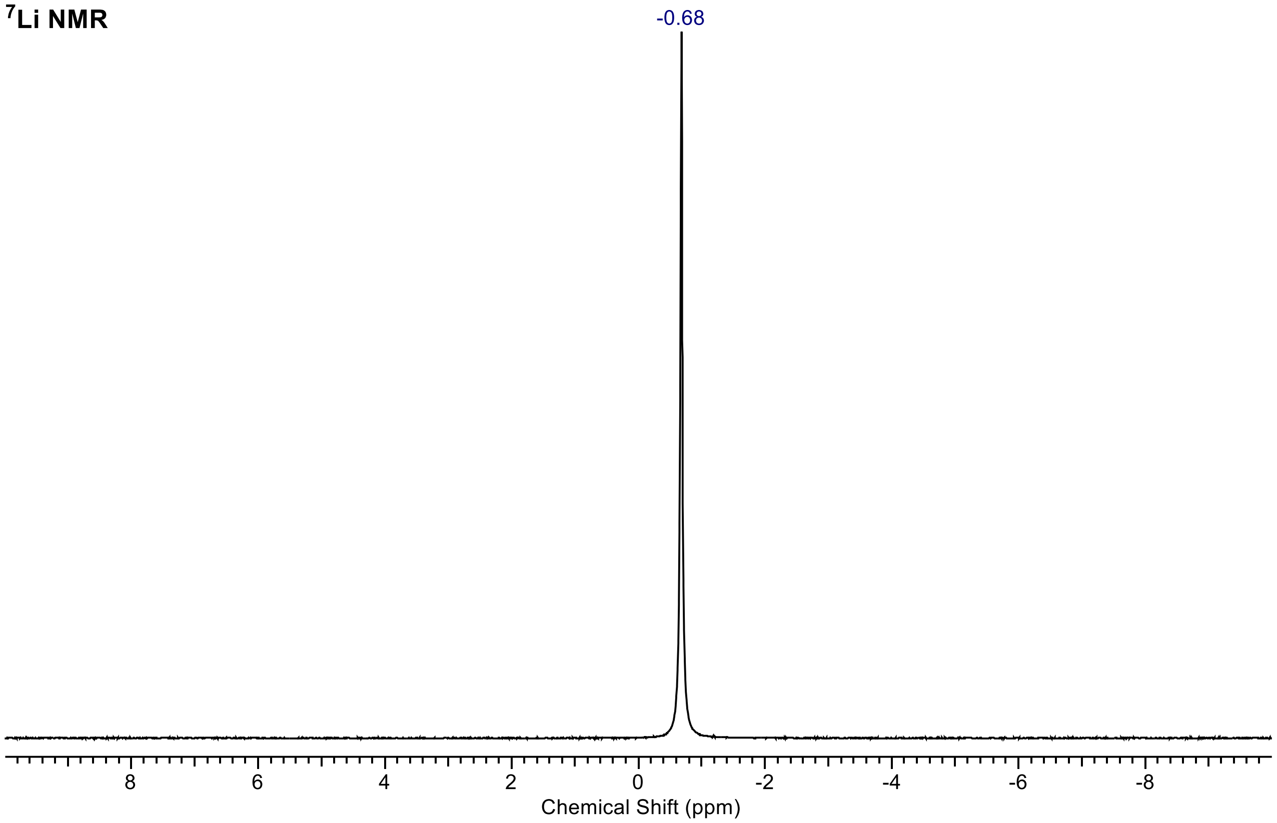


**Spectra S27:** $^{7}$Li NMR spectrum of [Li(THF)$_2$Ph$_2$Ni($\eta^3$-perylene)][Li(THF)$_4$] (**6Li**) in THF-d$_8$.

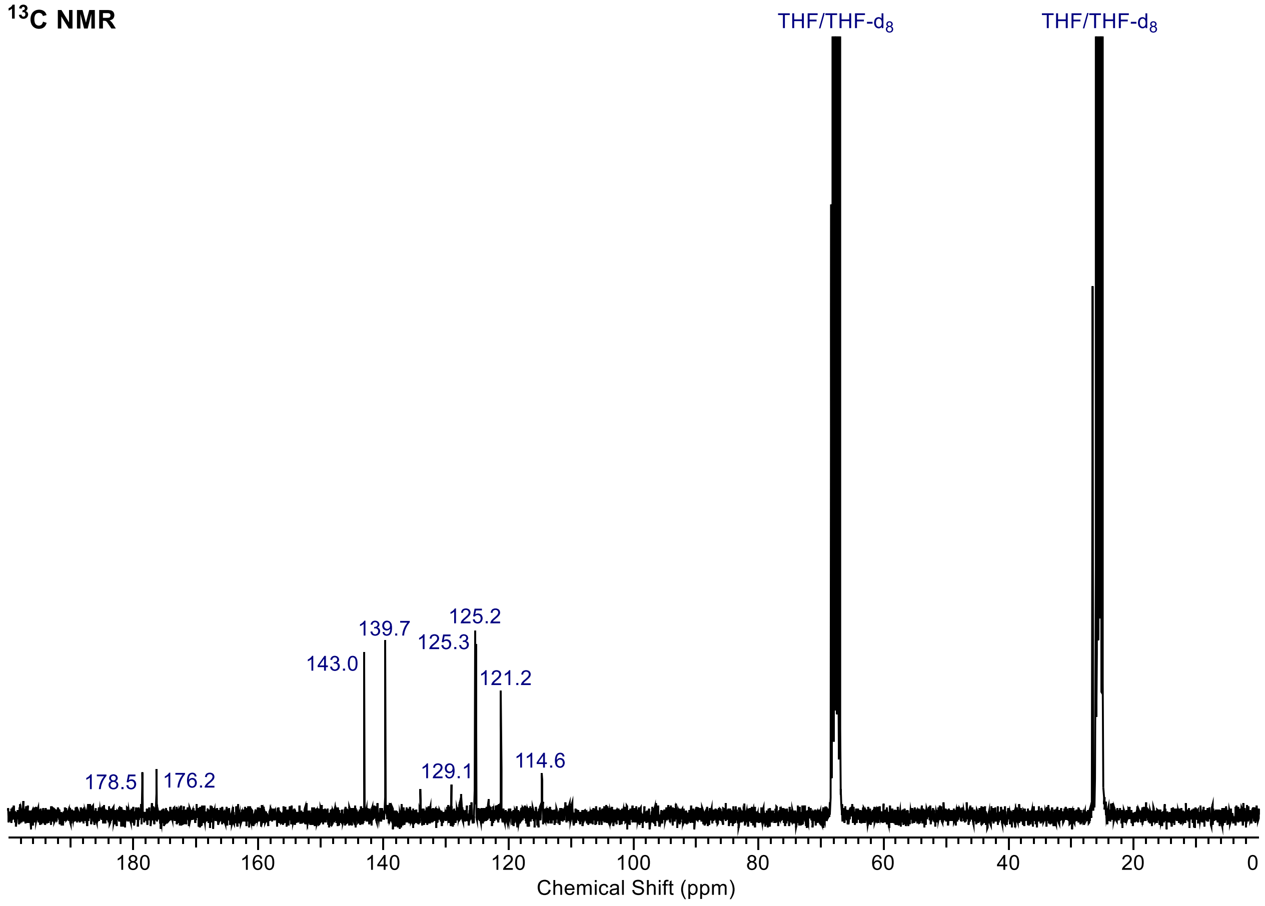


**Spectra S28:** $^{13}$C{$^{1}$H} NMR spectrum of [Li(THF)$_2$Ph$_2$Ni($\eta^3$-perylene)][Li(THF)$_4$] (**6Li**) in THF-d$_8$.

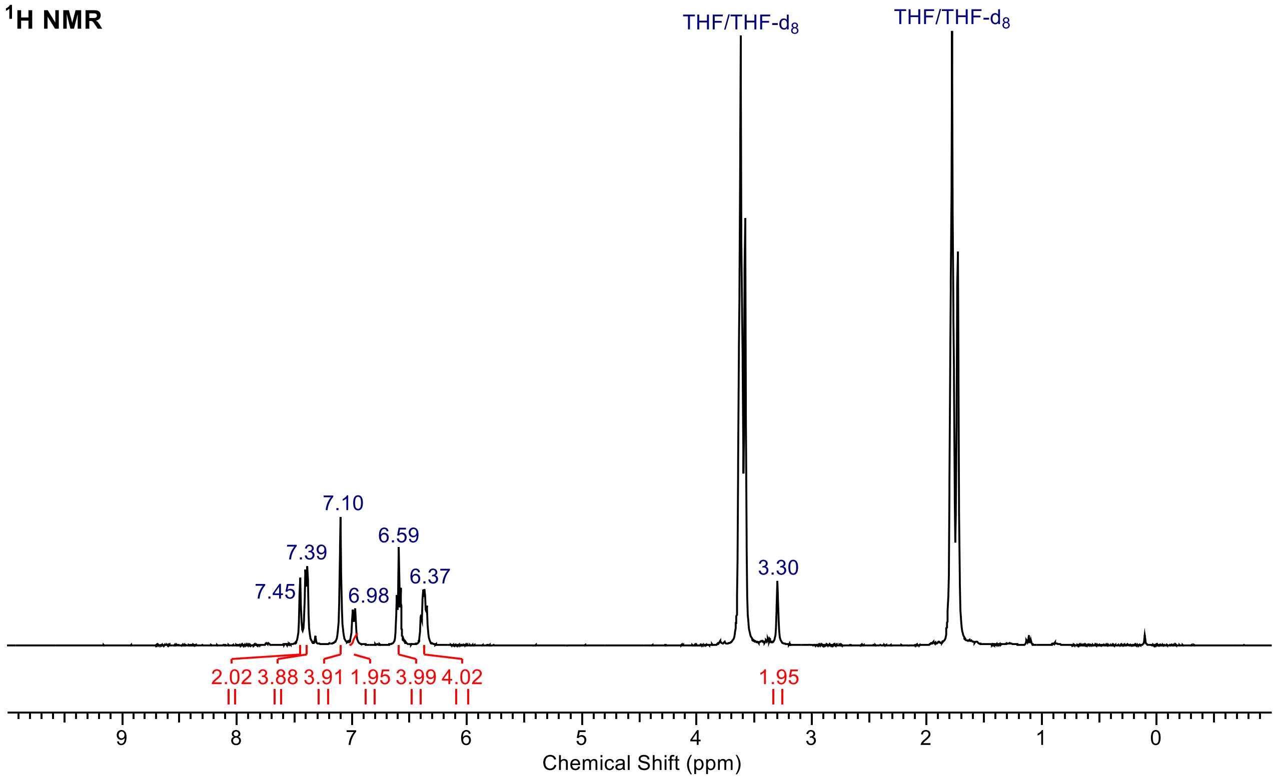


**Spectra S29:** $^{1}$H NMR spectrum of $[Li(THF)_2Ph_2Ni(\eta^2\text{-coronene})][Li(THF)_4]$ (**7Li**) in THF-$d_8$ at -20 °C.

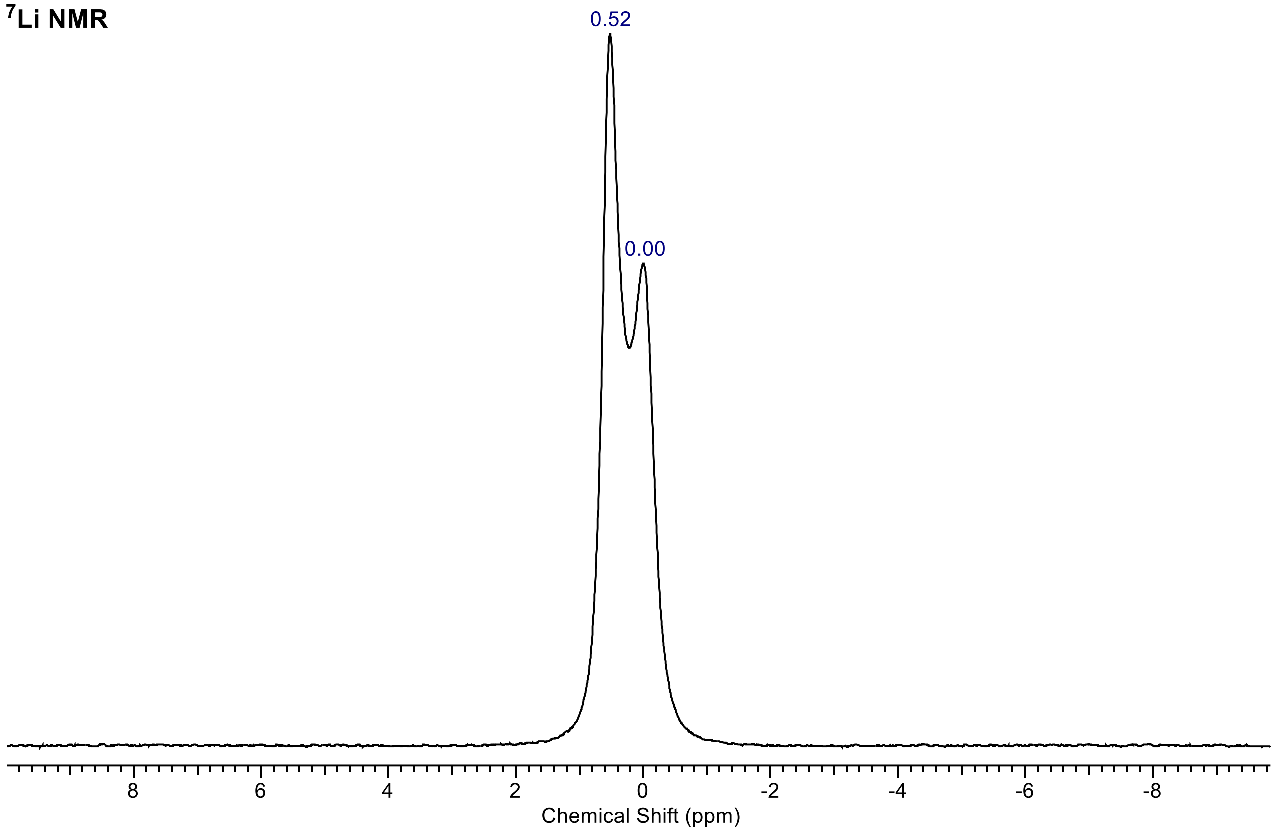


**Spectra S30:** $^{7}$Li NMR spectrum of $[Li(THF)_2Ph_2Ni(\eta^2\text{-coronene})][Li(THF)_4]$ (**7Li**) in THF-$d_8$ at -40 °C.

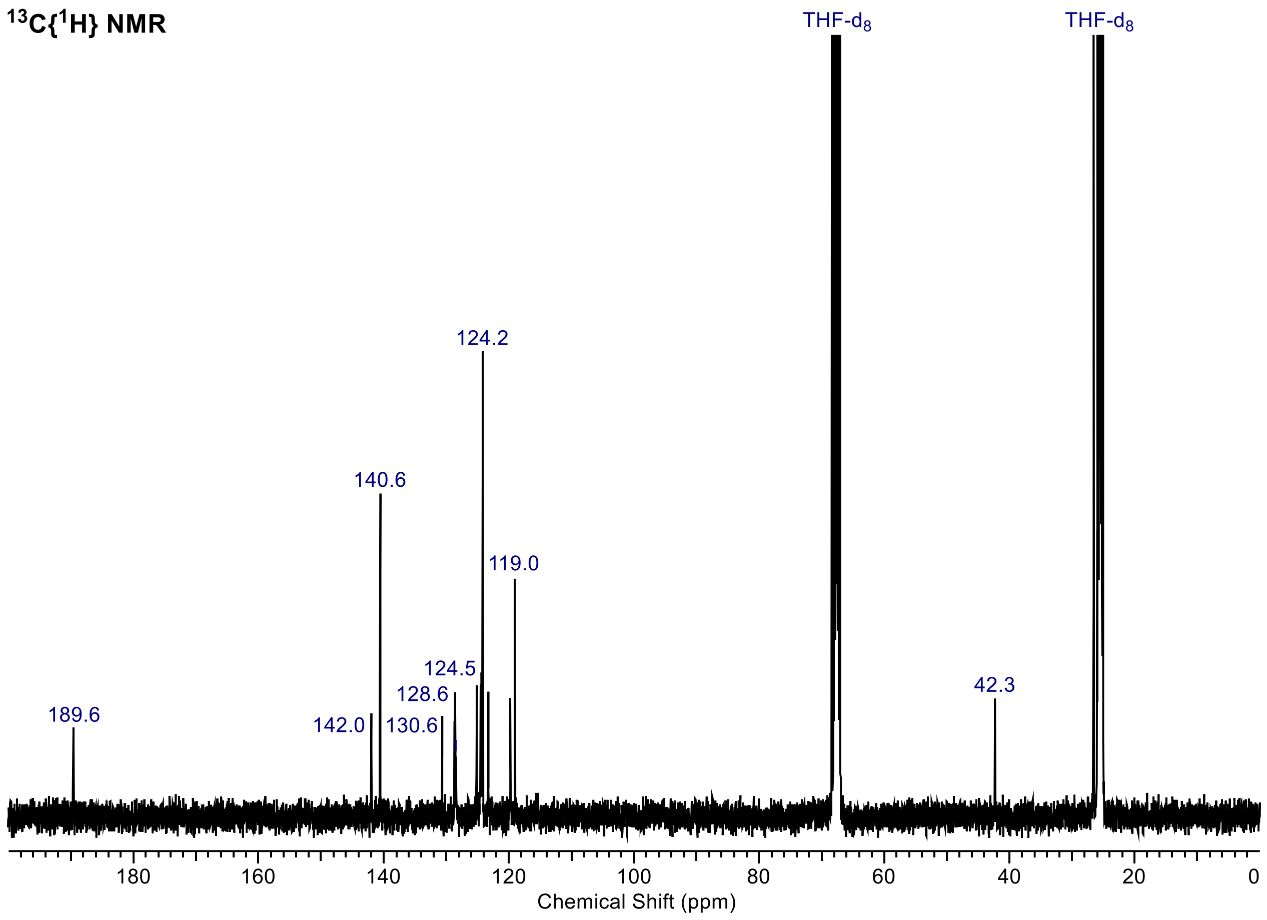


**Spectra S31:** $^{13}C\{^1H\}$ NMR spectrum of $[Li(THF)_2Ph_2Ni(\eta^2\text{-coronene})][Li(THF)_4]$ (**7Li**) in THF-$d_8$ at -20 °C.

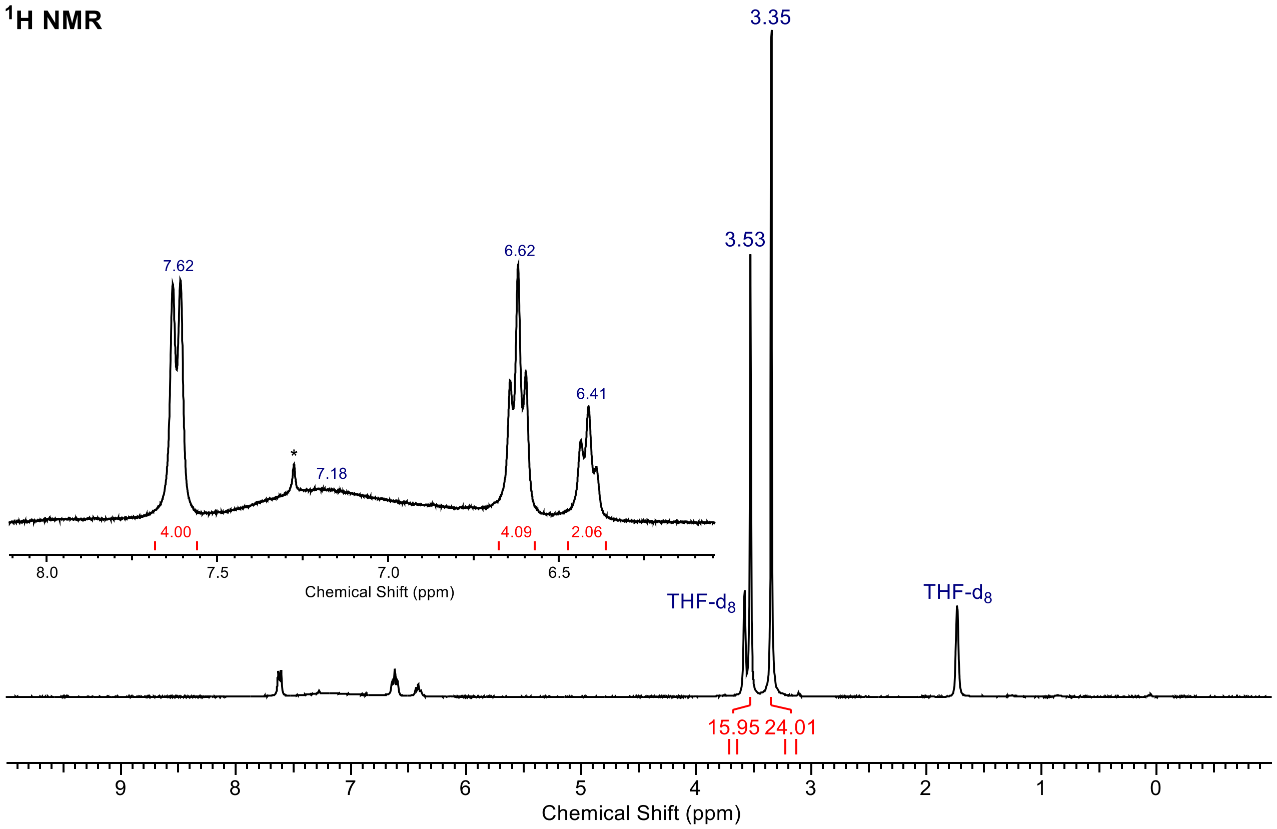


**Spectra S32:** $^1H$ NMR spectrum of $K_2(DME)_4Ph_2Ni(\eta^2\text{-coronene})$ (**7K**) in THF-$d_8$.

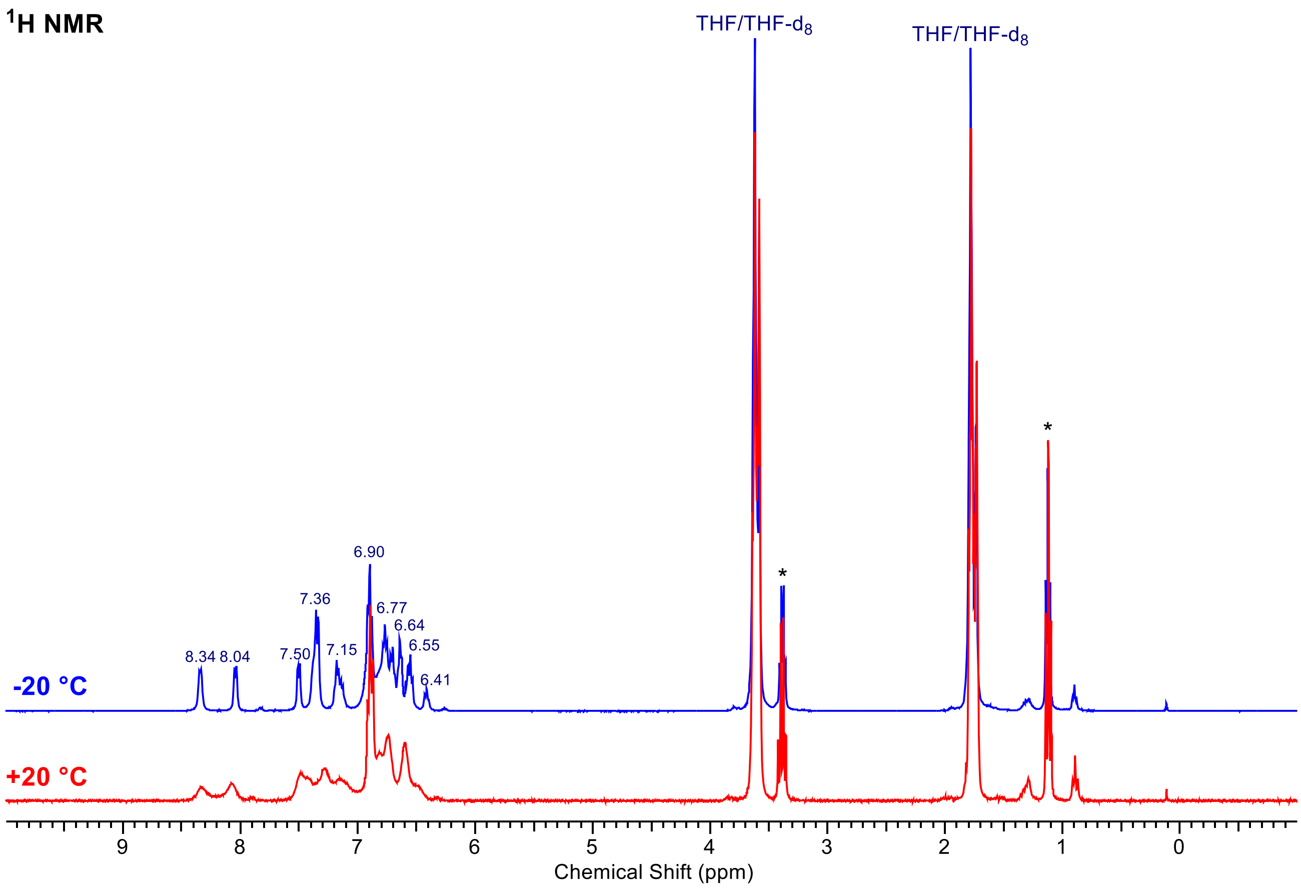


**Spectra S33:** Stacked [1]H NMR spectra of $[Li_2(THF)_3Ph_2Ni(\eta^2\text{-}Ph_2CO)]_2$ (**8Li**) in THF-$d_8$ at -20 °C (*blue trace*) and +20 °C (*red trace*). * Residual $Et_2O$.

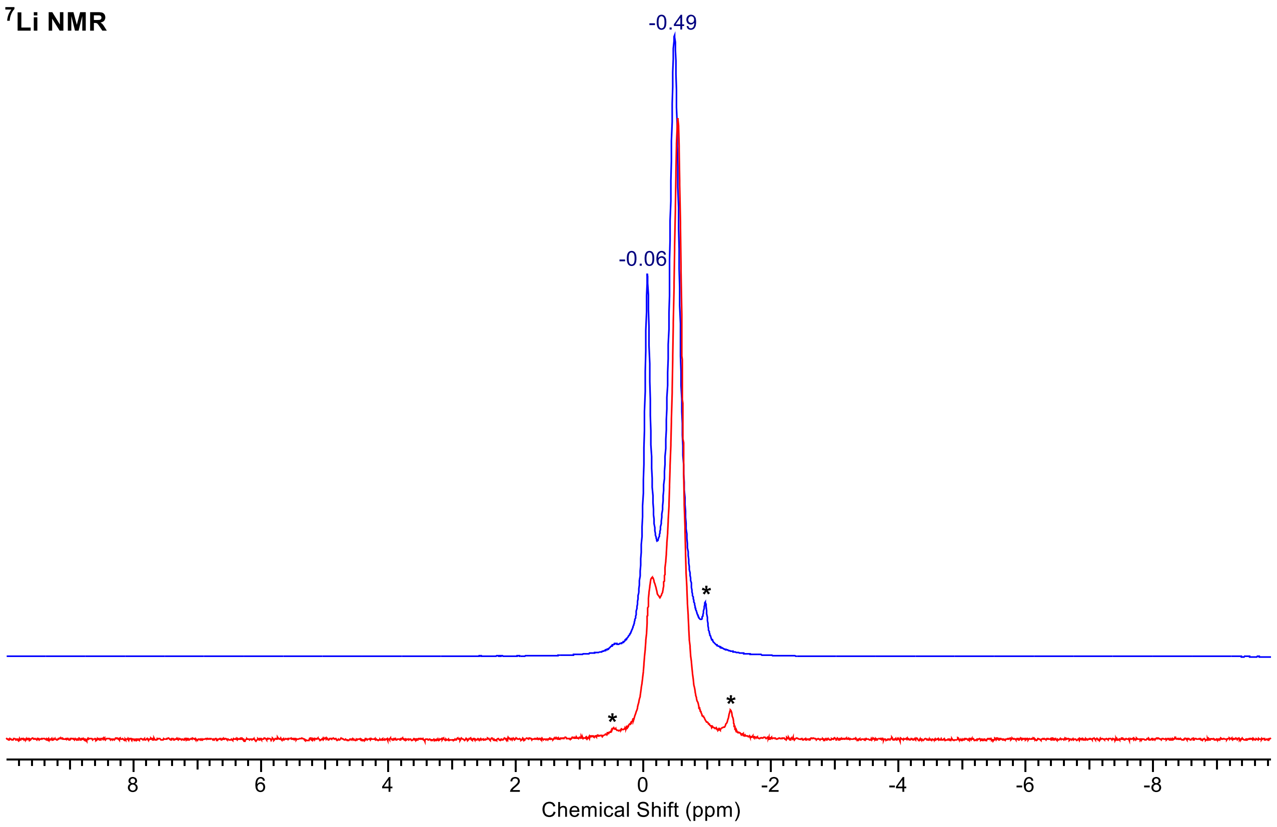


**Spectra S34:** Stacked [7]Li NMR spectra of $[Li_2(THF)_3Ph_2Ni(\eta^2\text{-}Ph_2CO)]_2$ (**8Li**) in THF-$d_8$ at -20 °C (*blue trace*) and +20 °C (*red trace*). * Unidentified impurities.

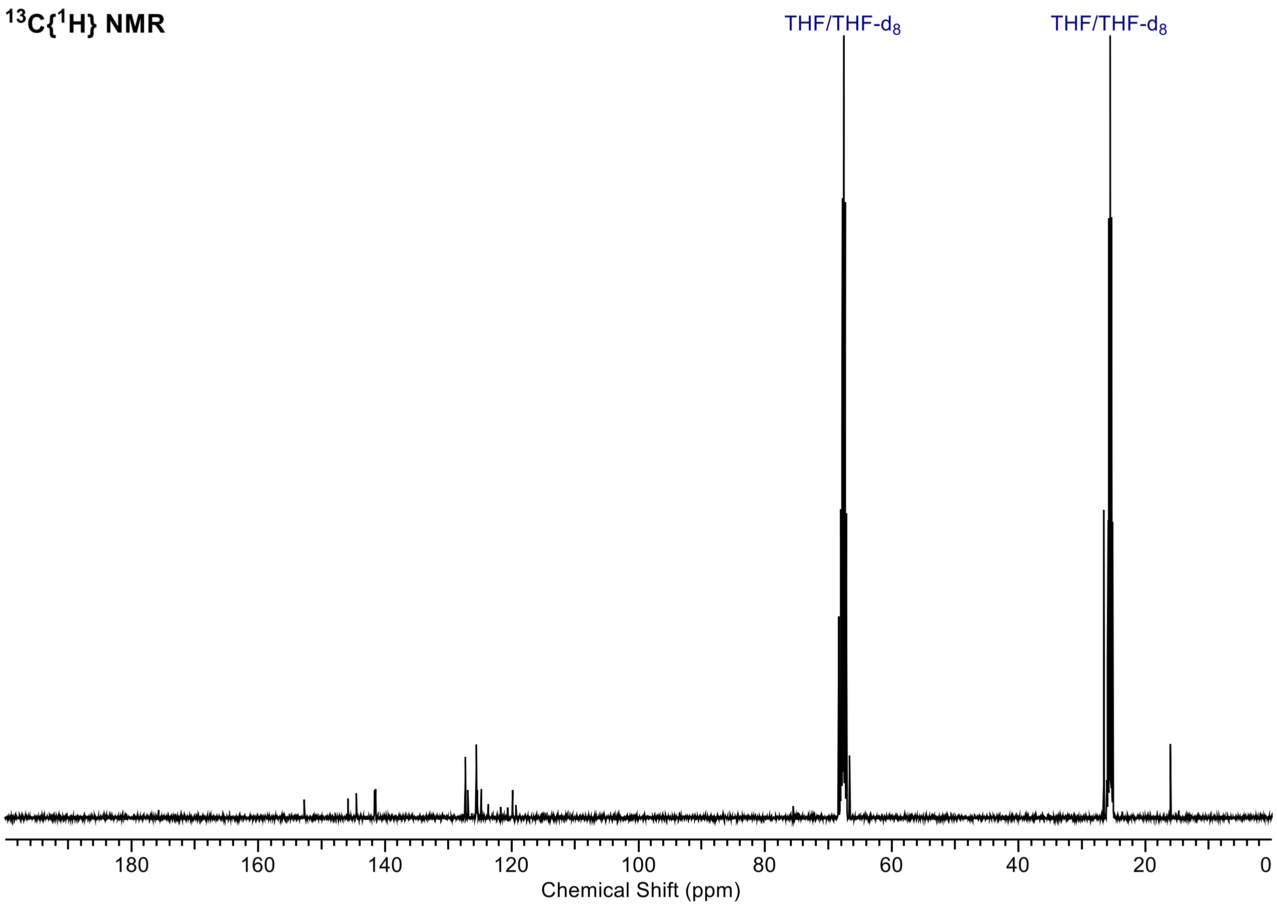


**Spectra S35:** $^{13}C\{^1H\}$ NMR spectra of [$Li_2(THF)_3Ph_2Ni(\eta^2$-$Ph_2CO)]_2$ (**8Li**) in THF-$d_8$ at -20 °C.

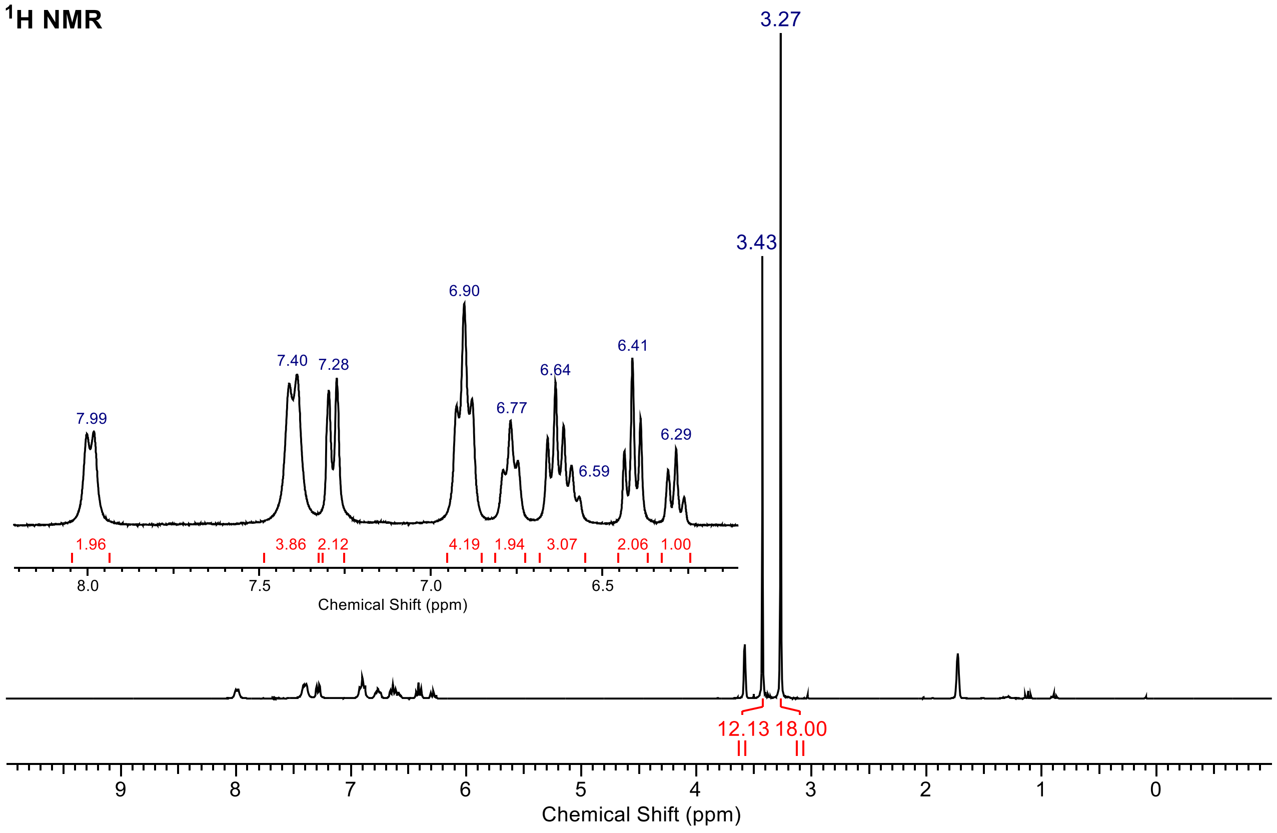


**Spectra S36:** $^1H$ NMR spectrum of [$K_2(DME)_3Ph_2Ni(\eta^2$-$Ph_2CO)]_2$ (**8K**) in THF-$d_8$.

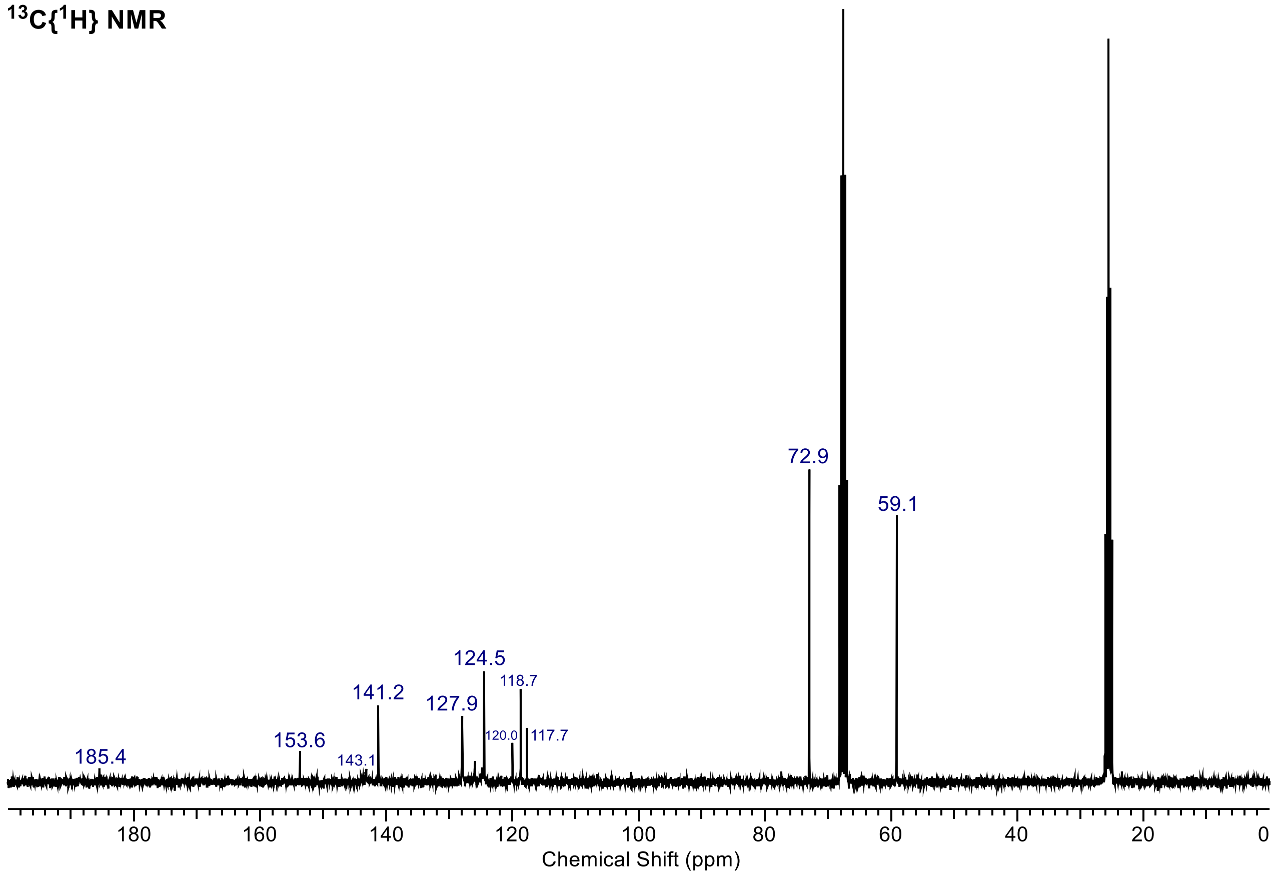


**Spectra S37:** $^{13}C\{^1H\}$ NMR spectrum of $[K_2(DME)_3Ph_2Ni(\eta^2\text{-}Ph_2CO)]_2$ (**8K**) in THF-$d_8$.

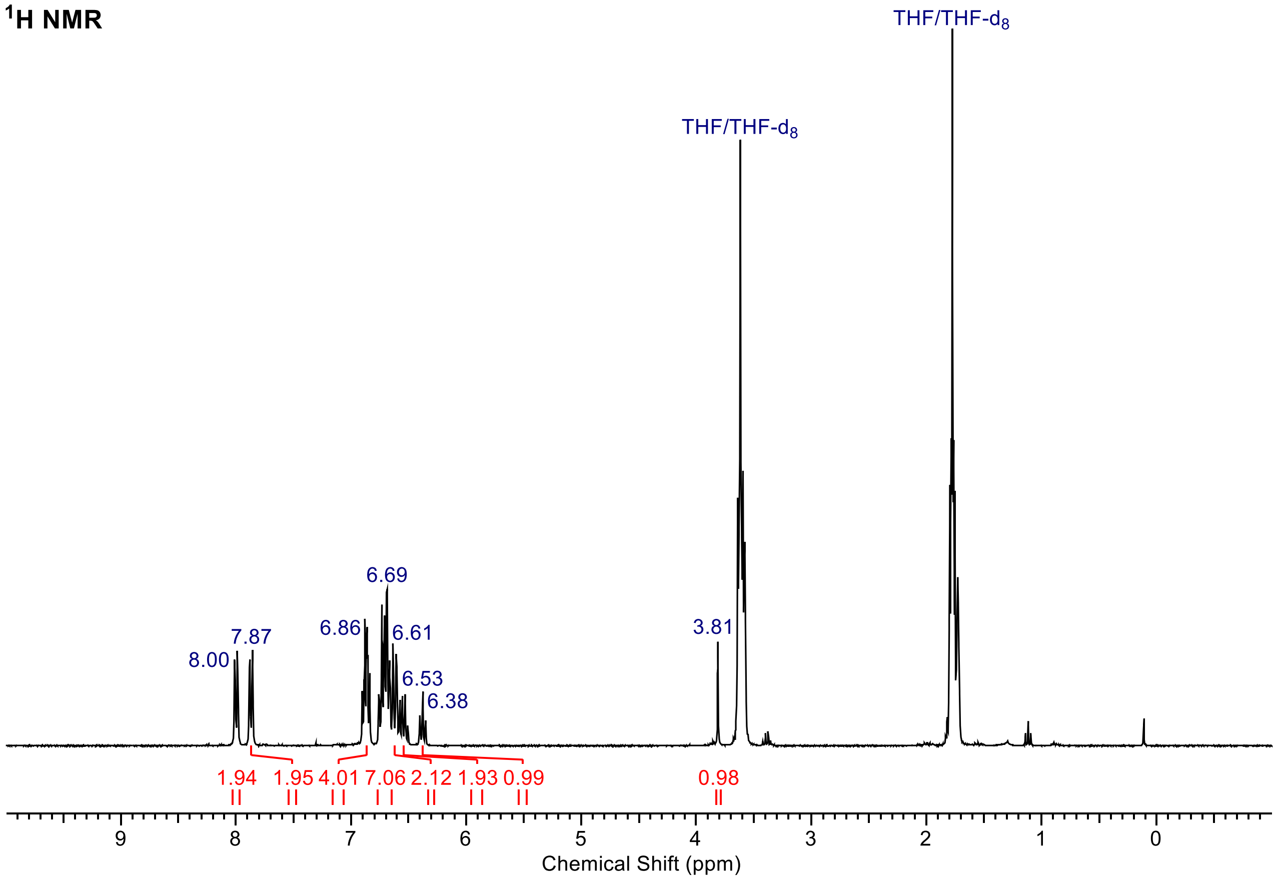


**Spectra S38:** $^1H$ NMR spectrum of $Li_2(THF)_5Ph_2Ni(\eta^2\text{-}PhCH{=}NPh)$ (**9Li**) in THF-$d_8$.

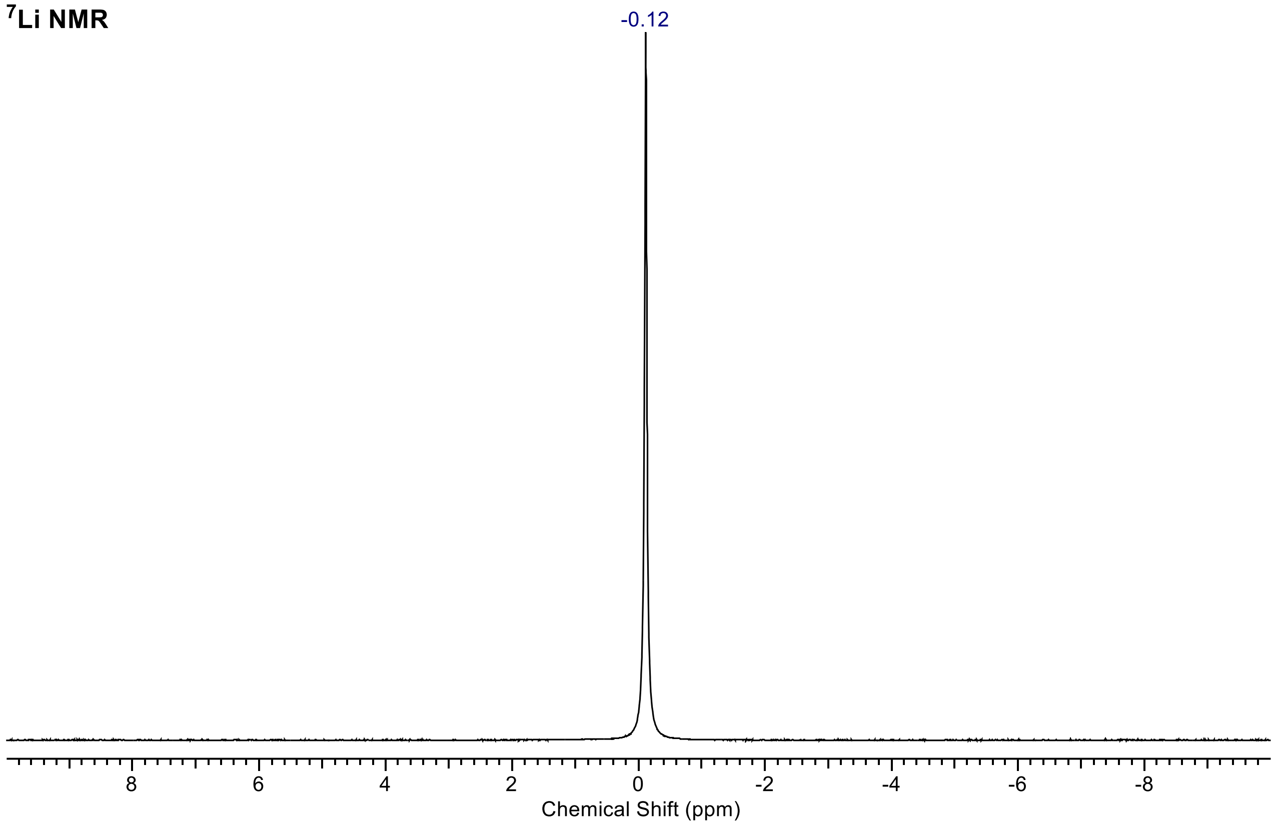


**Spectra S39:** $^{7}$Li NMR spectrum of $Li_2(THF)_5Ph_2Ni(\eta^2$-PhCH=NPh) (**9Li**) in THF-$d_8$.

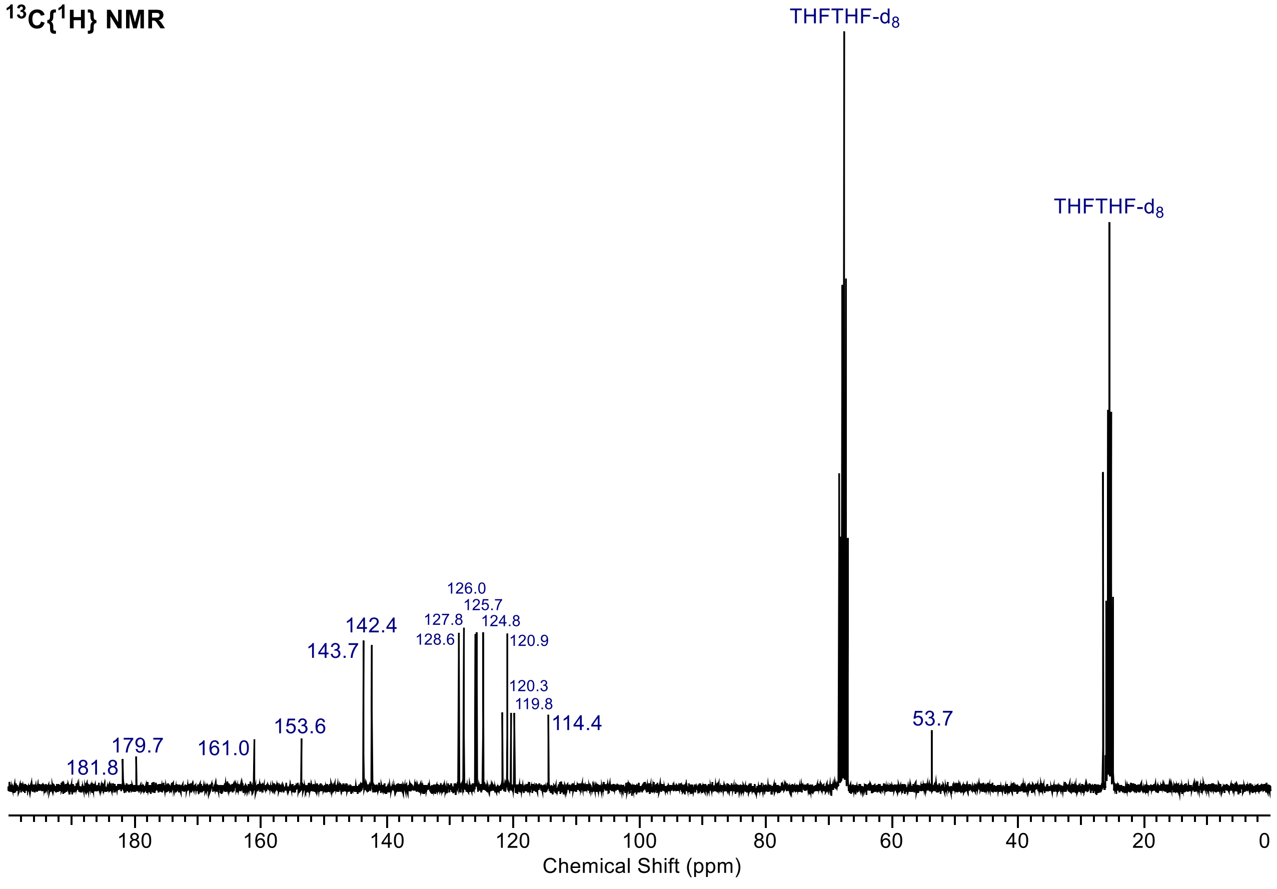


**Spectra S40:** $^{13}$C{$^{1}$H} NMR spectrum of $Li_2(THF)_5Ph_2Ni(\eta^2$-PhCH=NPh) (**9Li**) in THF-$d_8$.

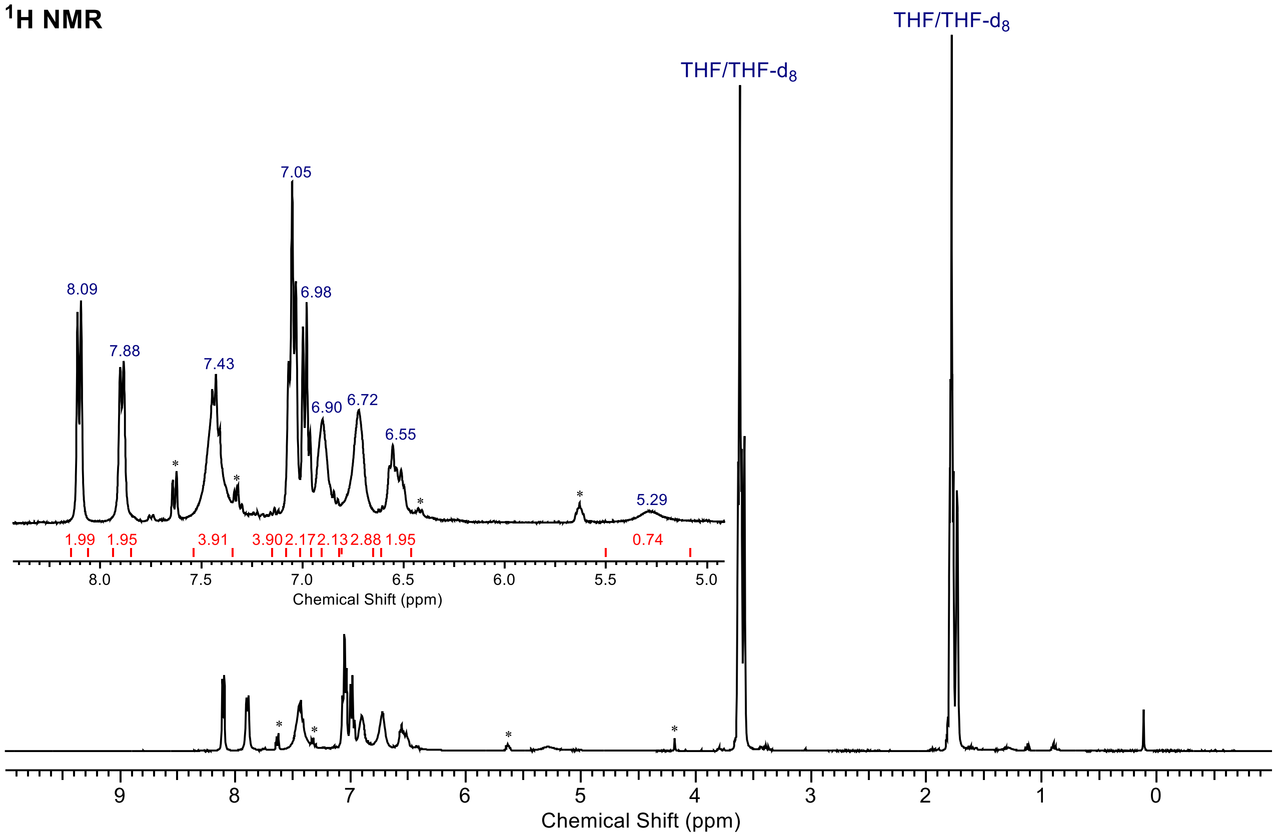


**Spectra S41:** $^{1}$H NMR spectrum of $[Li_2(THF)_3Ph_2Ni(\eta^2\text{-}Ph_2CHCN)]_2$ (**10Li**) in THF-$d_8$ at -20 °C. * Unidentified impurities/decomposition products.

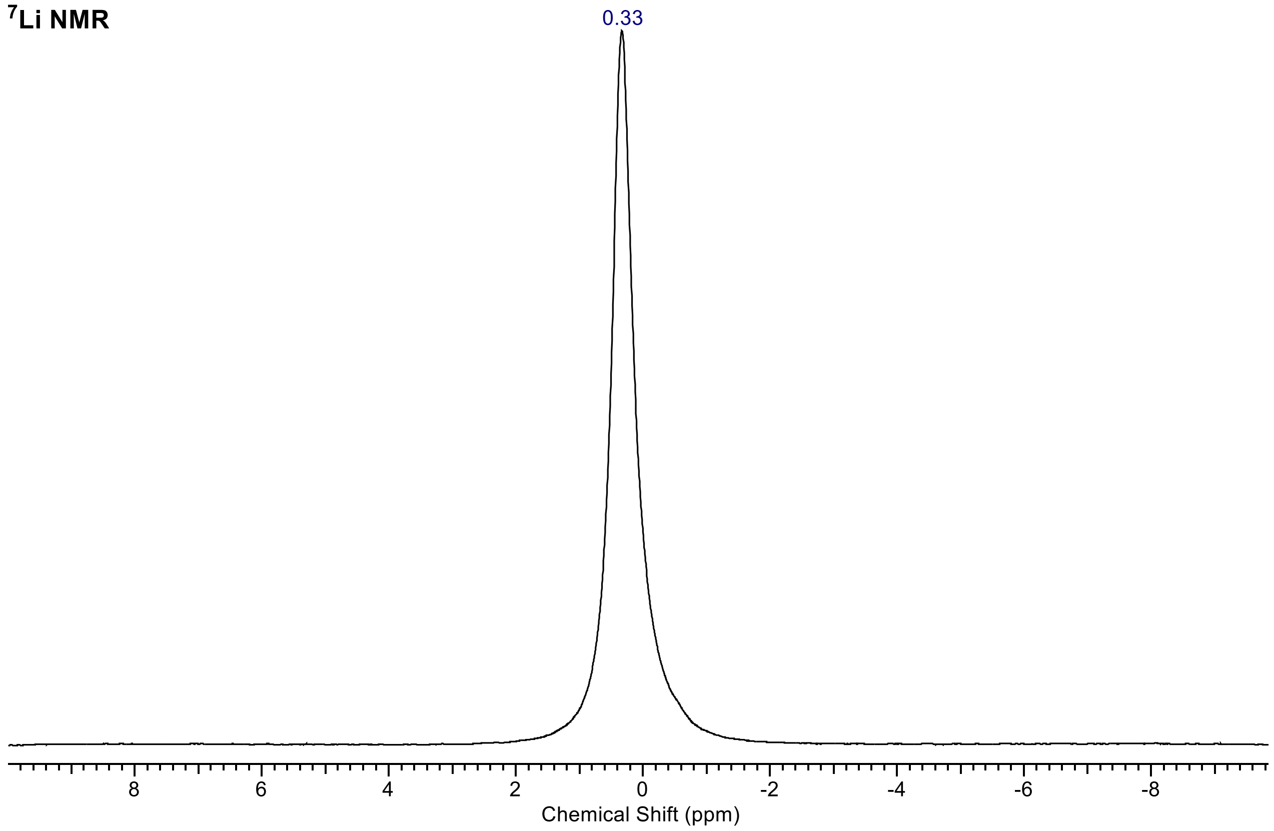


**Spectra S42:** $^{7}$Li NMR spectrum of $[Li_2(THF)_3Ph_2Ni(\eta^2\text{-}Ph_2CHCN)]_2$ (**10Li**) in THF-$d_8$ at -20 °C.

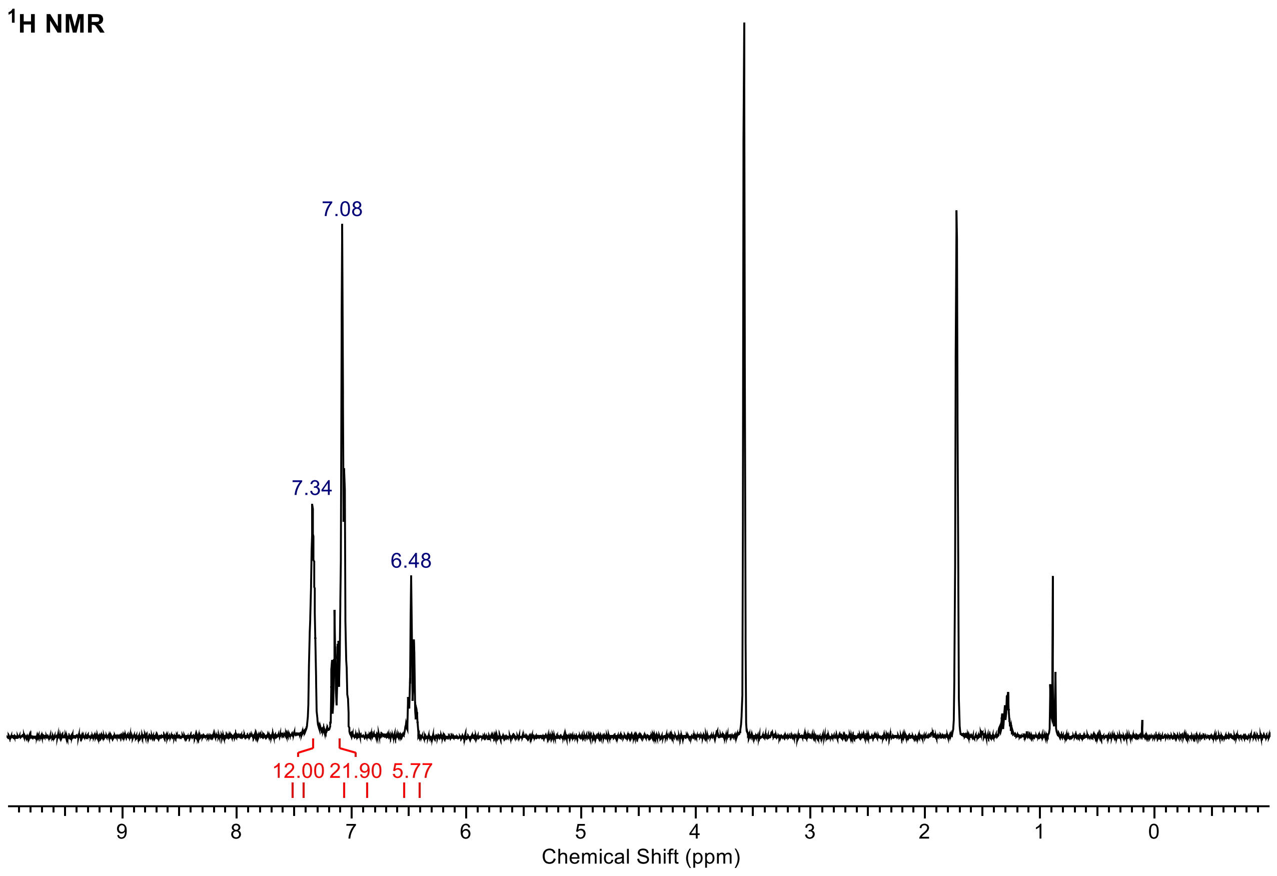


**Spectra S43:** $^1$H NMR spectrum of Li(THF)$_3$(PPh$_3$)$_2$(CO)$_3$Ni$_2$($\mu$-PPh$_2$) (**11Li**) in THF-d$_8$.

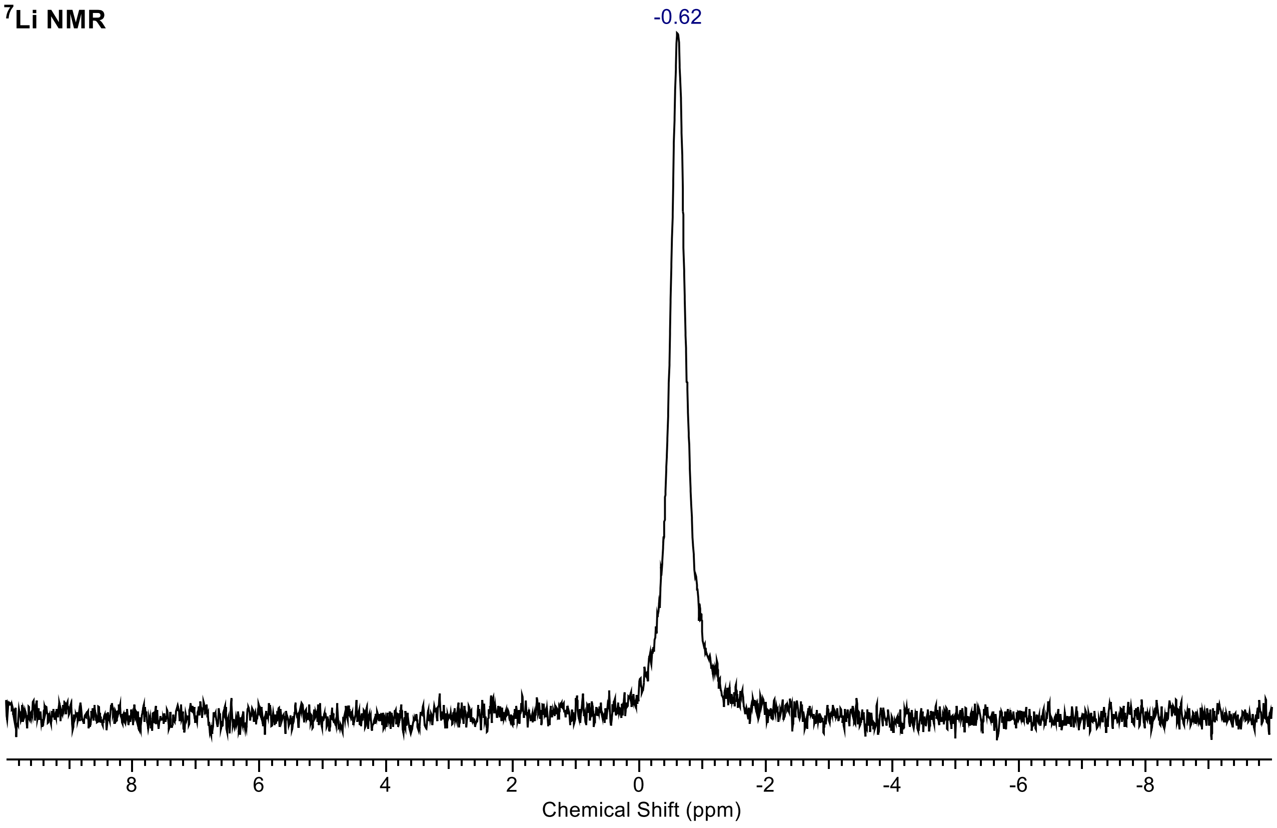


**Spectra S44:** $^7$Li NMR spectrum of Li(THF)$_3$(PPh$_3$)$_2$(CO)$_3$Ni$_2$($\mu$-PPh$_2$) (**11Li**) in THF-d$_8$.

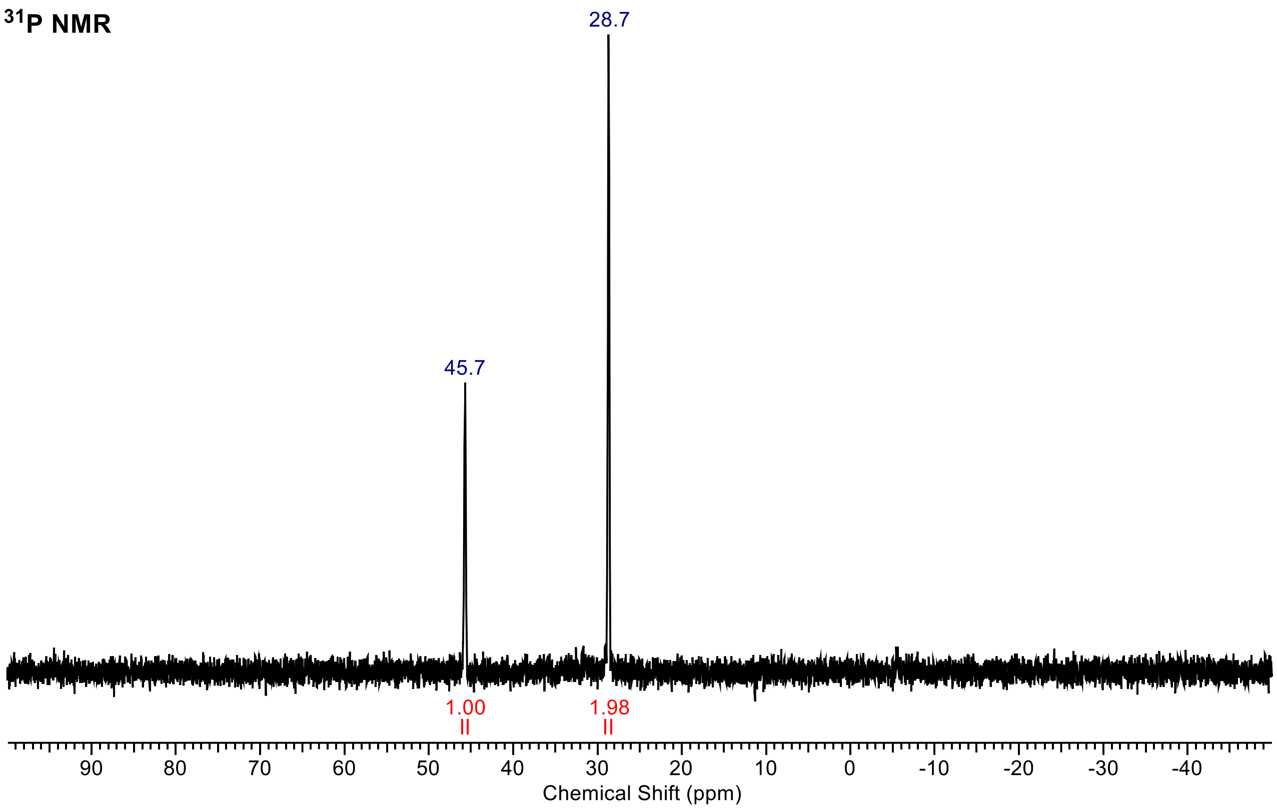


**Spectra S45:** $^{31}$P NMR spectrum of Li(THF)$_3$(PPh$_3$)$_2$(CO)$_3$Ni$_2$($\mu$-PPh$_2$) (**11Li**) in THF-d$_8$.

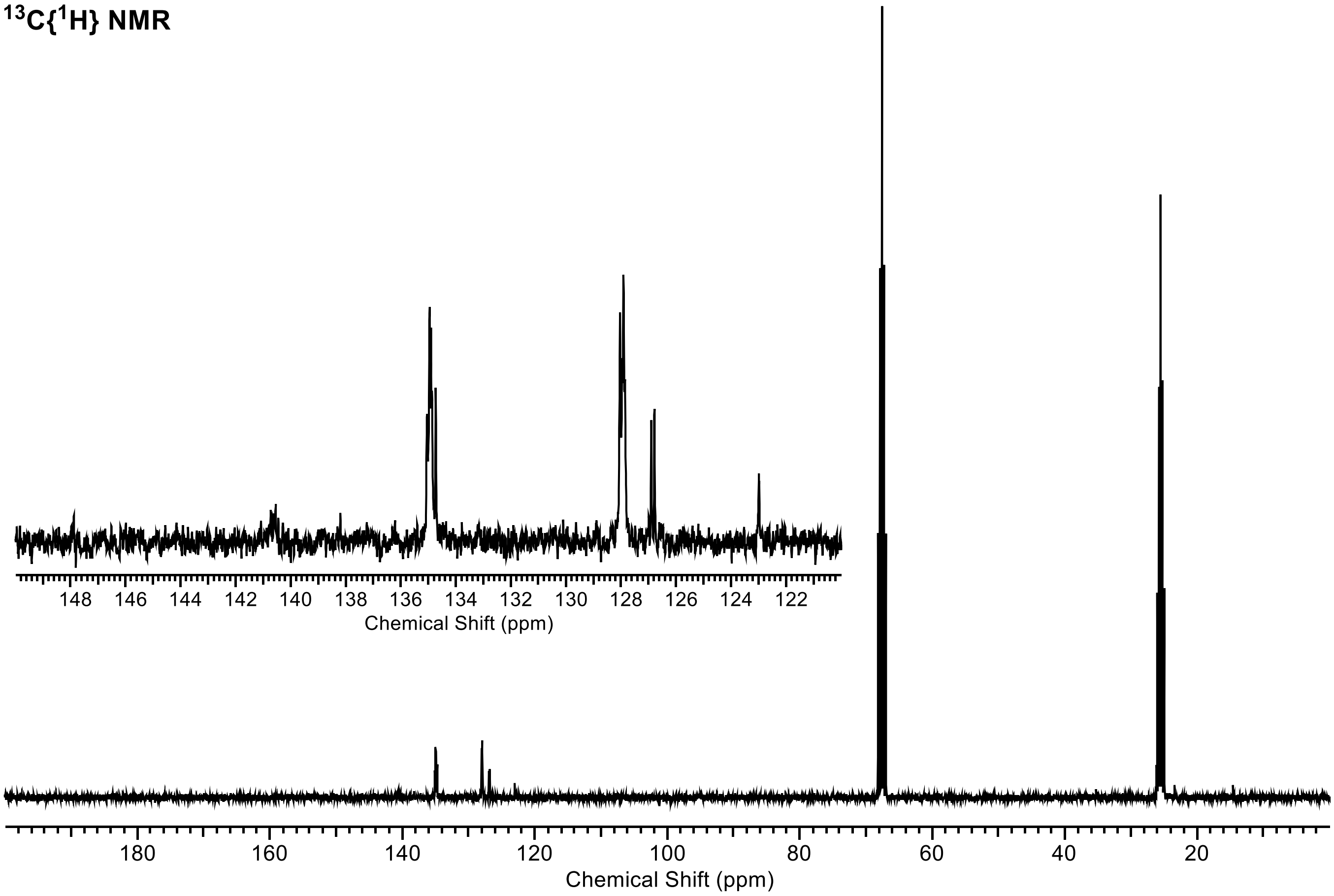


**Spectra S46:** $^{13}$C{$^1$H} NMR spectrum of Li(THF)$_3$(PPh$_3$)$_2$(CO)$_3$Ni$_2$($\mu$-PPh$_2$) (**11Li**) in THF-d$_8$.

## Reference Spectra

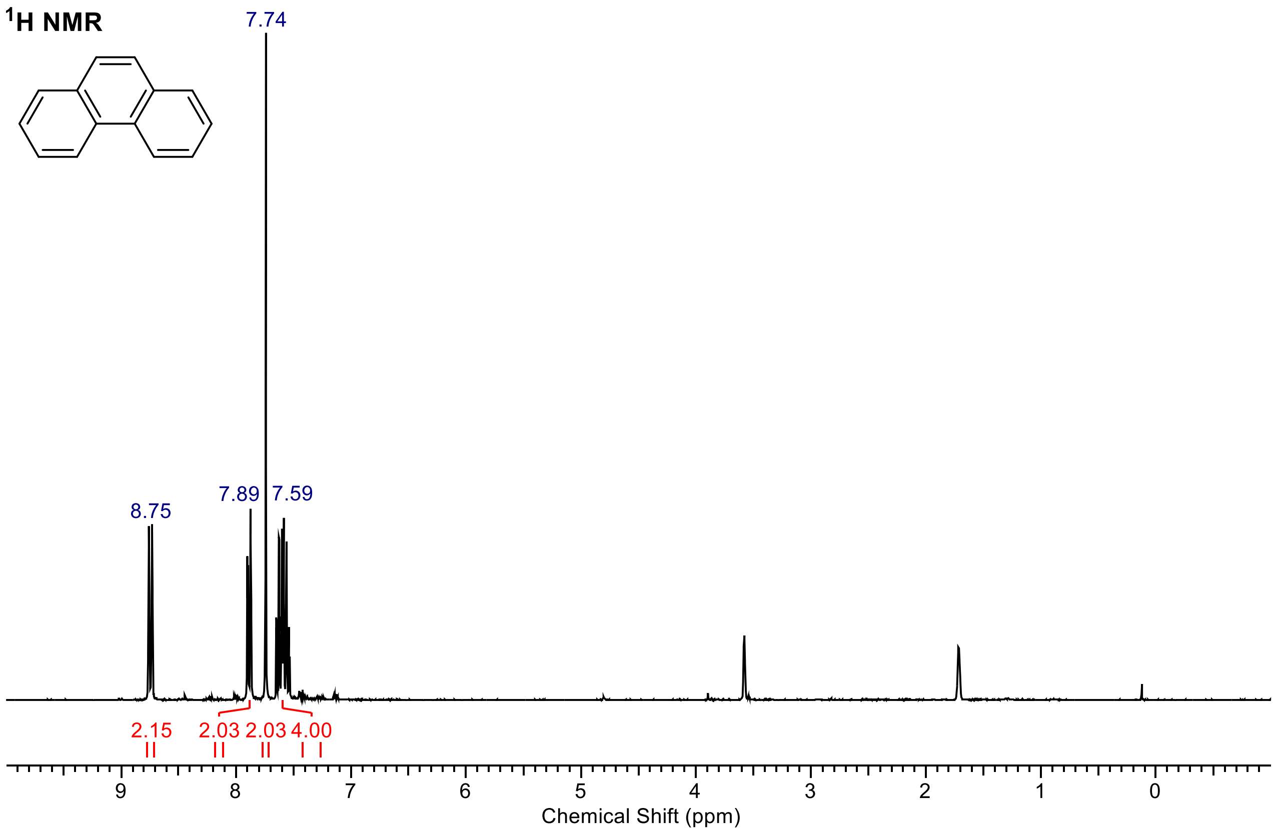


**Spectra S47:** ¹H NMR spectrum of phenanthrene in THF-$d_8$. **¹H NMR** (300.1 MHz, THF-$d_8$): δ 8.75 (dd, *J* = 7.9, 1.2 Hz, 2H), 7.89 (m, 2H), 7.74 (s, 2H), 7.66–7.53 (m, 4H).

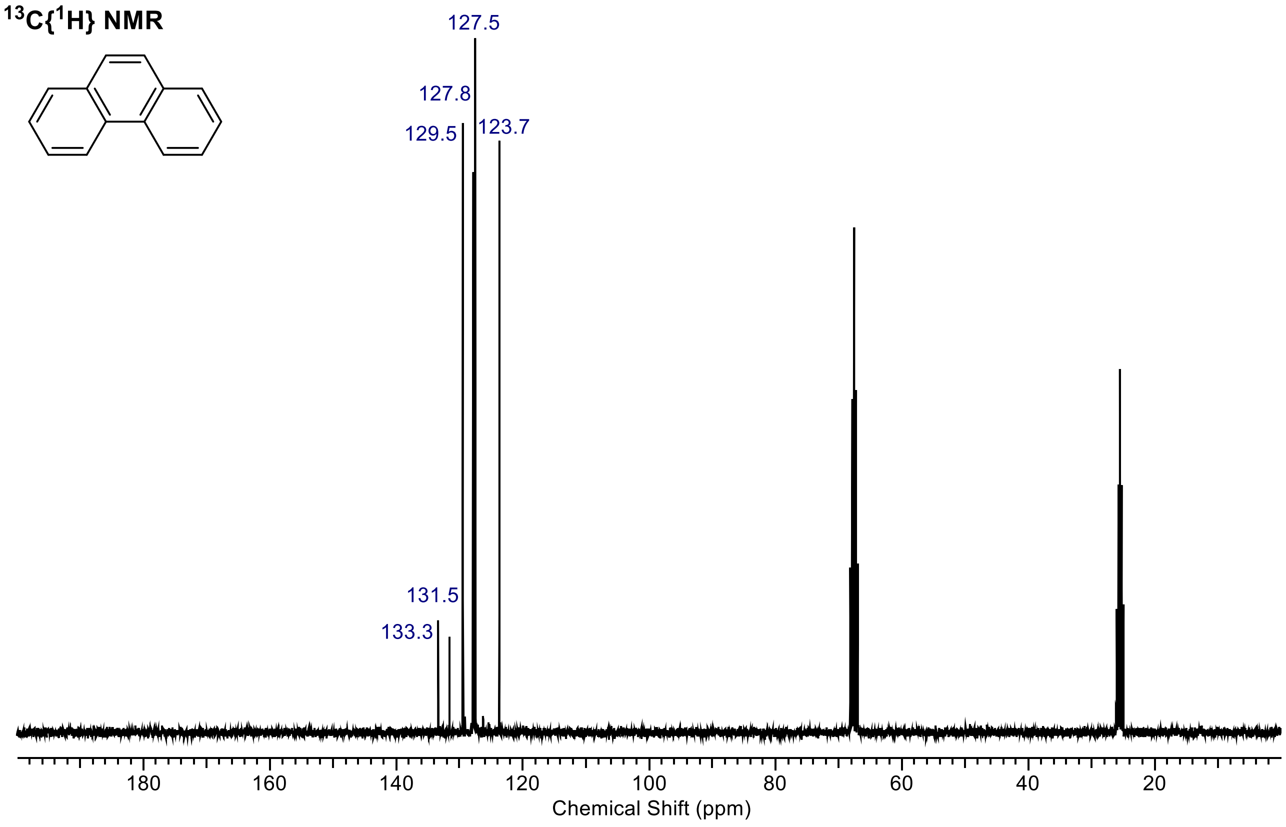


**Spectra S48:** ¹³C{¹H} NMR spectrum of phenanthrene in THF-$d_8$. **¹³C{¹H} NMR** (75.5 MHz, THF-$d_8$): δ 133.3, 131.5, 129.5, 127.8, 127.5, 127.5, 123.7.

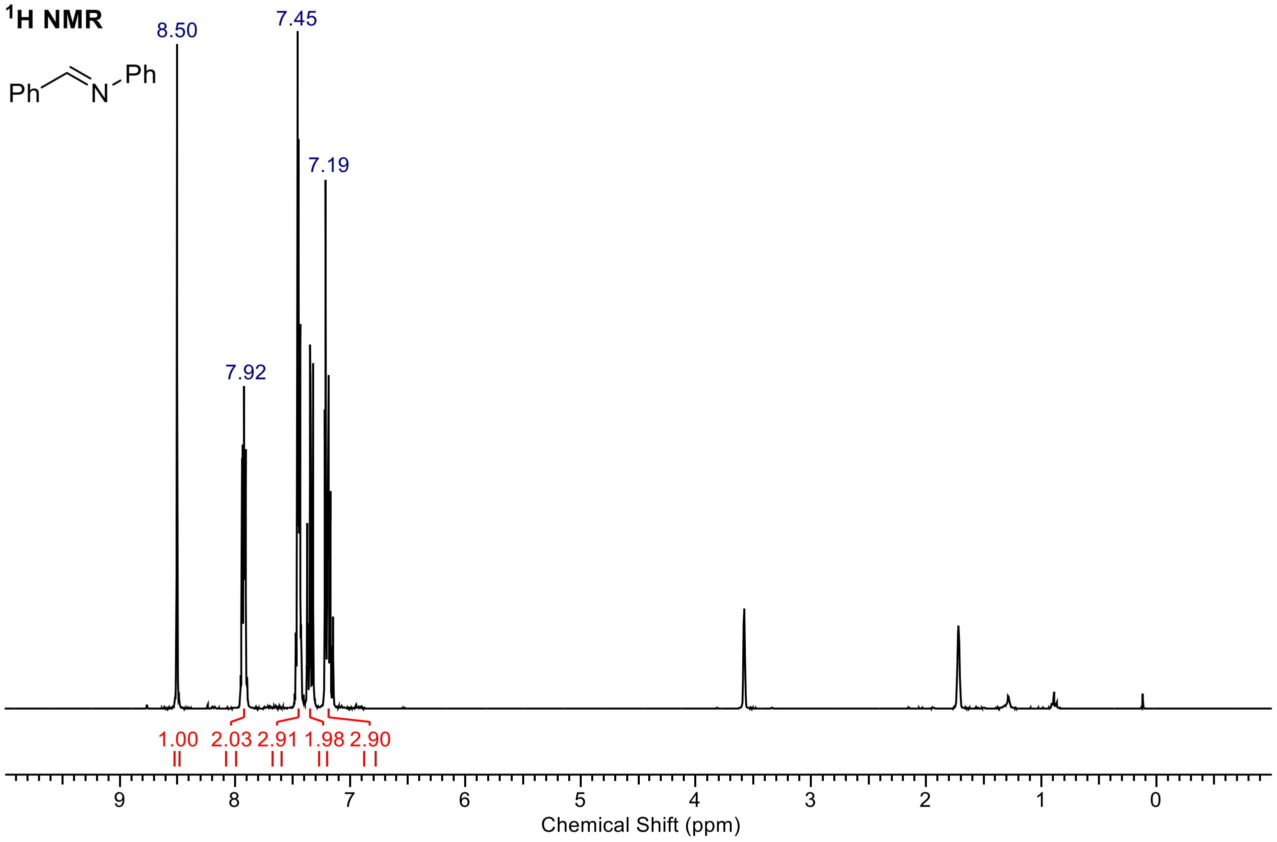


**Spectra S49:** $^{1}H$ NMR spectrum of *N*-benzylideneaniline in THF-$d_8$. **$^{1}$H NMR** (300.1 MHz, THF-$d_8$): δ 8.50 (s, 1H), 7.96–7.88 (m, 2H), 7.48–7.41 (m, 3H), 7.38–7.31 (m, 2H), 7.24–7.14 (m, 3H).

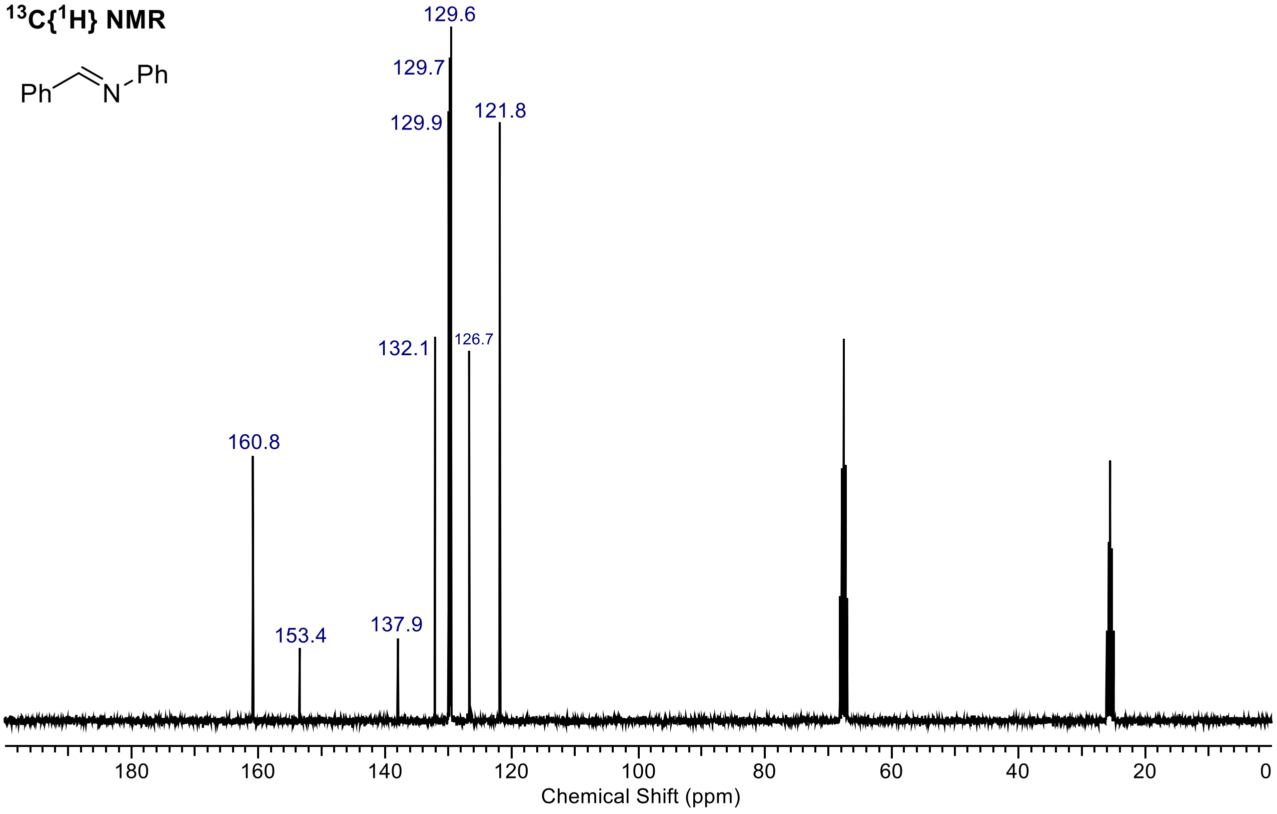


**Spectra S50:** $^{13}C\{^{1}H\}$ NMR spectrum of *N*-benzylideneaniline in THF-$d_8$. **$^{13}$C{$^{1}$H} NMR** (75.5 MHz, THF-$d_8$): δ 160.8, 153.4, 137.9, 132.1, 129.9, 129.7, 129.6, 126.7, 121.8.

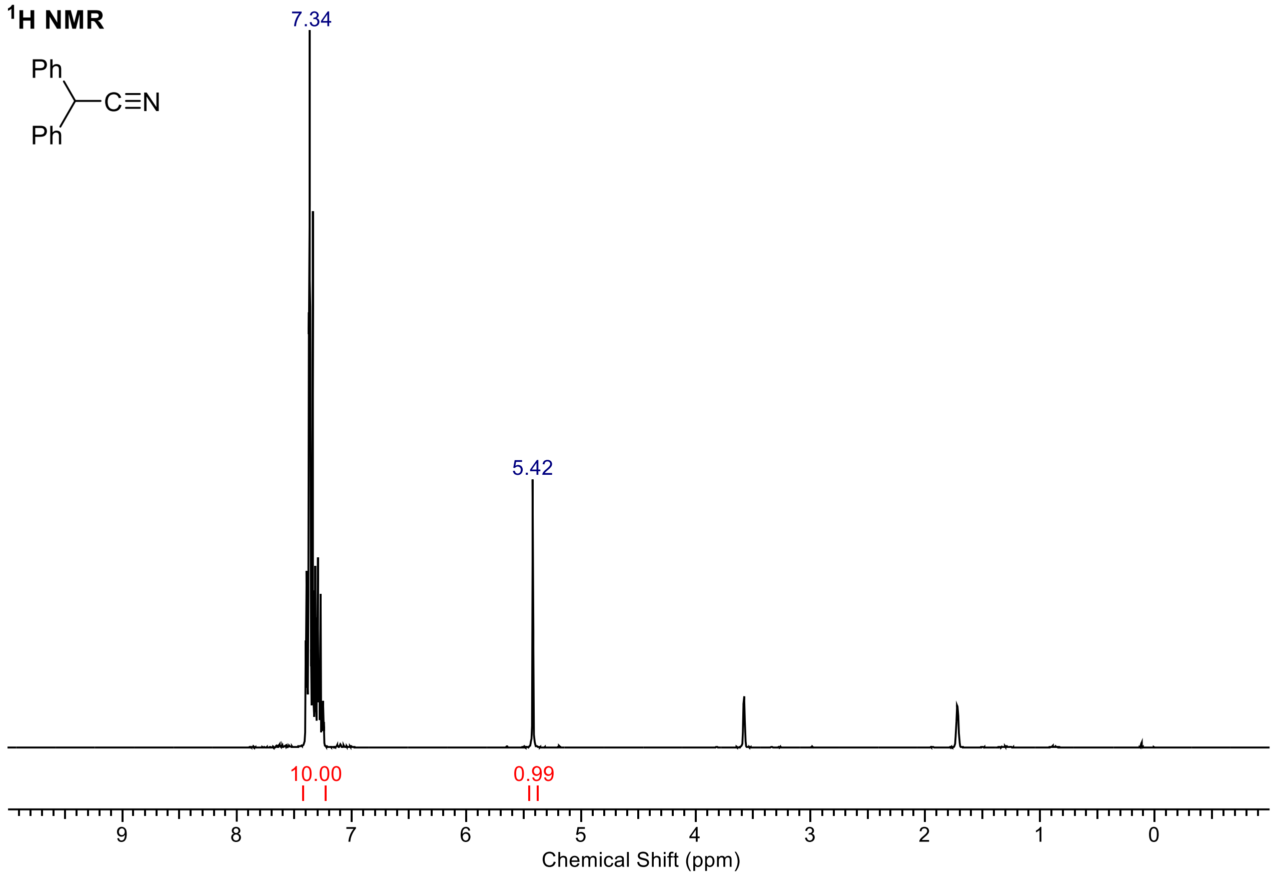


**Spectra S51:** $^{1}$H NMR spectrum of diphenylacetonitrile in THF-$d_8$. **$^{1}$H NMR** (300.1 MHz, THF-$d_8$): δ 7.41–7.24 (m, 10H), 5.42 (s, 1H).

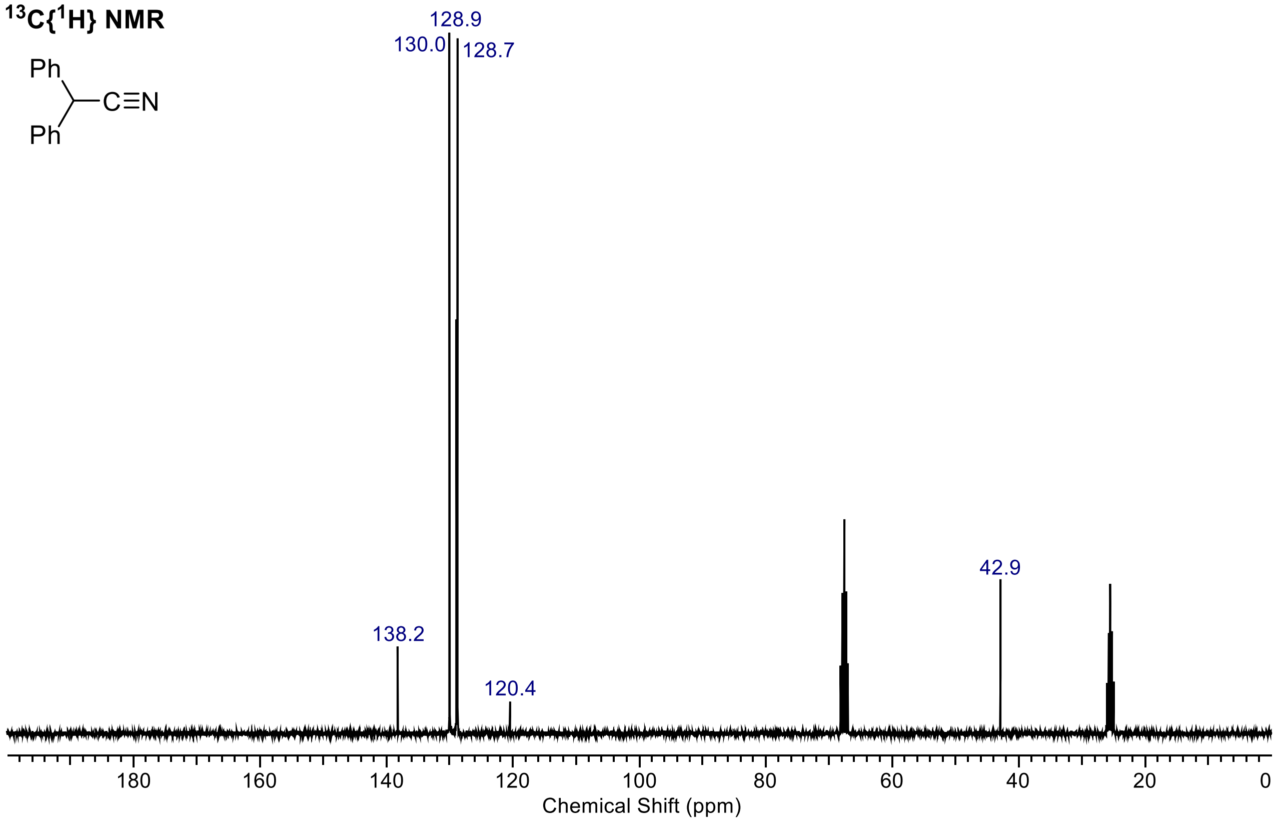


**Spectra S52:** $^{13}$C{$^{1}$H} NMR spectrum of diphenylacetonitrile in THF-$d_8$. **$^{13}$C{$^{1}$H} NMR** (75.5 MHz, THF-$d_8$): δ 138.2, 130.0, 128.9, 128.7, 120.4, 42.9.

***In Situ* Synthesis and Characterisation of Imine Addition Product, $Ph_2CHN(Li)Ph$**

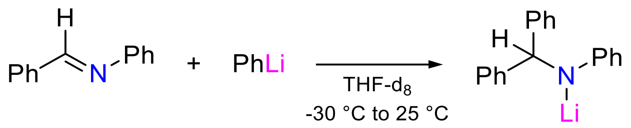


N-benzylideneaniline (18.1 mg, 0.1 mmol) was dissolved in THF-$d_8$ (0.5 mL) and cooled to -30 °C. PhLi (8.6 mg, 0.1 mmol) was added and the colourless solution was warmed to room temperature. $^1$H NMR spectroscopy indicated clean nucleophilic addition of PhLi across the C=N bond to give $Ph_2CHN(Li)Ph$.

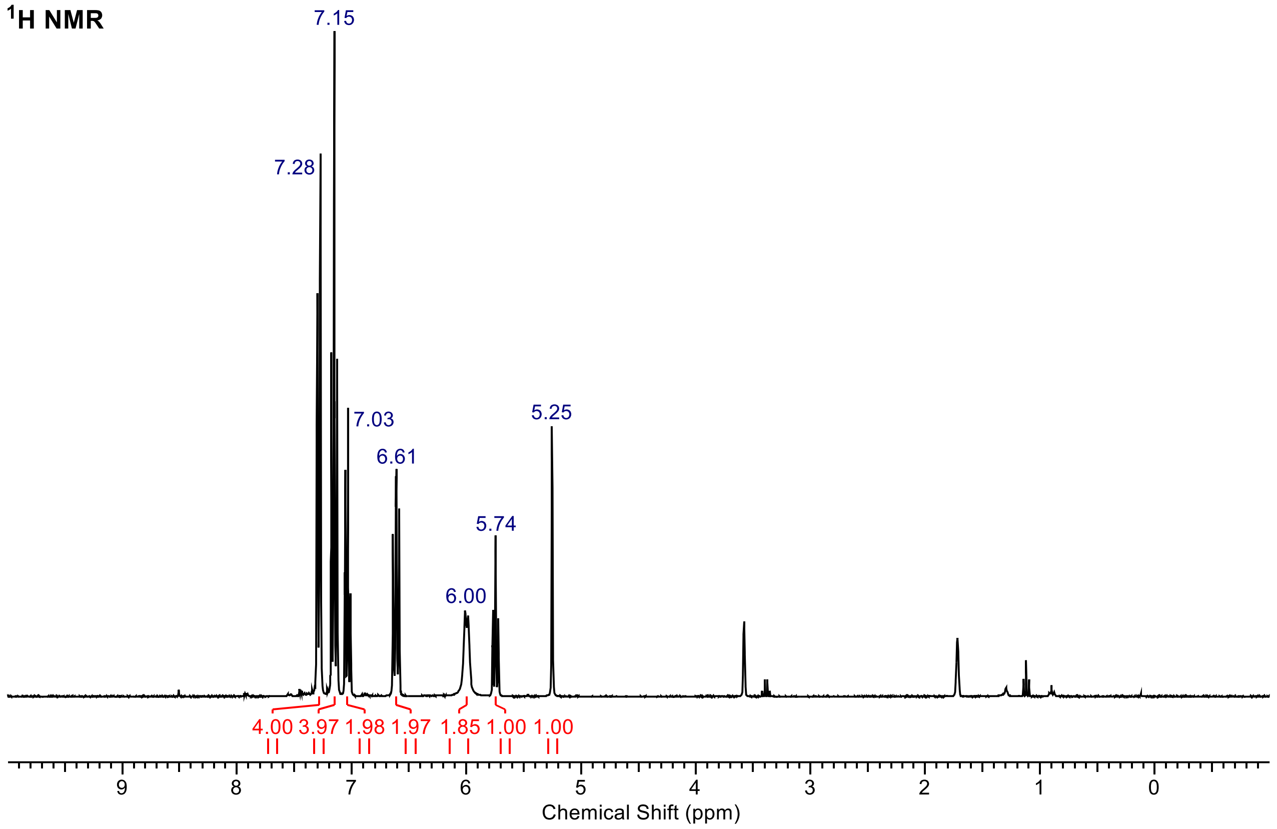


**Spectra S53:** $^1$H NMR spectrum of $Ph_2CHN(Li)Ph$ prepared *in situ* in THF-$d_8$. **$^1$H NMR** (300.1 MHz, THF-$d_8$): δ 7.28 (m, 4H, $Ph_2$-*o*-C*H*), 7.15 (t, *J* = 7.6 Hz, 4H, $Ph_2$-*m*-C*H*), 7.03 (tt, *J* = 7.2, 1.4 Hz, 2H, $Ph_2$-*p*-C*H*), 6.61 (dd, *J* = 7.1, 1.6 Hz, 2H, N-Ph-*m*-C*H*), 6.00 (br d, *J* = 7.0 Hz, 2H, N-Ph-*o*-C*H*), 5.74 (tt, *J* = 7.0, 1.0 Hz, 1H, N-Ph-*p*-C*H*), 5.25 (s, 1H, C*H*).

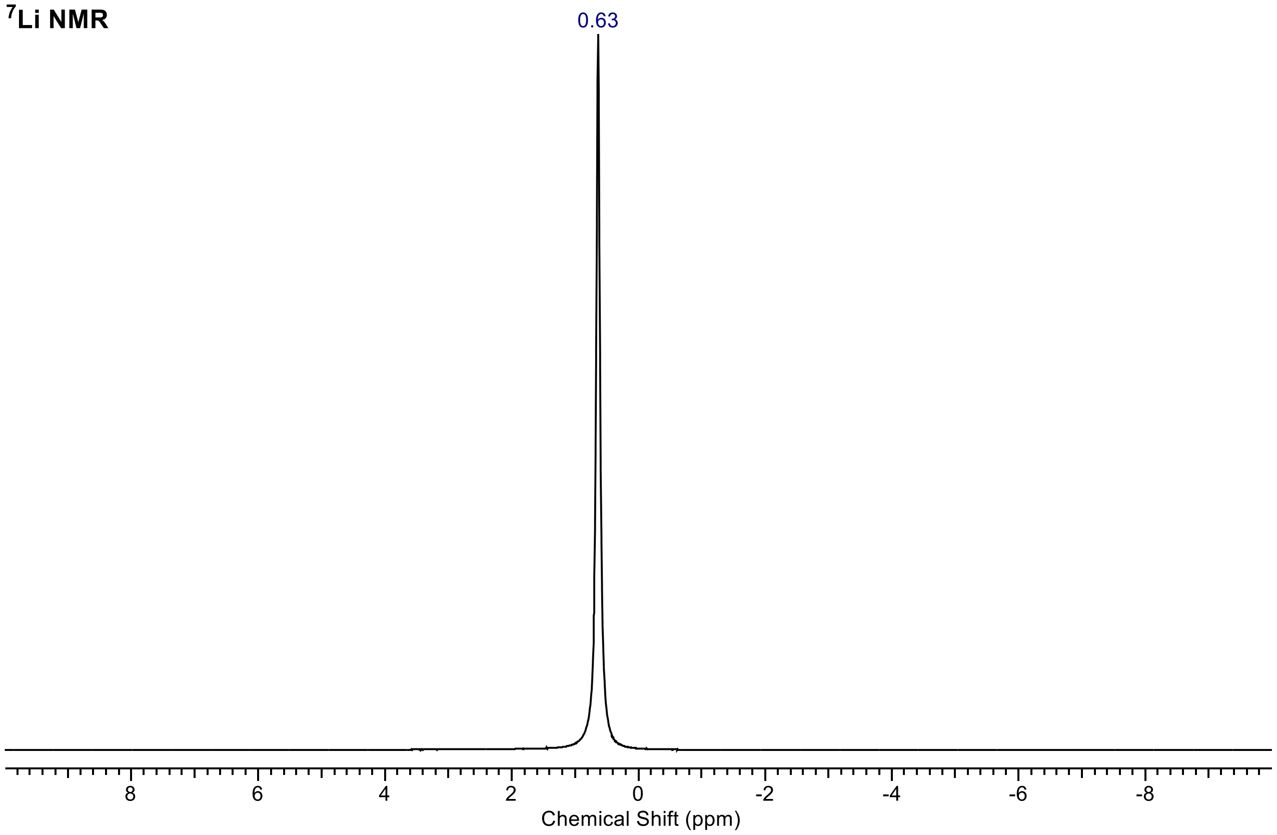


**Spectra S54:** $^7$Li NMR spectrum of $Ph_2CHN(Li)Ph$ prepared *in situ* in THF-$d_8$. **$^7$Li NMR** (116.6 MHz, THF-$d_8$): δ 0.63 (s).

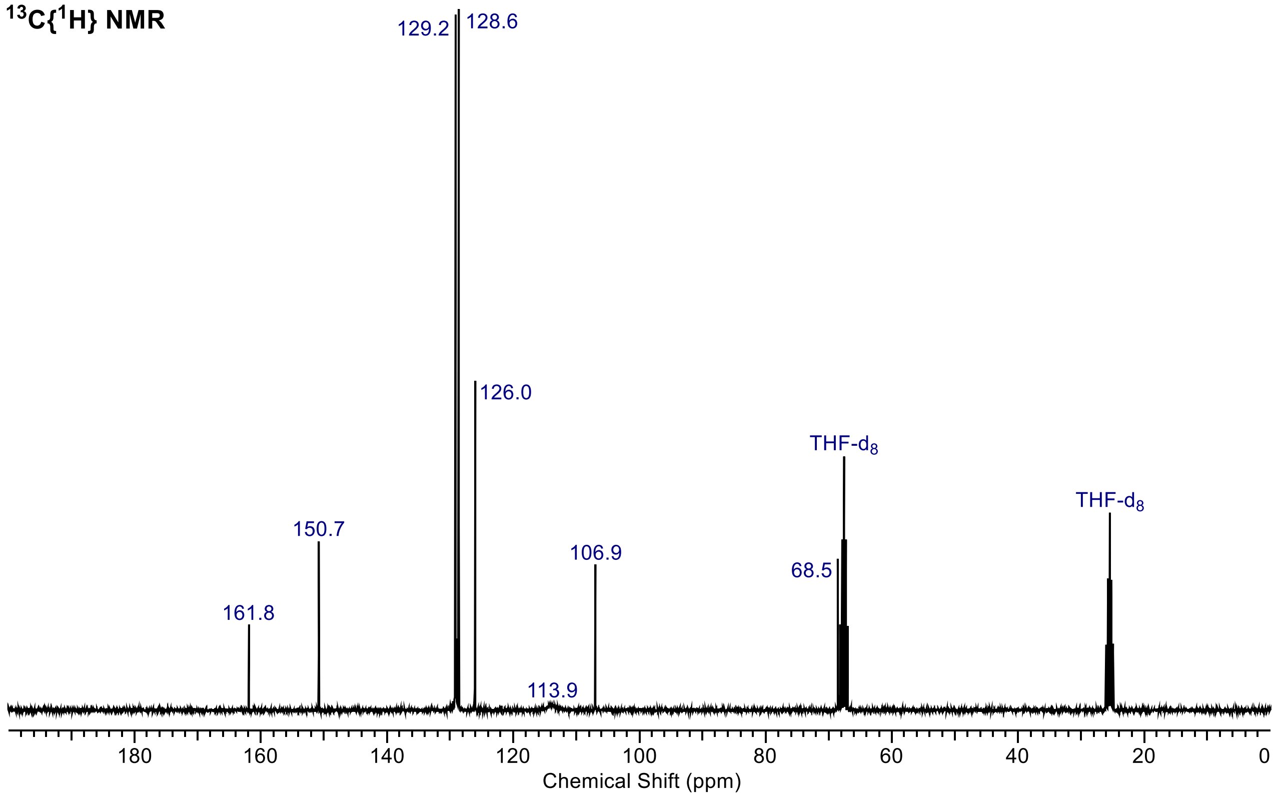


**Spectra S55:** $^{13}C\{^1H\}$ NMR spectrum of $Ph_2CHN(Li)Ph$ prepared *in situ* in THF-$d_8$. **$^{13}C\{^1H\}$ NMR** (75.5 MHz, THF-$d_8$): δ 161.8 (N-Ph-*ipso*-C), 150.7 ($Ph_2$-*ipso*-C), 129.2 ($Ph_2$-*o*-CH), 129.0 (br, N-Ph-*m*-CH), 128.6 ($Ph_2$-*m*-CH), 126.0 ($Ph_2$-*p*-CH), 113.9 (br, N-Ph-*o*-CH), 106.9 (N-Ph-*p*-CH), 68.5 (CH).

**_In Situ_ Synthesis and Characterisation of Lithiated Diphenylketenimine, $Ph_2C$=C=N–Li**

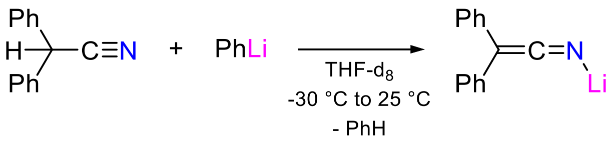


Diphenylacetonitrile (19.3 mg, 0.1 mmol) was dissolved in THF-$d_8$ (0.5 mL) and cooled to -30 °C. PhLi (8.6 mg, 0.1 mmol) was added leading to a colour change from colourless to pale yellow upon warming to room temperature. $^1$H NMR spectroscopy indicated the clean α-deprotonation to give the lithiated diphenylketenimine alongside benzene.

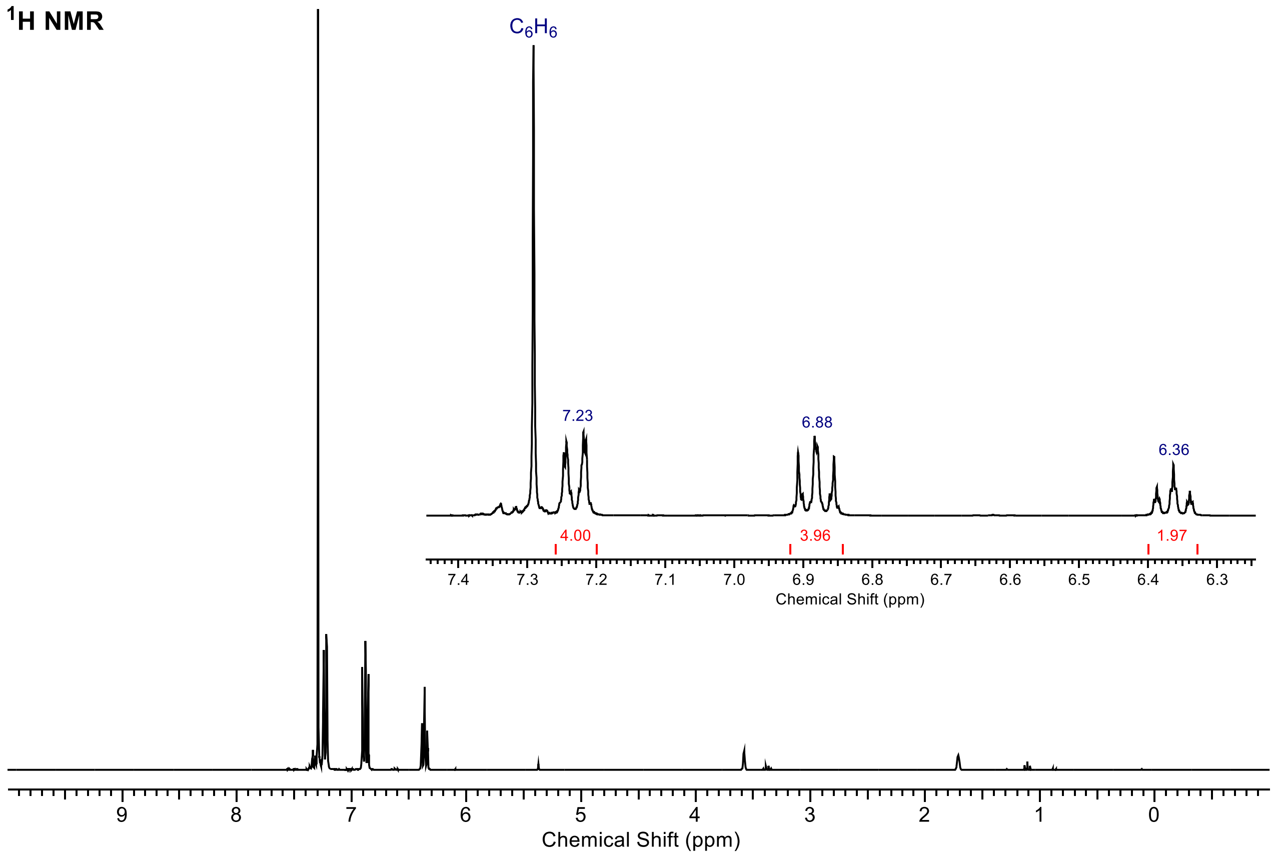


**Spectra S56:** $^1$H NMR spectrum of lithiated diphenylketenimine prepared _in situ_ in THF-$d_8$. **$^1$H NMR** (300.1 MHz, THF-$d_8$): δ 7.23 (dd, $J$ = 7.4, 1.2 Hz, 4H, Ph-_o_-C<u>H</u>), 6.88 (m, 4H, Ph-_m_-C<u>H</u>), 6.36 (tt, $J$ = 7.1, 1.2 Hz, 2H, Ph-_p_-C<u>H</u>).

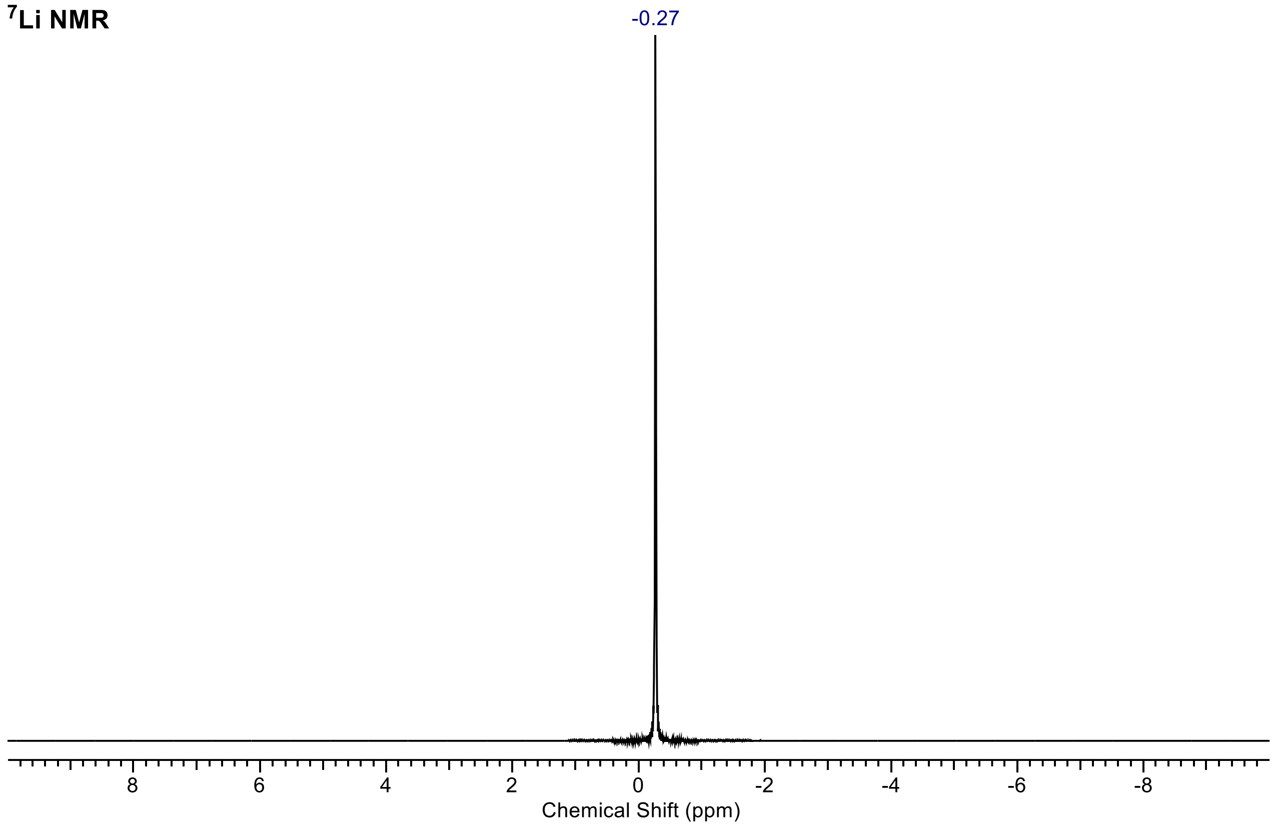


**Spectra S57:** $^{7}$Li NMR spectrum of lithiated diphenylketenimine prepared *in situ* in THF-$d_8$. **$^{7}$Li NMR** (116.6 MHz, THF-$d_8$): δ -0.27 (s).

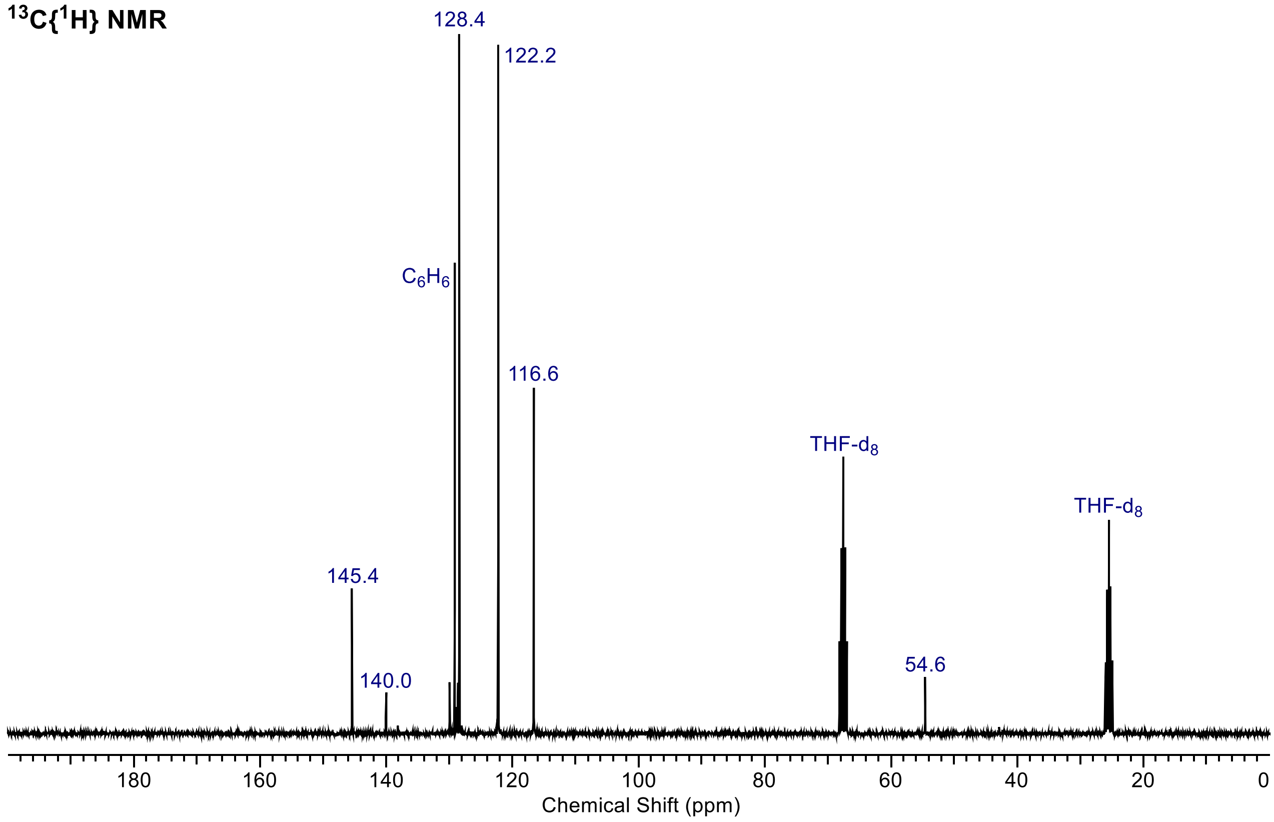


**Spectra S58:** $^{13}$C{$^{1}$H} NMR spectrum of lithiated diphenylketenimine prepared *in situ* in THF-$d_8$. **$^{13}$C{$^{1}$H} NMR** (75.5 MHz, THF-$d_8$): δ 145.4 (Ph-*ipso*-<u>C</u>), 140.0 (C=<u>C</u>=N), 128.4 (Ph-*m*-<u>C</u>H), 122.2 (Ph-*o*-<u>C</u>H), 116.6 (Ph-*p*-<u>C</u>H), 54.6 (<u>C</u>=C=N).

## References


(1) Borys, A. M. The Schlenk Line Survival Guide https://schlenklinesurvivalguide.com. (Accessed March 2024)

(2) Borys, A. M. An Illustrated Guide to Schlenk Line Techniques. *Organometallics* **2023**, *42* (3), 182–196. https://doi.org/10.1021/acs.organomet.2c00535.

(3) Bogdanović, V. B.; Kröner, M.; Wilke, G. Olefin Complexes of Nickel(0). *Liebigs Ann. Chem* **1966**, *669* (1), 1–23. https://doi.org/10.1002/jlac.19666990102.

(4) Borys, A. M.; Hevia, E. The Anionic Pathway in the Nickel-Catalysed Cross-Coupling of Aryl Ethers. *Angew. Chem. Int. Ed.* **2021**, *60* (46), 24659–24667. https://doi.org/10.1002/anie.202110785.

(5) Neufeld, R.; Stalke, D. Accurate Molecular Weight Determination of Small Molecules via DOSY-NMR by Using External Calibration Curves with Normalized Diffusion Coefficients. *Chem. Sci.* **2015**, *6* (6), 3354–3364. https://doi.org/10.1039/c5sc00670h.

(6) Bachmann, S.; Gernert, B.; Stalke, D. Solution Structures of Alkali Metal Cyclopentadienides in THF Estimated by ECC-DOSY NMR-Spectroscopy. *Chem. Commun.* **2016**, *52* (87), 12861–12864. https://doi.org/10.1039/c6cc07273a.

(7) Bachmann, S.; Neufeld, R.; Dzemski, M.; Stalke, D. New External Calibration Curves (ECCs) for the Estimation of Molecular Weights in Various Common NMR Solvents. *Chem. Eur. J.* **2016**, *22* (25), 8462–8465. https://doi.org/10.1002/chem.201601145.

(8) Oxford-Diffraction. CrysAlisPro. Oxford Diffraction 2018.

(9) Sheldrick, G. M. A Short History of SHELX. *Acta Cryst.* **2008**, *64* (1), 112–122. https://doi.org/10.1107/S0108767307043930.

(10) Sheldrick, G. M. Crystal Structure Refinement with SHELXL. *Acta Cryst.* **2015**, *C71*, 3–8. https://doi.org/10.1107/S2053229614024218.

(11) Dolomanov, O. V.; Bourhis, L. J.; Gildea, R. J.; Howard, J. A. K.; Puschmann, H. OLEX2: A Complete Structure Solution, Refinement and Analysis Program. *J. Appl. Crystallogr.* **2009**, *A42* (2), 339–341. https://doi.org/10.1107/S0021889808042726.

(12) Balashova, T. V; Polyakova, S. K.; Ilichev, V. A.; Baranov, E. V; Fukin, G. K.; Kozhanov, K. A.; Zhigulin, G. Y.; Ketkov, S. Y.; Bochkarev, M. N. Synthesis and Structure of a Samarium Complex with Perylene. *Organometallics* **2023**. https://doi.org/10.1021/acs.organomet.3c00388.
